# Determination of the crystallographic orientation of organic crystal facets with angle-resolved polarised Raman spectroscopy

**DOI:** 10.1039/d5ce00672d

**Published:** 2025-12-03

**Authors:** Dave F. Collins, Jonathan M. Skelton, Sven L. M. Schroeder, Helen Blade, Mark Jackman, Anuradha R. Pallipurath

**Affiliations:** a School of Chemical and Process Engineering, University of Leeds Leeds UK a.r.pallipurath@leeds.ac.uk; b Department of Chemistry, University of Manchester Machester UK; c Global Product Development, Pharmaceutical Technology and Development, Operations, AstraZeneca Macclesfield UK; d Business Development and Licensing, SP&A- Tech, Biopharmaceuticals R&D, AstraZeneca Cambridge UK

## Abstract

Controlling the material properties of crystalline pharmaceutical materials is essential for developing materials with robust performance and manufacturability. Identification of the crystal facets present in a material opens up the opportunity for developing strategies to control and engineer material to meet manufacturing needs. This proof of concept study presents a workflow for using powder X-ray diffraction (PXRD) and angle resolved polarised Raman spectroscopy (ARPRS), in combination with density functional theory (DFT) calculations, to identify facets in samples unsuitable for single-crystal face indexing with XRD. Using Paracetamol (PCM) form I as a model compound, we demonstrate how preferred orientation effects in PXRD can be used with ARPRS measurements at different sample orientations, obtained by rotating in the plane perpendicular to the laser incidence direction, to define facet assignments from a set of possible planes. PXRD alone cannot distinguish the (011) and (01̄1) facets, but these can be differentiated with ARPRS by analysing the change in normalised band intensity of selected vibrational modes under crystal rotation. Information on the symmetry and orientation of vibrational modes relative to the incident laser can be related to the orientation of functional groups, and this information is consistent with the predicted particle morphology as well as with measurements of the interfacial angle between the facets and corresponding Miller planes.

## Abstract 2

This paper presents a correlative workflow for the identification of symmetry equivalent organic crystal facets using angle resolved polarised Raman spectroscopy.

## Graphical abstract

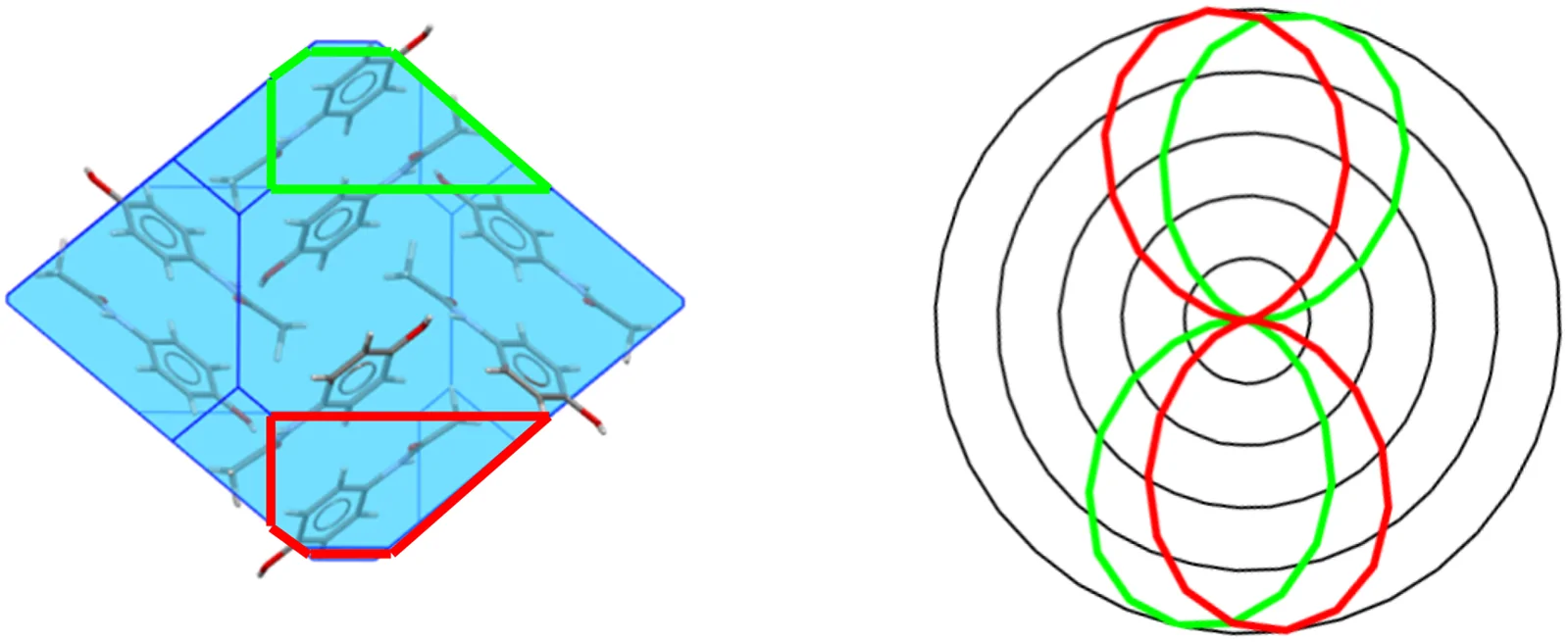

## Introduction

Crystal engineering is a topic of much interest to the pharmaceutical industry, with the aim of manipulating the solid-state structure of materials to “design in” bulk properties.^1^ The performance and manufacturability of a drug product is determined by the interfacial interactions between the active pharmaceutical ingredient (API) and excipient materials in the formulation.^2,3^ Performance of a pharmaceutical material is also tied to the crystal habit, with elongated needle like particles undesirable due to their poor flowability.^4^ Particles of this morphology also pose a challenge in downstream processing and scale up, with needle shaped paracetamol particles for example demonstrating a greater propensity to segregate and form tablets with greater friability compared to those sourced from more granular material.^5^ Currently there is a lack of tools available to formulation scientists to predict both crystal facet specific properties and potential particle-particle interactions in real-world samples.^6^ As a result, there is an interest in controlling these properties by preferentially forming desirable crystal habits early in the manufacturing process.^7^

The morphology of crystalline materials is characterized by crystallographic faces, indexed by Miller indices, with different faces often exhibiting significant differences in physicochemical properties.^8^ Theoretical models that predict crystal morphologies based on the crystal planes with the lowest attachment energies^9^ have come a long way in aiding particle science, and the logical next step is to improve our understanding of the chemical nature of these crystal facets. However, there is currently a lack of understanding of how crystal structure, notably surface speciation, links to physicochemical properties.^10^ This is particularly true for organic systems, due to the complex influences of low crystal symmetry and solvent-dependent functional group terminations on the surface structure.

Facets of organic crystals generally exhibit a complex range of potential surface terminations, exposing different functional groups to the local environment and leading to diverse and facet-dependent chemical behaviour.^10^ Studies on mefenamic acid and celecoxib demonstrate that particle surfaces that expose electronegative^11^ and polar functional groups^12^ to a tablet punch show a greater propensity to stick during tablet compression. Similarly, the difference in potential interactions between surface functional groups and solvent molecules can be exploited to alter the needle like crystal habit of 5-aminosalicylic acid crystals and obtain material with more desirable properties.^7^ The interactions between surface functional groups and solvent droplets have been directly observed in aspirin crystals using polarised Raman spectroscopy (PRS) through the presence of peaks characteristic of interactions between the crystal surface and adsorbed solvent molecules.^10^ Despite most Raman spectrometers lacking the spatial resolution required for surface sensitive measurements, PRS can provide information to determine crystal facets and orientations that can be related to, and used to probe deviations from, the expected bulk crystal structure.

Facet identification is typically performed using single crystal X-ray diffraction (SCXRD). This technique does not provide insight on the surface structure and orientation of functional groups outside of the crystallographic bulk model and is only suitable for samples with sufficient crystallinity and scattering quality for analysis,^13^ with facet identification possible on poorer quality crystals as long as the crystal structure is known. As an alternative, powder XRD (PXRD) is typically used to identify the polymorphic form of a material, where characteristic Bragg peaks corresponding to the Miller indices of the facets in the incident X-ray beam can be measured. As this technique does not have the same practical sample limitations as SCXRD, it can be used to detect Bragg peaks characteristic of a single facet present in a crystal sample, provided the sample height is corrected for.^14^ However, as the Bragg peak positions are determined by the *d*-spacing, this approach cannot distinguish between symmetry-equivalent planes with identical or similar *d*-spacing without prior knowledge of the absolute orientation of the sample. This is a particular issue for centrosymmetric crystal systems such as the *P*2_1_/*a* structure of paracetamol form I (PCM-I), where the (011), (01̄1), (011̄) and (01̄1̄) facets have identical *d*-spacing and are indistinguishable using PXRD alone, preventing observations of facet behaviour that deviates from the bulk structure.

A variety of other analytical techniques have been used to study facet-dependent behaviour, each with their own advantages and disadvantages. X-ray photoelectron spectroscopy (XPS) has been used to study H-bonding networks and charge transfer in multicomponent systems,^15,16^ but using it to definitively identify crystal facets has proven challenging.^17^ XPS is highly sensitive to surface cleanliness, phase purity and local structure, since about 28% of the spectral information arises from a depth up to 10 Å below the surface.^17^ This means that XPS is highly sensitive to adventitious carbon and/or other impurities that are often present on crystals produced by solution crystallisation.^17^ The surface sensitivity of XPS therefore complicates measurements of crystal surface properties, as these are not solely determined by the “native” functional groups exposed by the crystal structure. This further highlights the need for a complementary technique to characterise deviation from the bulk. Time of flight secondary ion mass spectrometry (TOF-SIMS)^18^ has the capability to yield chemical information in two or three dimensions with high sensitivity and mass resolution,^18^ and has been used to analyse the distribution of 4-nitrophenol impurities present at low concentration on the surface of PCM single crystals,^19^ but the method is destructive by nature.^20^ Atomic force microscopy (AFM) measurements have also been used to demonstrate that PCM-I particle monolayers on a silica surface form as a randomly oriented powder, whereas PCM form II and III (PCM-II/PCM-III) form as aligned crystallites. This was supported by the presence of Bragg spots in grazing incidence X-ray diffraction (GIXD) measurements of PCM-II and PCM-III compared to diffraction rings for PCM-I.^21^ However, while AFM has emerged as a common method for measuring the physicochemical properties of specific facets,^22^ it does not provide chemical information, and etching from the probe tip can damage soft samples.^23^

Raman spectroscopy is a fast and non-destructive characterisation technique^24^ that can provide information specific to the structure and chemical composition of a material. Facet specific Raman measurements (particularly angle resolved) are less frequently seen in literature than bulk powder measurements, and are difficult to predict from the bulk crystal structure. As such, a computational approach is required to predict the Raman behaviour, exploiting the natural polarisation of the incident laser, and utilising libraries of Raman spectra to experimentally determine the morphology of powder samples. For example, Wijethunga *et al.* developed machine learning models based on Raman scattering from single crystal facets of PCM between 400–1200 cm^−1^ to identify unknown crystal facets from epitaxially grown crystals,^25^ and validated the predictions against traditional SCXRD face indexation. Notably, face indexation is complicated in epitaxially grown crystals, requiring the use of the Kullback–Leibler divergence method to identify symmetry equivalent crystal faces, which clearly highlights the need for simpler alternatives.

**Fig. 1:**
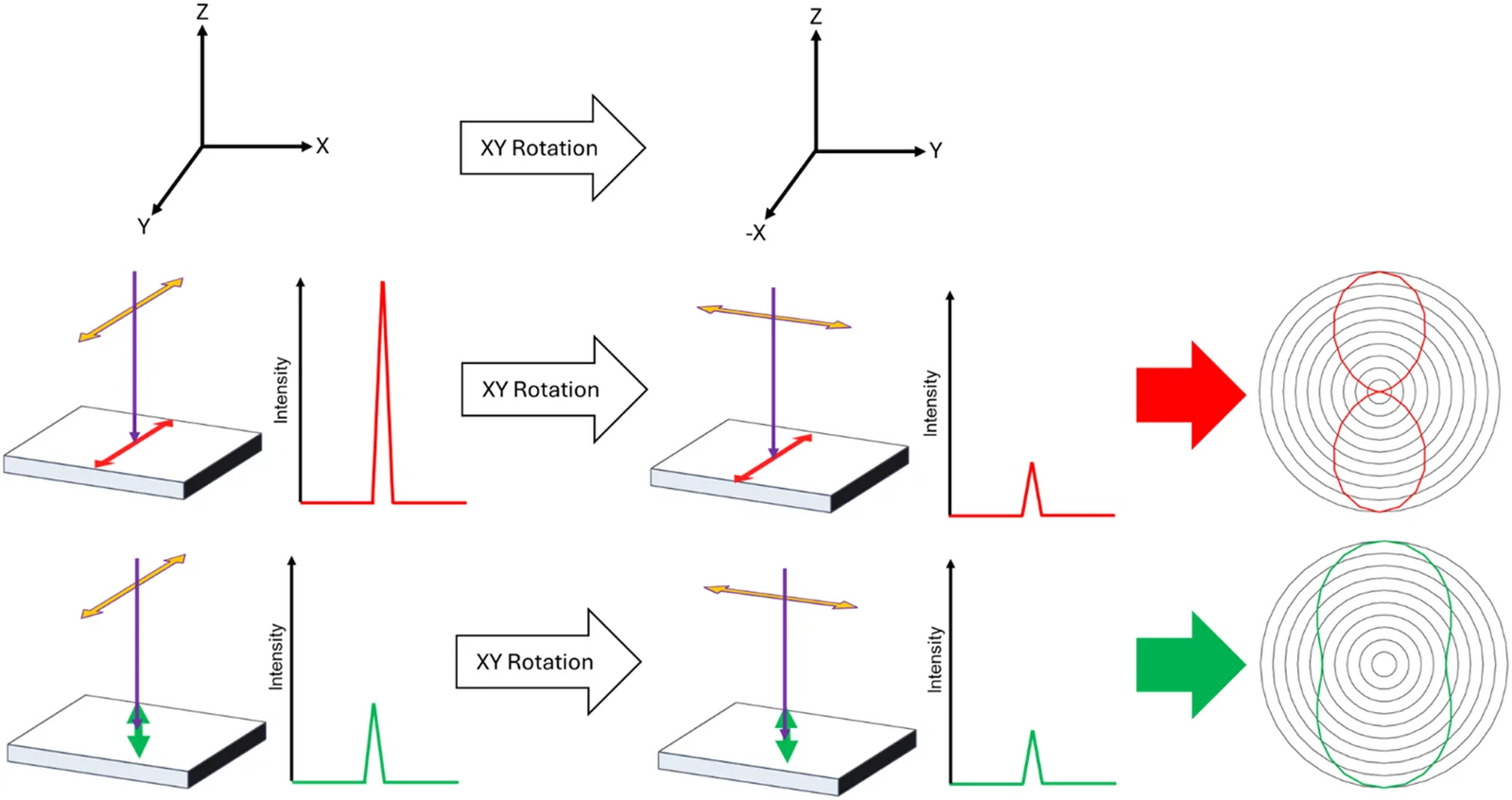
Schematic illustration of the impact of the orientation of the vibrational mode displacement vector relative to incident laser polarisation on observed Raman intensity and the resulting angle-resolved band intensity polar plots.

The polarised nature of Raman measurements can be further exploited in angle resolved PRS (ARPRS) measurements. ARPRS measurements involve the use of a polarised incident light source, typically in a backscattering geometry, to evaluate the change in Raman intensity as a function of the sample rotation angle to probe the polarisation dependence of spectral bands (Fig. 1).^26^ Previous studies of organic materials, such as on β-carotene crystals, have exploited the relationship between the intensity of the scattered Raman signal and the orientation of the Raman tensor relative to the polarisation of the incident light^27^ and the direction of observation.^28^ ARPRS measurements are highly versatile and have been used for a number of applications. The technique is widely used to study two-dimensional materials such as MoS_2_,^29^ as well as organic, polarisation sensitive photodetector materials such as 2,6-diphenyl anthracene.^30^ ARPRS has also been used to deduce the absolute orientation of ReSe_2_ ^31^ flakes and carbon nanotubes on silica substrates,^32^ as well as to identify phase coexistence in rubrene single crystals through changes in the phonon bands with laser polarisation, using density functional theory (DFT) calculations as a reference.^33^ Similar to the latter example, ARPRS has also be used to probe the symmetry of hydrogen bonding networks in the phonon region of l-alanine single crystals.^26^

An improved understanding of the nature of Raman vibrational modes at specific facets, as a function of the orientation of organic crystals, could allow for both straightforward identification of crystal facets and determination of absolute crystal orientation. Furthermore, it may in principle be possible to determine the orientation of functional groups relative to the crystal surface, providing links between the possible surface chemistry and its impact on physicochemical properties.

In this paper, we build on these promising preliminary findings and use ARPRS to enable the differentiation of symmetry equivalent crystal facets. We present a workflow that combines ARPRS, PXRD, interfacial angle measurements and reference DFT calculations to assign individual crystal facets. We demonstrate this approach on model PCM-I crystals grown from methanol. PCM is commonly used as a model API for technique development, as its crystal structure and physicochemical properties, including those related to the manufacturability and final drug product performance, are well characterised. This work serves as a prelude to identifying the surface terminations and absolute orientations of the functional groups on organic crystals *e.g.* for optimising downstream processing.

## Methodology

Our work is organised as follows. We first outline the methods used in our workflow, illustrated in Fig. 3, which exploits preferred orientation effects in PXRD to initially assign major facets, and uses ARPRS measurements supported by DFT simulations to further distinguish between symmetry equivalent faces. We then present and discuss the results of a proof-of-concept application to PCM-I crystals. Finally, we validate our facet assignments by measuring the interfacial angles using tensiometry and cross referencing to the angles between the corresponding Miller planes.

### Crystal growth

**Fig. 2:**
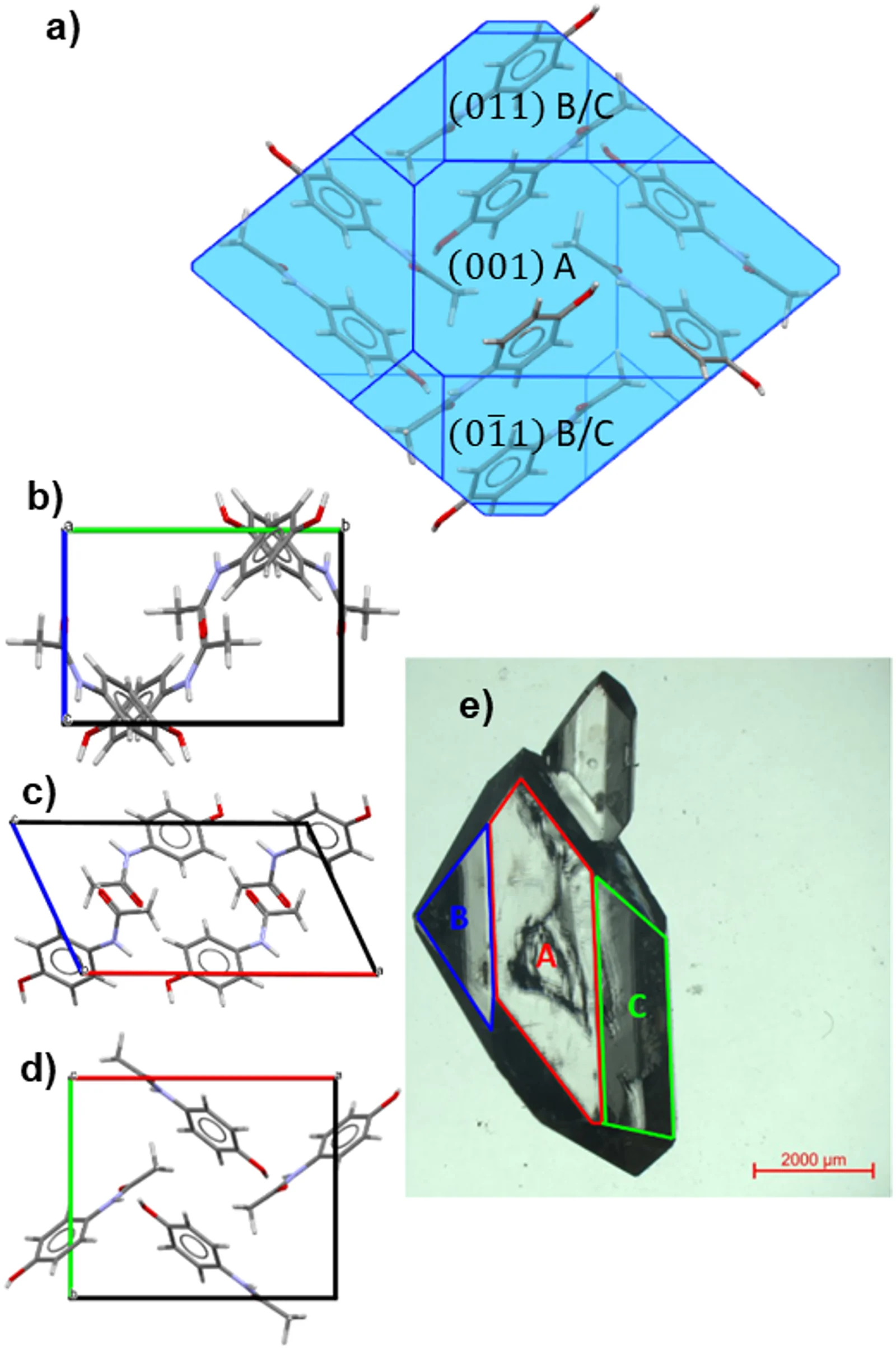
(a) Crystal morphology of PCM-I predicted using visual HABIT with the CCDC Mercury software, with the major crystal facets labelled.^49^ (b)–(d) Crystal structure of Paracetamol form I (PCM-I, CCDC: **HXACAN01** (ref. 40)) viewed along the *a* (b) *b* (c) and *c* axes (d). (e) Optical bright field micrograph of a large PCM single crystal grown from methanol.

Seed crystals of PCM-I were grown *via* slow evaporation from a 0.3 g mL^−1^ methanol solution for one week in a 20 mL glass vial, yielding crystals with a long axis of approximately 150 μm (PCM: Sigma-Aldrich, 98–100%, MeOH: VWR Chemicals, 99+% extra pure L). Five of these crystals were then used to seed a second, slower evaporative crystallisation from 0.3 g mL^−1^ methanol solution in fresh 20 mL glass vials left to evaporate for 3 months, yielding PCM crystals with long axes between 3–6 mm and large enough facets for ARPRS and PXRD analysis. All five crystals exhibited a dominant facet, A, flanked by two angled facets B and C, as shown in Fig. 2e. Bright and dark field microscope images of the five large crystals, with the dominant facets indicated, are provided in Fig. S1 in the SI.

### Powder X-ray diffraction

PXRD measurements of the large single crystals were performed on a Bruker D8 instrument, with the crystal facet flush with the top of a Perspex sample holder and aligned with the incoming beam. A wooden platform was used to tilt the crystal to access the (011) and (01̄1) angled facets, as shown in Fig. S2. All measurements were performed using a Cu Kα source (*λ* = 1.5406 Å) and a VANTEC-1 detector, with a 2*θ* range of 10–40°, a step size of 0.01649°, and an exposure time of 0.38 s per step.

The large PCM crystals are too large to face index using SCXRD. Instead, we exploited preferred orientation effects in PXRD measurements and fit the peaks to theoretical scattering patterns for the HXACAN01 structure (CCDC: **HXACAN01**). These patterns were generated using the CrystalDiffract software,^34^ based on Lorentzian lines shapes and plate-like crystals with facet specific preferred orientations selected to match the large surfaces observed on the crystals.

### Angle resolved polarised Raman spectroscopy

Raman spectra were collected using a Renishaw inVia Raman microscope with a CCD detector and 532 nm green laser in a backscattering geometry (laser propagation along the *z* direction in the laboratory coordinate system and polarised along the *x* direction). Spectra were collected between 30–3500 cm^−1^ with 0.5 mW laser power, 10 s exposure time, three accumulations per spectrum, and a 10× objective producing a laser spot size of approximately 7 μm^2^. For ARPRS measurements, a series of spectra were collected with the sample rotated by 20° in the laboratory *xy* plane between each measurement. A fixed visible light 2400 lines mm^−1^ diffraction grating was used during data collection as this experimental setup demonstrated clear polarisation behaviour. Polarisation behaviour is known to be sensitive to the groove density on the diffraction grating, as this affects the reflectivity, and care must be taken when considering data from different instruments for comparison. A comparative study is beyond the scope of the current work.

To maximise the measured Raman intensity and to aid comparison between samples, the measured facets must be flat (*i.e.*, orthogonal to propagation direction of the laser) to avoid introducing additional angle dependencies. All facets were therefore tilted as needed and rested on the bespoke wooden frame shown in Fig. S2. To improve comparability between measurements of different facets baseline correction was performed on each scan after collection, in addition to cosmic ray removal to remove background signals, using the Renishaw Wire software. Vibrational modes were assigned by comparing the measured spectra to literature reports^35,36^ and DFT simulations of the spectra of the **HXACAN01** structure. A full comparison and assignment of the major spectral features is provided in Table S1.

To process the Raman data, a Python script was written to extract band intensities *I*_*j*_ from individual scans at each sample orientation angle and perform a min-max normalisation based on the intensity of the tallest peak in the range 30 < *ν* < 3500 cm^−1^ across all sample rotations *ϕ* (eqn (1)):1
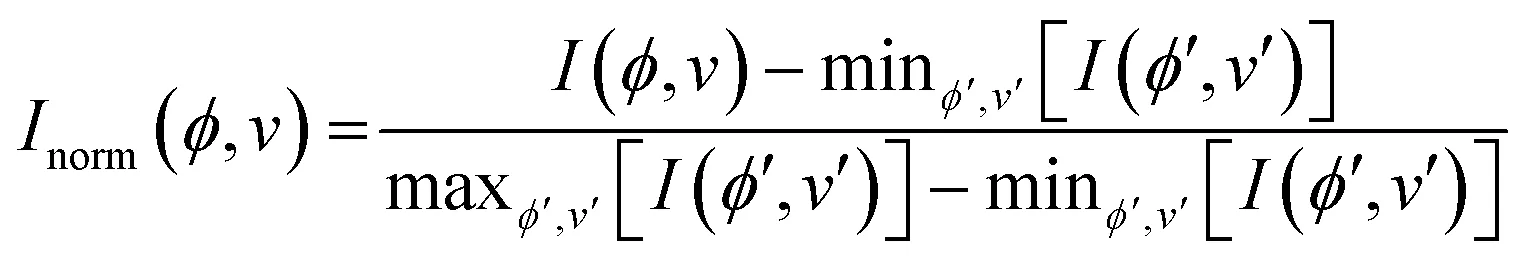


The angle dependence and polarisation of the vibrational modes can be described by both the normalised intensity range Δ_*j*_ and the depolarisation ratio *ρ*_*j*_ calculated using eqn (2) and (3) respectively:

2 Δ_*j*_ = max_*ϕ*_[*I*^Norm^_*j*_(*ϕ*)] − min_*ϕ*_[*I*^Norm^_*j*_(*ϕ*)]

3 *ρ*_*j*_ = min_*ϕ*_[*I*^Norm^_*j*_(*ϕ*)]/max_*ϕ*_[*I*^Norm^_*j*_(*ϕ*)]

The Δ_*j*_ reveal information about the degree of selective excitation of the vibrational mode, while the *ρ_j_* indicate the symmetry of the scattering from the vibrational mode. Assuming the vibrations being compared have the same scattering symmetry, a greater Δ_*j*_ indicates the vibrational mode has a greater dependence on the sample orientation *ϕ*. In the case of most vibrational modes, a lower *ρ*_*j*_ demonstrates a more polarised vibrational mode, although this interpretation is complicated for vibrational modes that are equally polarised in orthogonal directions.

Polar plots showing the angle dependence of the band intensities at each facet were created by aligning the maximum *I*^Norm^_*j*_ of the OH wag to *ϕ* = 0°. An equivalent alignment procedure was performed with the simulated *I*_*j*_ (*ϕ*) for comparison to the experimental data. Each polar plot was constructed by then taking an average of the *I*^Norm^_*j*_ across all five samples (15 scans total).

### *Ab initio* Raman simulations

Raman spectra were simulated using pseudopotential plane-wave density-functional theory (DFT) as implemented in the Vienna *ab initio* simulation package (VASP) code.^37^

The starting point for our calculations was the published 80-atom unit cell taken from the Cambridge Structural Database (CCDC: **HXACAN01**).^38^ Calculations were performed using the PBE generalised-gradient approximation (GGA) functional with the DFT-D3 dispersion correction (*i.e.* PBE + D3).^39,40^ The ion cores were modelled with projector augmented-wave (PAW) pseudopotentials,^39,41^ with the H 1s and C, O and N 2s/2p electrons in the valence region. Explicit convergence tests indicated that a plane-wave cut-off of 850 eV and a Γ-centered Monkhorst–Pack ***k***-point sampling mesh^42^ with 1 × 1 × 2 subdivisions were sufficient to converge the absolute total energy and external pressure to <1 meV atom^−1^ and 1 kbar (0.1 GPa) respectively. We subsequently found that using a single ***k***-point, *i.e.* a 1 × 1 × 1 mesh containing ***k*** = Γ, yielded practically identical optimised structures and phonon frequencies at a significantly lower computational cost. The precision of the charge-density grids was set automatically to avoid aliasing errors, the PAW projection was performed in reciprocal space, and non-spherical contributions to the gradient corrections inside the PAW spheres were accounted for.

The experimental structure was optimised with the unit-cell parameters fixed to the experimental values (*a* = 12.934 Å, *b* = 9.401 Å, *c* = 7.102 Å, *α* = *γ* = 90°, *β* = 115.9°) and tolerances of 10^−8^ eV and 10^−2^ eV Å^−1^ were applied to the electronic total energy and ionic forces, respectively, during electronic-structure calculations and geometry optimisations.

Phonon calculations were then performed on the optimised structures using the finite-differences approach implemented in the Phonopy package with a step size of 10^−2^ Å.^43^ Since simulating the Raman spectra only requires the modes at a single phonon wavevector ***q*** = Γ, we performed the phonon calculations with single unit cells. However, we also performed an additional calculation on an expanded 2 × 2 × 2 supercell to evaluate the phonon dispersion and density of states (DoS) and confirm the absence of imaginary harmonic modes (Fig. S10).^44^ Our Phonopy-Spectroscopy package^45^ was then used to determine the Raman-activity tensors ***α***_Γ*j*_ of the Γ-point phonon modes according to:4
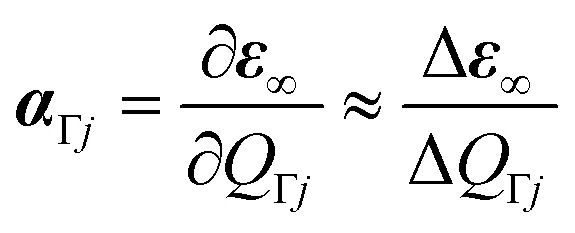
where ***ε***_∞_ is the high-frequency dielectric constant and *Q*_Γ*j*_ is a displacement along the *j*th Γ-point mode. In this work, we used a two-point finite difference stencil with a normal-mode displacement step Δ*Q* = 3.5 × 10^−2^ amu^1/2^ Å and calculated the ***ε***_∞_ using the density-functional perturbation theory (DFPT) routines in VASP^46^ and a denser ***k***-point sampling mesh with 2 × 3 × 5 subdivisions compared to that used for the optimisation and phonon calculations. We note that we performed careful testing to verify the ***k***-point sampling and method used to calculate the ***ε***_∞_, and further details are given in section S1 of the SI. During the single-point force and ***ε***_∞_ calculations an additional support grid with 8× the number of points as the standard grid was used to evaluate the augmentation charges to minimise numerical noise.

The (scalar) band intensities *I*_Γ*j*_ of the Γ-point phonon modes were calculated as:5
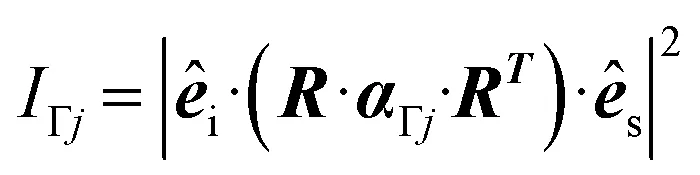
where *j* is the band index, ***ê***_i_ and ***ê***_s_ are the unit polarisation vectors of the incident and scattered light, and ***R*** is a rotation matrix that rotates the crystal into a measurement reference frame corresponding to a backscattering geometry with the incident light directed along *z* and the scattered light collected along *z̅*. The ***R*** are obtained by compositing two rotation matrices ***R*** = ***R***_*ϕ*_***R***_*hkl*_. ***R***_*hkl*_ aligns the surface normal ***n̂***_*hkl*_ of the crystal surface with Miller indices *hkl* antiparallel to the incident direction ***k***_i_, and is obtained from a *θ* = 180° rotation about the vector ***k*** = (***n̂***_*hkl*_ + ***k***_i_)/2 using Rodrigues' formula:

6 ***R***_*hkl*_ = ***I*** + ***K*** sin π + ***K***^2^(1 − cos π) = ***I*** + 2***K***^2^

where ***I*** is the identity matrix and ***K*** is calculated from the vector ***k*** as:7
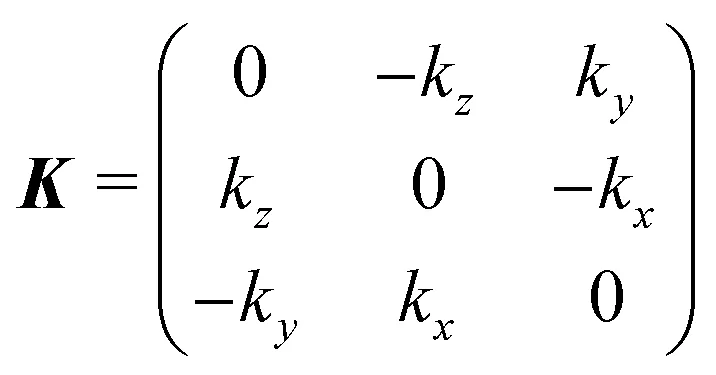
***R***_*ϕ*_ is an in-plane rotation (*xy*) perpendicular to the incident direction, and is given by:8
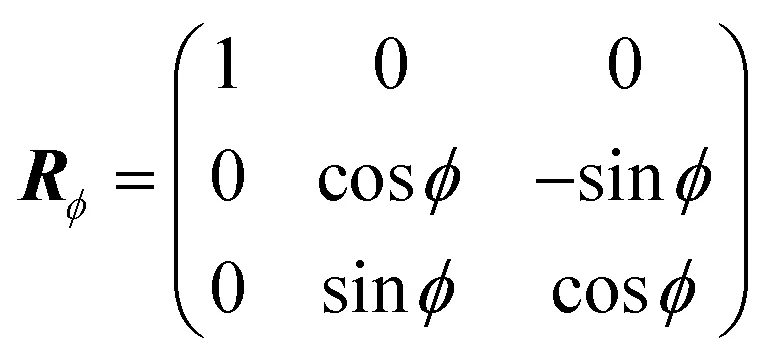
The *I*_Γ*j*_ were used to calculate the differential cross sections (d*σ*_Γ*j*_/d*Ω*) for Stokes scattering with respect to the solid angle *Ω* according to:^47^9
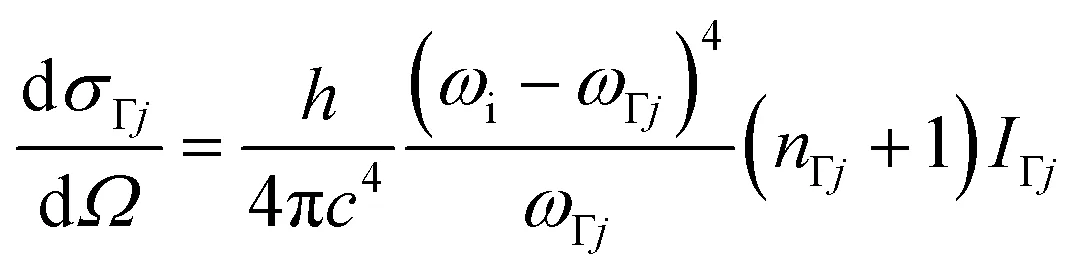
where *h* is the Planck constant, *c* is the speed of light, *ω*_Γ*j*_ is the angular phonon frequency and *ω*_i_ is the angular frequency of the incident light, and *n*_Γ*j*_ is the phonon occupation number calculated at *T* = 300 K.

Polar plots were simulated by calculating and summing the differential cross sections for 532 nm incident light polarised along ***ê***_i_ = *x* and scattered light with parallel ***ê***_s_ = *x* and perpendicular ***ê***_s_ = *y* polarisations over a range of in-plane rotation angles *ϕ*. We note that we assumed the far-from-resonance approximation, *i.e.* we evaluated the ***α***_Γ*j*_ at *ω*_i_ = 0.

### Interfacial angle measurements

Steno's law of constant dihedral angles states that the angle between the crystal facets of the same material is constant regardless of the rate of facet growth (Fig. S3a).^48^ This indicates that facet assignments can be validated by measuring the angle between crystal facets and comparing it to the angle between the corresponding crystallographic planes *e.g.* in the CCDC Mercury software.^49^

High-resolution side-on images of the sample crystals were taken using a Theta Flex Tensiometer with a 2″ CMOS USB 3.0 digital camera and homogenous LED background lighting. These images were then annotated as shown in Fig. S3b to highlight the front edges of the crystal facets and reduce the impact of non-uniform crystal growth on the measured angles. The angle between these edges, *θ*_B_, were measured using the ImageJ software,^50^ and the interfacial angles, *θ*_A_, were determined from:

10 *θ*_A_ (deg) = 180 − *θ*_B_

## Results and discussion

### Powder X-ray diffraction

Whilst the size of the crystals used in this feasibility study is not representative of a typical pharmaceutical material, identification of these large crystal facets acts as a first step to understanding the Raman behaviour of individual facets. In order to identify the large crystal facets, powder X-ray diffraction was performed by approximately aligning the facet with the top of a solid Perspex sample holder in the focal point of the incident X-ray beam. The preferred orientation yielded characteristic Bragg peaks that could be indexed by comparison to predicted patterns obtained from the CrystalDiffract software.^34^ A selection of PXRD patterns with each of the dominant crystal facets A, B and C aligned in the beam are presented in Fig. 4, with complete patterns for all of the facets analysed provided in Fig. S4.

Crystal habit predictions based on the attachment energy model of crystal growth with the Dreiding II forcefield (CCDC: **HXACAN01**)^38^ suggest that PCM-I should crystallize as rounded particles (aspect ratio 1.2–1.8), as illustrated in Fig. 2a. The dominant crystal facet is predicted to be the (001) facet, as indicated by the lowest attachment energy (−40.534 kJ mol^−1^), followed by the (110) (−45.746 kJ mol ^−1^) and (011) facets (−46.649 kJ mol^−1^).

As demonstrated in Fig. 4a, facet A exhibits a strong Bragg peak between 2*θ* = 13–14° and a weaker secondary peak at 2*θ* ≈ 28°. This is an excellent match to the predicted powder pattern for the (001) facet, with the smaller secondary peak characteristic of the (002) Bragg reflection and present in PXRD measurements of facet A for all bar one of the samples. Facets B and C exhibit similar patterns, with a primary Bragg peak between 16.4–17.0° and a smaller signal between 28.5–29.4°. The stronger features between 16.4–17.0° can be assigned as the (011) reflection, and the secondary signal at higher 2*θ* can only reasonably be identified as the (121) reflection, with the predicted (022) signal at 34° not seen potentially due to a significantly lower intensity. The dominant facet A of sample 3 shows a single Bragg reflection at 2*θ* = 18°, which best matches predictions for the (010) facet. However, due to poor crystal quality, it is not possible to make this assignment confidently and we therefore do not include this facet in the ARPRS and interfacial angle analysis data sets. Data for this facet is presented separately in the SI (Fig. S4 and Tables S2 and S9).

**Fig. 3:**
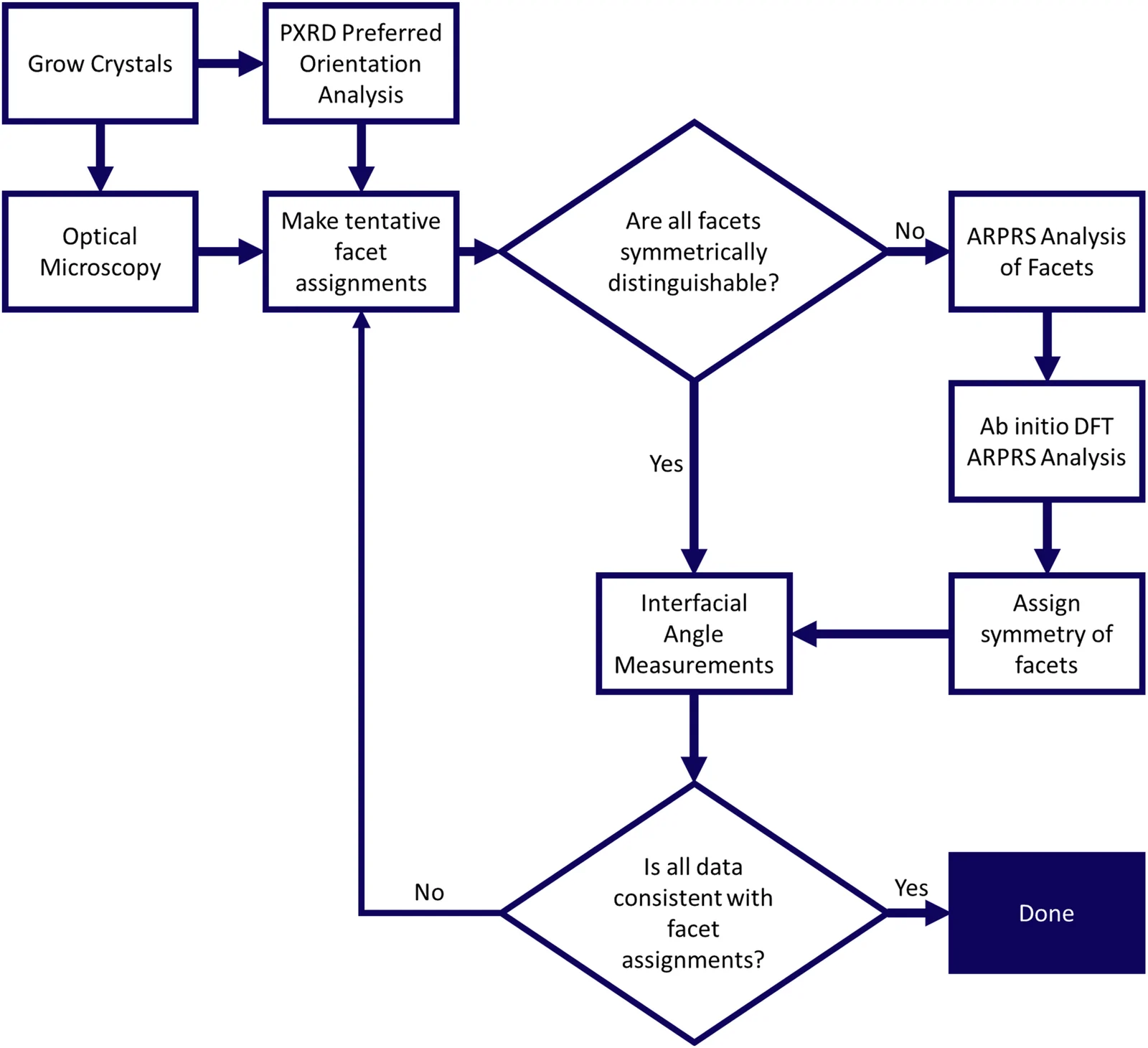
Workflow for facet identification for large single crystals using powder X-ray diffraction and angle resolved polarised Raman spectroscopy measurements.

**Fig. 4:**
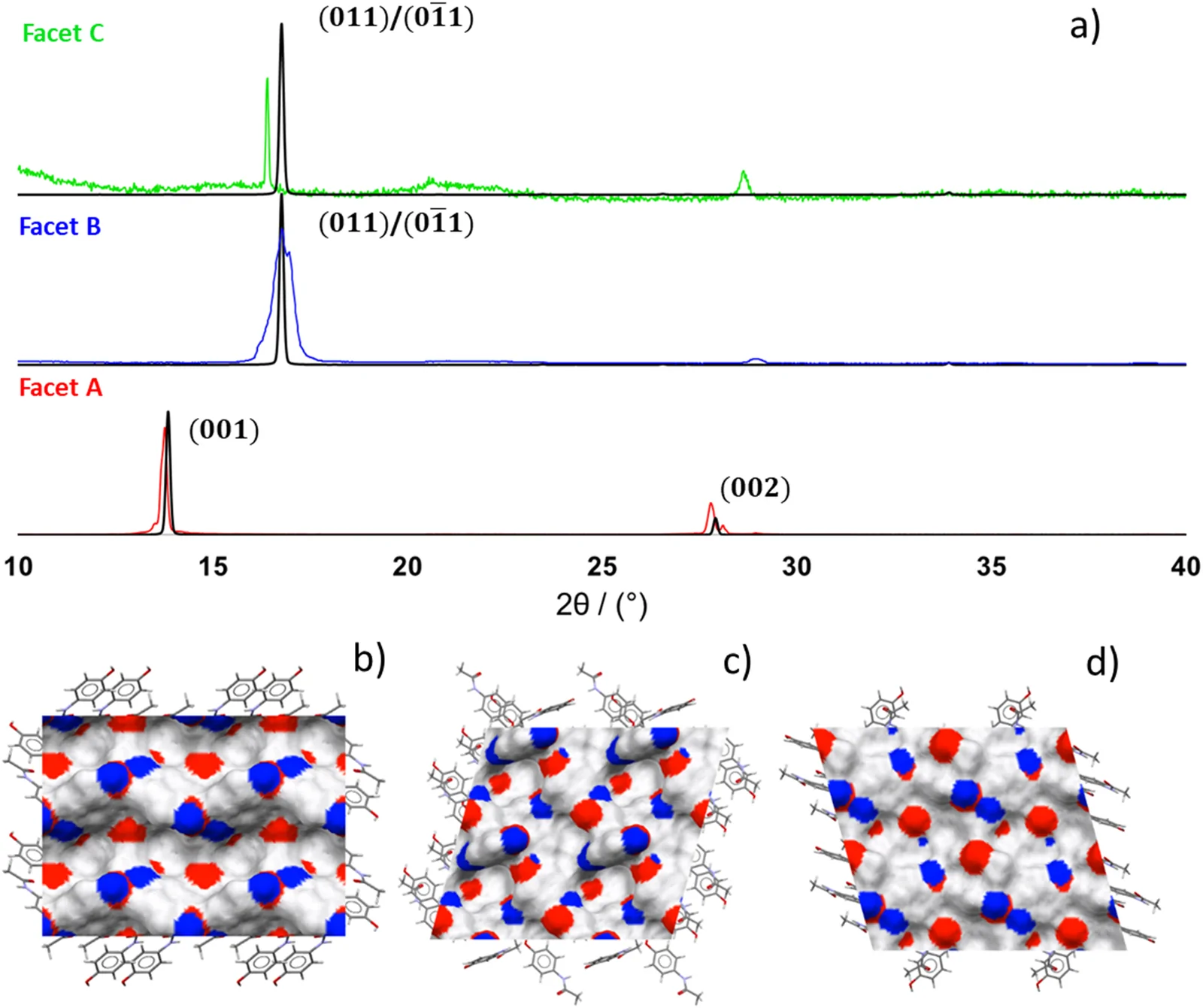
(a) Powder X-ray diffraction patterns of the dominant facets A, B and C in paracetamol form I compared to predicted peak positions for the HXACAN01 structure using the CrystalDiffract^34^ and CCDC Mercury software.^49^ (b)–(d) Surface analysis of the (001) (b), (011) (c) and (01̄1) (d) surfaces using Mercury, showing the positions of H-bond acceptors (red) and donors (blue).

Due to the centrosymmetric *P*2_1_/*a* structure of PCM-I the *d*-spacing of the (011), (01̄1), (011̄) and (01̄1̄) Miller planes are identical and therefore show identical PXRD patterns. However, as shown in Fig. 4b–d these facets are not chemically identical, with the (011) surface exhibiting greater rugosity (1.999 *vs.* 1.342) and hydrogen bond donor and acceptor densities than the (01̄1) surface (0.034 *vs.* 0.027 Å^−2^, 0.041 *vs.* 0.034 Å^−2^). For comparison, the (001) facet demonstrates a rugosity of 1.815, and hydrogen bond donor and acceptor densities of 0.049 and 0.033 Å^−2^, further highlighting the range of chemical behaviour between different facets (these values were estimated using the Mercury software on the surface terminations with the lowest attachment energies). This chemical difference cannot be probed with PXRD alone. With reference to Fig. 3, at this point in the workflow we can identify facet A, and we can identify facets B and C as either the (011) or (01̄1), but we cannot definitively assign B and C to one of these two planes.

### Angle resolved polarised Raman spectroscopy

**Fig. 5:**
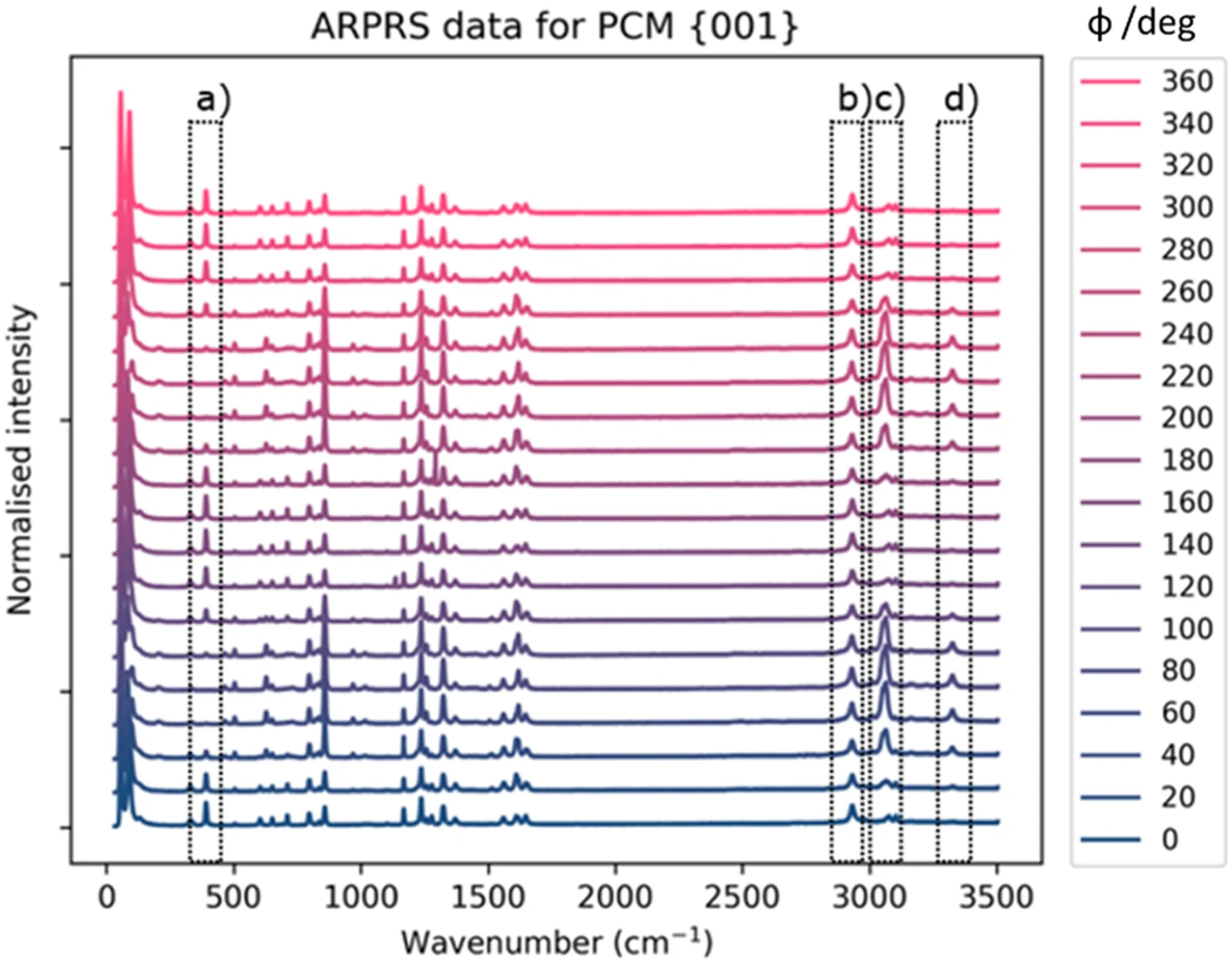
Variation of the normalised Raman intensity measured at the {001} surface as a function of sample orientation angle. The four black boxes show the four bands for which the polarisation behaviour was analyses, *viz.* a) the OH wag, b) the CH_3_ symmetric stretch, c) the aromatic CH stretch, and d) the NH stretch.

Raman spectra of PCM have been reported previously,^51,52^ and suggested assignments of the bands observed in the present measurements based on previous literature^35,36^ and DFT simulations are presented in Table S1. We note that most signals are a combination of multiple functional group vibrations. For ease of analysis and interpretation of the orientations of the vibrational modes, four distinct bands were selected for analysis, namely the OH wag (391 cm^−1^), the CH_3_ symmetric stretch (2931 cm^−1^), the aromatic C–H stretch (3064 cm^−1^) and the NH stretch (3326 cm^−1^). Animations of the vibrational modes are provided as SI. Fig. 5 shows the experimental ARPRS data from the (001) facet, with the spectra offset for clarity, and the measured spectra show good overall agreement with the simulated Raman spectra in Fig. S11. It can be seen that peaks at 391 cm^−1^, 3064 cm^−1^ and 3326 cm^−1^ are polarised, *i.e.,* the intensity of these peaks depends on the crystal orientation with respect to the laser polarisation. In contrast, the CH_3_ symmetric peak at 2931 cm^−1^ exhibits markedly different polarisation behaviour, with similar normalised intensity (NI) across all rotation angles.

**Fig. 6:**
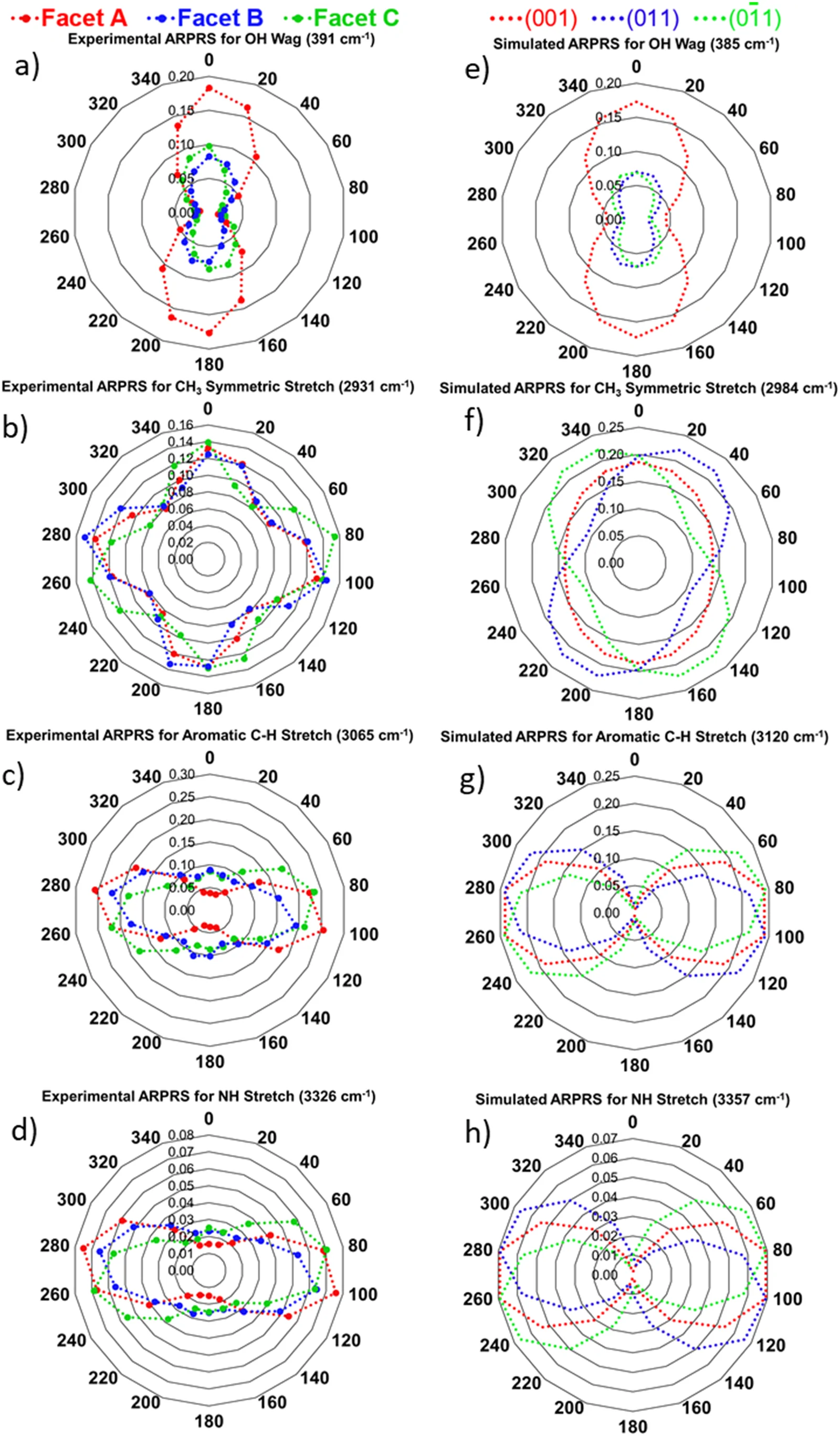
Comparison of the measured ARPRS polar plots for facets A, B and C (a–d) and corresponding computational predictions for the (001), (011) and (01̄1) facets based on calculations on bulk paracetamol form I (e–h).

Fig. 6 compares the measured and predicted changes in band intensity of the of the four selected vibrations as a function of rotation angle at the (001), (011) and (01̄1) surfaces identified in the PXRD measurements and averaged over multiple crystals. A summary of the average NI ranges and depolarisation ratios for each facet, and a comparison to DFT predictions, is provided in Fig. S5 and Table S2. Polar plots for the four selected bands at facets A, B and C of the crystal samples we examined are provided in Fig. S6–S8.

Fig. 6a and b compare the measured and simulated angle dependence of the OH wag intensity for the three different crystal facets. There is a good match between the measured and predicted polarisation behaviour, with the (001) band exhibiting the greatest normalised intensity of the three facets and (011) and (01̄1) bands showing comparable maximum intensities. Notably, the simulations indicate a difference in the angular dependence of the OH wag intensity of the (011) and (01̄1) facets that can also be observed in the experimental data.

Whereas the (001) OH wag is predicted to show a symmetrical polar plot, the (011) data is predicted show significant “skew”, with a higher NI counter-clockwise from the maximum, while the (01̄1) facet is predicted to show the mirror image behaviour with a higher relative NI clockwise from the maximum. These are clearly seen in the experimental data, with the polar plot for facet B showing higher NI at 20° than at 340° (0.08/0.06 NI) and facet C showing the mirror image behaviour. This suggests that facets B and C can be assigned as the (011) and (01̄1) respectively.

For the NH stretch (Fig. 6d and g), all three dominant facets are predicted to show comparable polar plots with maxima at approximately 90° to the OH wag and separated by 180°, which are again a good match to the measured behaviour. Interestingly, this suggests that the polarizability (*i.e.*, Raman tensor) of the NH stretch is largely orthogonal to the OH wag in the *xy* plane in which the measurements are taken. Again, as for the OH wag, the simulations predict a contrast in the polarisation behaviour of the (011) and (01̄1) facets, in the form of a small skew in the positions of the intensity maxima position. The simulations predict that the (001) NH stretch reaches a maximum intensity at sample orientation angles of 90 and 270°, with the (011) maxima at 100 and 280° and the (01̄1) at mirror image offsets of 80 and 260°. While the lower angular resolution of the experimental data makes it impossible to determine the exact position of the maxima of facet A, the polar plots of facets B and C clearly show the contrasting behaviour expected for the (011) and (01̄1), respectively, consistent with the analysis of the OH wag band.

As for the OH wag and NH stretch, the aromatic C–H stretch also produces dumbbell-shaped polar plots with maxima separated by 180° and minima at orthogonal angles (Fig. 6c and g). However, the measurements suggest a larger maximum intensity for the (001) (facet A) than the (011) and (01̄1) (B/C). Nonetheless, the (011) and (01̄1) can once again be distinguished based on the positions of the NI maxima. The simulations suggest the (001) maxima occur at orientation angles of 90 and 270°, whereas the (011) maxima occur at 100 and 280° and the (01̄1) maxima at 80 and 260°. Despite the lower angular resolution, this can also be observed in the experimental polar plots, with a clear skew in the angle dependencies of the measured NI values for the (011) and (01̄1) relative to the (001) consistent with the facet assignments made from the OH wag and NH stretch.

The polar plots for the OH wag, NH stretch and aromatic CH stretch all producing dumbbell-shaped polar plots are sufficiently consistent with the DFT simulations to allow for unambiguous facet identification. However, the CH_3_ symmetric stretch appears to exhibit more complex polarisation behaviour that is not well captured by the simulations (Fig. 6b and f). Whereas the experimental data indicate four roughly equal NI maxima spaced 90° apart, the simulations predict an uneven polar plot with two primary and two weaker secondary NI maxima.

From the simulations the aliphatic CH_3_ stretch is composed of five overlapping bands, which complicates the interpretation of the experimental polar plot as this only considers the intensity change at a single wavenumber. Potential discrepancies between the experimental and predicted relative intensity of the overlapping bands could also play a role.

Despite this discrepancy, as observed with the other modes the measurements and predictions both show a characteristic skew that can be used distinguish the (011) and (01̄1). The simulations predict that the (011) and (01̄1) NI maxima occur at sample orientations of around 20 and 340°, respectively, which are mirror images about the (001) maximum at 0°. While the experimental data indicate maxima at 0° for all three facets, the measurements for facets B and C still show notable differences in NI at 20 and 340° (0.12/0.09 for facet B and 0.09/0.12 for facet C) that can be used to assign them to the (011) and (01̄1) facets.

**Fig. 7:**
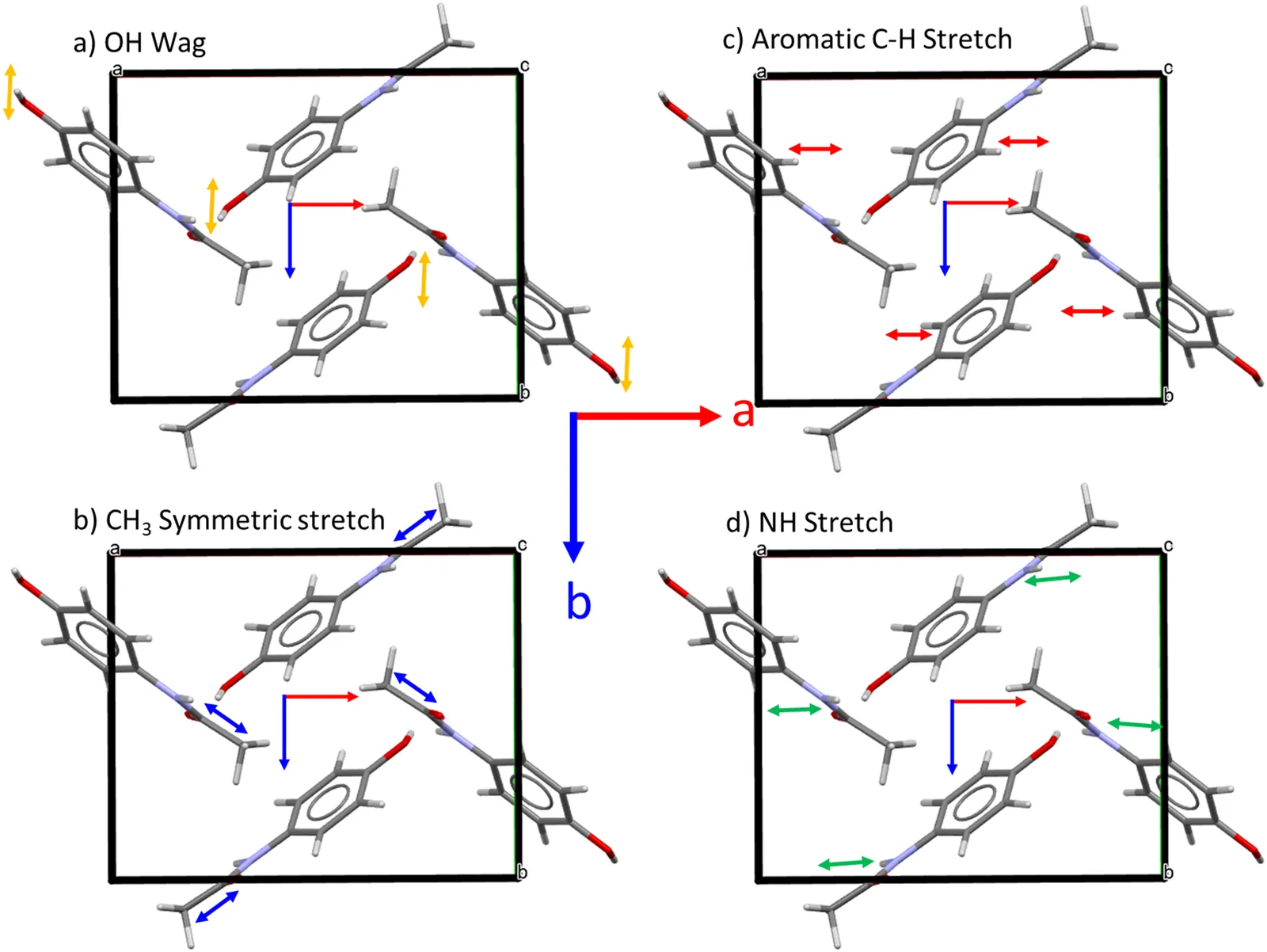
Relative orientation of the polarisability changes (Raman tensors) for the OH wag (a), CH_3_ symmetric stretch (b), aromatic C–H stretch (c) and NH stretch (d) vibrations viewed perpendicular to the (001) facet plane.

The relative orientations of the polar plots in Fig. 6 indicate the relative orientations of the Raman tensors of the four vibrations (Fig. 7, *c.f.*Fig. 1). As noted above from the polar plots, the Raman tensors of the OH wag are orthogonal to both the NH stretch and the aromatic C–H stretch. Fig. 7b shows an indicative change in polarizability for just the CH_3_ breathing mode. Unlike the other three vibrations (Fig. 7a, b and d), where all four molecules in the unit cell show the same net stretching behaviour, the CH_3_ symmetric stretch involves two pairs of orthogonal stretches that would result in similar but orthogonal changes in polarizability at 90°. This accounts for why this band shows four intensity maxima, as reflected in the experimental data.

This observed angular dependence of the OH wag, NH stretch and aromatic C–H stretch intensities is comparable to previous work on l-alanine by Nemtsov *et al.*^26^ This study found that the net polarisation of a given vibrational mode determines the relationship between the observed Raman intensity and the orientation angle relative to the polarisation of the incident laser.^26^ In this work, the shear vibrational mode (48 cm^−1^) at the (101) and (002) facets of l-alanine showed comparable polarisation behaviour due to a similar net stretching direction of the hydrogen bonds, whereas the (011) showed markedly different behaviour due to roughly equal populations of orthogonal hydrogen bond stretches. The observed ARPRS behaviour is also supported by measurements from Xue-Lu *et al.* showing that the G band at the edge plane of highly ordered pyrolytic graphite has anisotropic polarisability and reaches an intensity maximum when the polarisation directions of the incident and scattered light are parallel to the basal plane.^24^ However, in contrast to the structurally simple 2D material in ref. 24, analysis of three-dimensional organic crystal system is far more challenge due to the fact that most spectral regions contain multiple vibrational modes that typically result in overlapping features.^33^

We also note that the DFT simulations predict that the relative intensities of the NH and aromatic C–H stretches fall to zero at some sample rotation angles, but this is not observed experimentally. Also, the measurements for the NH and aromatic CH stretch show that the NI values for the (011) and (01̄1) facets reach local intensity maxima orthogonal to the global maxima, whereas those for the (001) facet reach minima, and this is also not predicted by the simulations. Finally, the measurements indicate a greater NI maximum for the OH wag at the (011) and (01̄1) facet, whereas the simulations predict identical intensity maxima. While these discrepancies could be due in part to experimental issues such as the lower angular resolution and the difficulty of accurately extracting band intensities from the measured spectra, it is also possible that the measurements show differences due to scattering from the sample surface. As demonstrated in Fig. 4, the (011) and (01̄1) surfaces potentially exhibit significant differences in surface structure, but this is not captured by the simulations of the bulk material. Other differences between the measurements and simulations that could also contribute to the observed differences include, for example, the influence of the chosen laser power on the band intensities, and further work is required to investigate these in more detail.

### Interfacial angle measurements

To validate the facet assignments from PXRD and ARPRS, we measured the interfacial angles between facets A, B and C and compared them to the expected angle between the corresponding crystallographic planes (Table 1). The predicted angle of 34.2° between the (001) and (011)/(01̄1) is largely comparable with the average measured angles of 34.5° and 35.5° between the dominant facet A and angled facets B and C respectively. These measurements therefore support the facet assignments from PXRD and ARPRS analysis. The small differences in the measured interfacial angles can be explained by step growth of the crystal facets complicating the determination of the position of the crystal facet edges.^53^

**Table 1:** Comparison of the measured interfacial angles between the major facet A and angled facets B and C with the predicted angles between the assigned crystallographic planes obtained for the reported HXACAN01 structure using the CCDC Mercury software^49^

Facet	Assignment	Measured angle (°)	Predicted angle (°)
A–B	**(001)**–**(011)**	34.5 ± 3.8	34.2
A–C	**(001)**–**(01̄1)**	35.5 ± 4.1	34.2

As noted above, we excluded the dominant facet A of sample 3 from our analysis of the (001), but this sample demonstrated interfacial angles of 150.82° and 144.89° with facets B and C respectively, which are similar to the angles observed for the other samples. However, the poor overall crystal quality makes us less confident in these measurements. A table listing the measured interfacial angles of all the samples is provided in Table S3.

## Conclusions

In this proof-of-concept work we have presented a workflow for the identification of large single crystal facets utilising a combination of powder X-ray diffraction, angle resolved polarised Raman spectroscopy, and DFT simulations, and demonstrated its application to the model system paracetamol form I. While PXRD measurements of aligned single crystals yield Bragg reflections characteristic of particular facets, crystal symmetry may prevent unambiguous facet assignments. In the present case, the centrosymmetric *P*2_1_/*a* crystal structure of PCM-I results in the (011) and (01̄1) facets being indistinguishable by PXRD, with both presenting comparable PXRD patterns. By comparing measured ARPRS spectra to simulations based on bulk PCM-I, these symmetry related facets can be differentiated based on the relative positions of Raman intensity maxima as a function of sample rotation. ARPRS measurements at the (011) and (01̄1) facets generally forming mirror images, and the resulting “skew” in the polar plots relative to the (001) allow the facets to be assigned with reference to the simulations. Despite some discrepancies between the simulations and measurements, unambiguous facet assignment was possible, and the assignments were validated by comparing measurements of the interfacial angles between the facets with the predicted angles between the assigned crystallographic planes.

Whilst we recognise the limitations of this technique, this paper acts as a proof of concept for this approach. Our vision is that this can be developed further to overcome the requirement of large crystals by applying the same workflow to a batch of API powder with crystals comparable in size to material from a typical pharmaceutical manufacturing process. While we have tested our workflow for PCM, it should be fully general and can be applied to other materials to provide further validation. Further work could also attempt to determine absolute crystal orientation from ARPRS data, as has been done for 2D materials. Ultimately, it is also our ambition to develop our workflow into a robust method for studying other organic surfaces and their interfaces with solution and/or other materials, where the non-destructive nature and chemical specificity of Raman measurements could provide unique advantages compared to other, more established techniques.

## Author contributions

DC was responsible for data curation, data interpretation, formal analysis, investigation, methodology development, visualisation and validation of the experimental work, as well as writing the original draft of the manuscript. JMS was responsible for software development, data curation, data interpretation, formal analysis and computational methodology development, as well as reviewing and editing of the drafts. ARP and HB were responsible for conceptualisation of the work, funding acquisition, providing resources, supervision, data interpretation and reviewing and editing of the draft. SLMS and MJ contributed to supervision, data interpretation and reviewing and editing of the draft.

## Conflicts of interest

There are no conflicts to declare.

## Supplementary Material

CE-028-D5CE00672D-s001

## Data Availability

The Phonopy-Spectroscopy code used to perform the Raman simulations is open source and available to download from https://github.com/skelton-group/Phonopy-Spectroscopy. Raw data for this article, including powder X-ray diffraction data, angle resolved polarised Raman spectra, interfacial angle measurements, and simulation data including the optimised crystal structure, phonon calculations and Raman simulations, are available from Mendeley Data at DOI: https://doi.org/10.17632/rb7w2ws48r. Supplementary information (SI) for this article is available as a PDF file. This includes additional data to support the discussion in the text, *viz.*: bright- and dark-field micrographs of the five crystals; illustration of the setup used for the PXRD measurements; illustration of the technique used to measure interfacial angles; additional data from the PXRD and Raman measurements; a simulated phonon spectrum of bulk paracetamol and simulated ARPRS spectra for the three crystal surfaces examined in this study; detailed benchmarking of the impact of ***k***-point sampling and computational approach on the simulated Raman spectra; and additional discussion of how surface terminations impact the Raman spectra. See DOI: https://doi.org/10.1039/d5ce00672d.
