# Supplementary material for: Determination of the crystallographic orientation of organic crystal facets with angle-resolved polarised Raman spectroscopy
Source: CrystEngComm. 2025 Dec 3;28(4):836–48. doi: 10.1039/d5ce00672d (PMC13584181; doi:10.1039/d5ce00672d)
Supplement: Supplementary file 1 [file CE-028-D5CE00672D-s001.pdf]

Supplementary Information (SI) for CrystEngComm.
This journal is © The Royal Society of Chemistry 2025

Determination of the crystallographic orientation of organic crystal facets with angle-resolved polarised Raman spectroscopy: Supporting Information

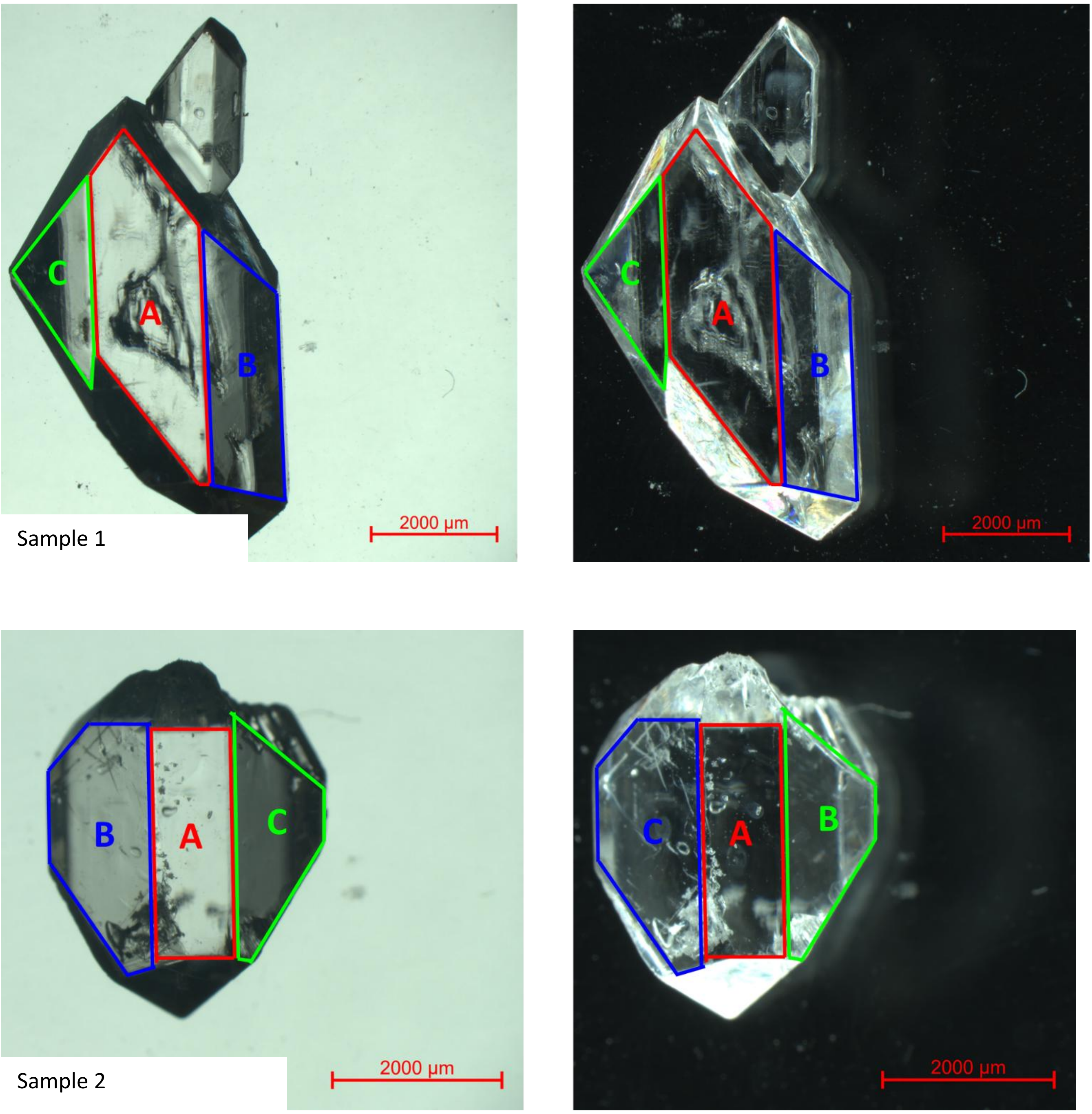

Figure S1. Bright and dark field microscope images of the five paracetamol crystals examined in this work.

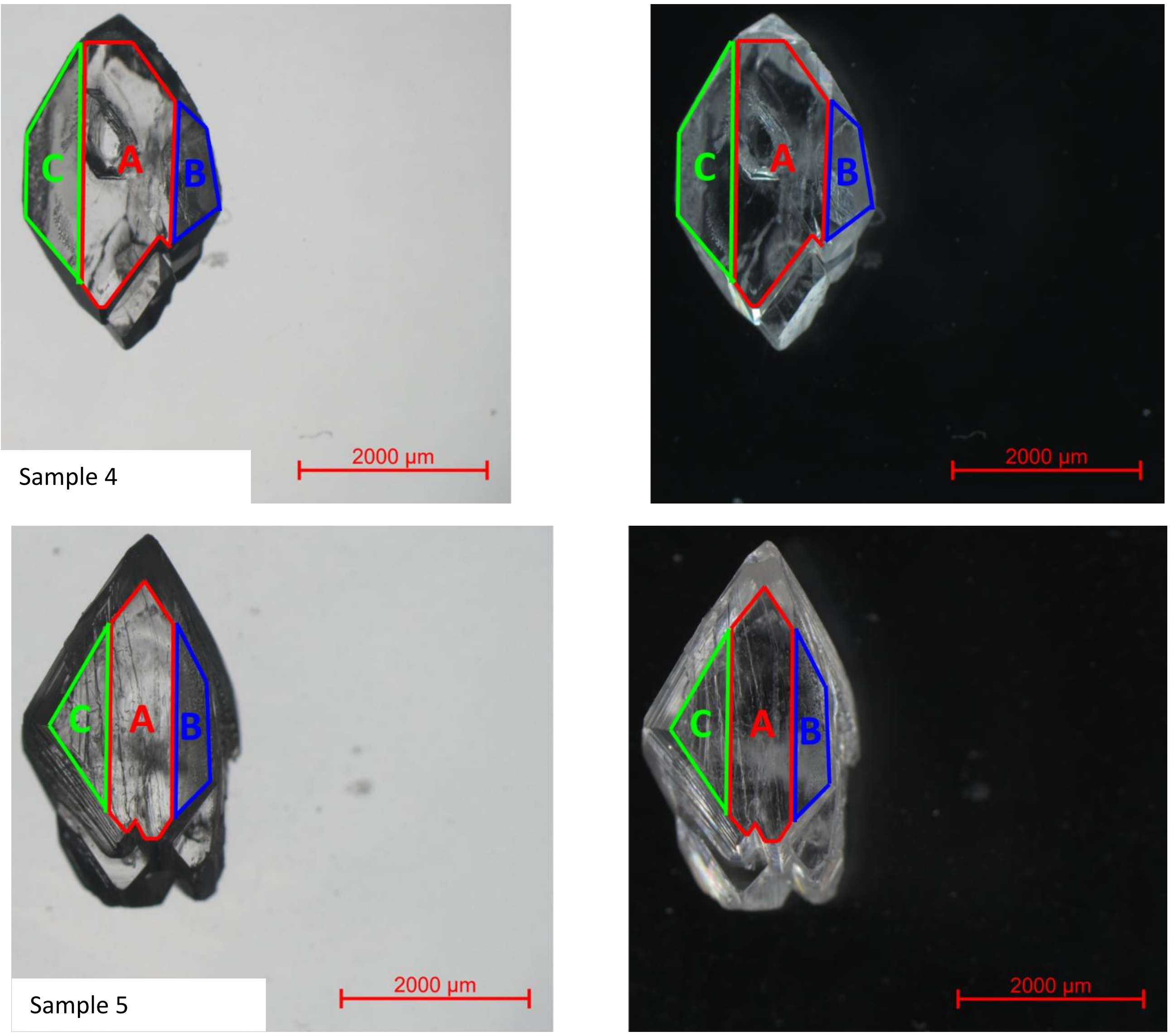

Figure S2 (continued). Bright and dark field microscope images of the five paracetamol crystals examined in this work.

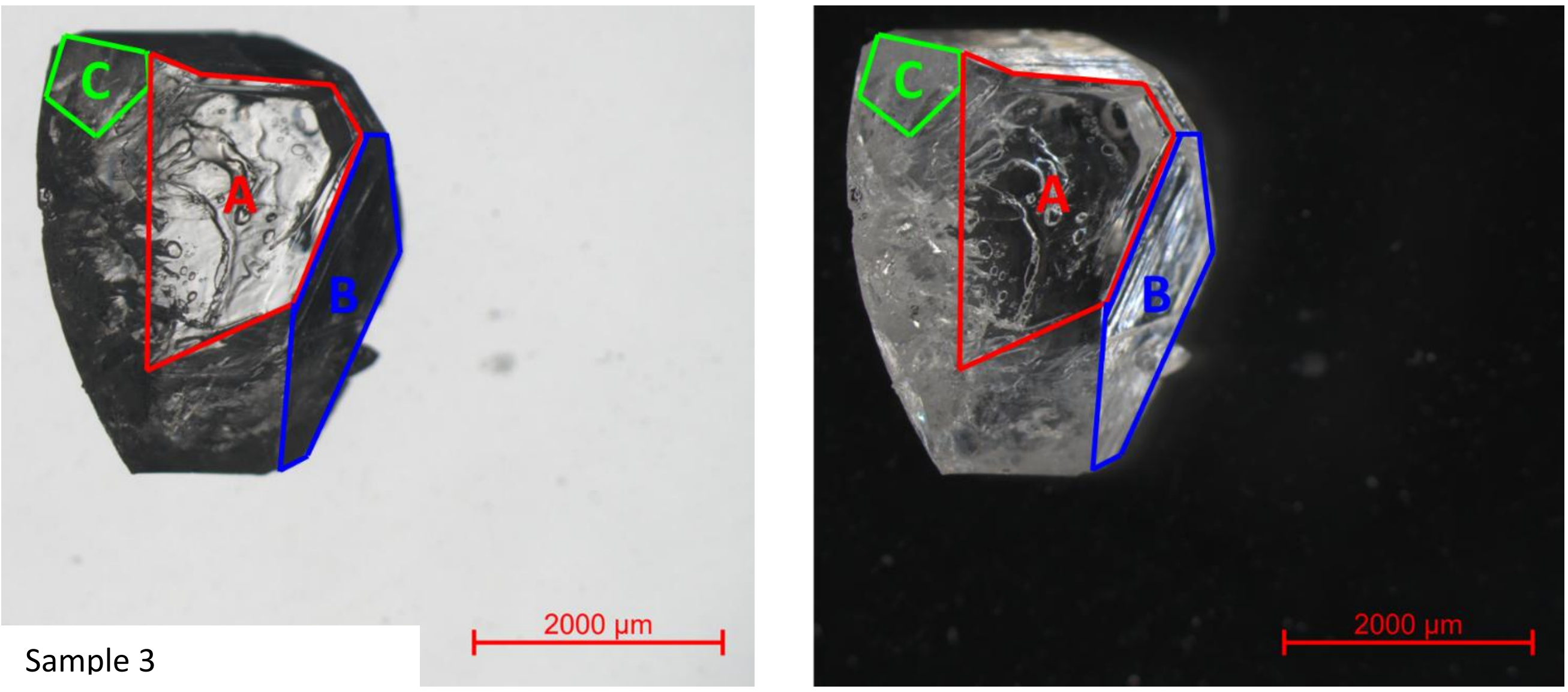

Figure S3 (continued). Bright and dark field microscope images of the five paracetamol crystals examined in this work.

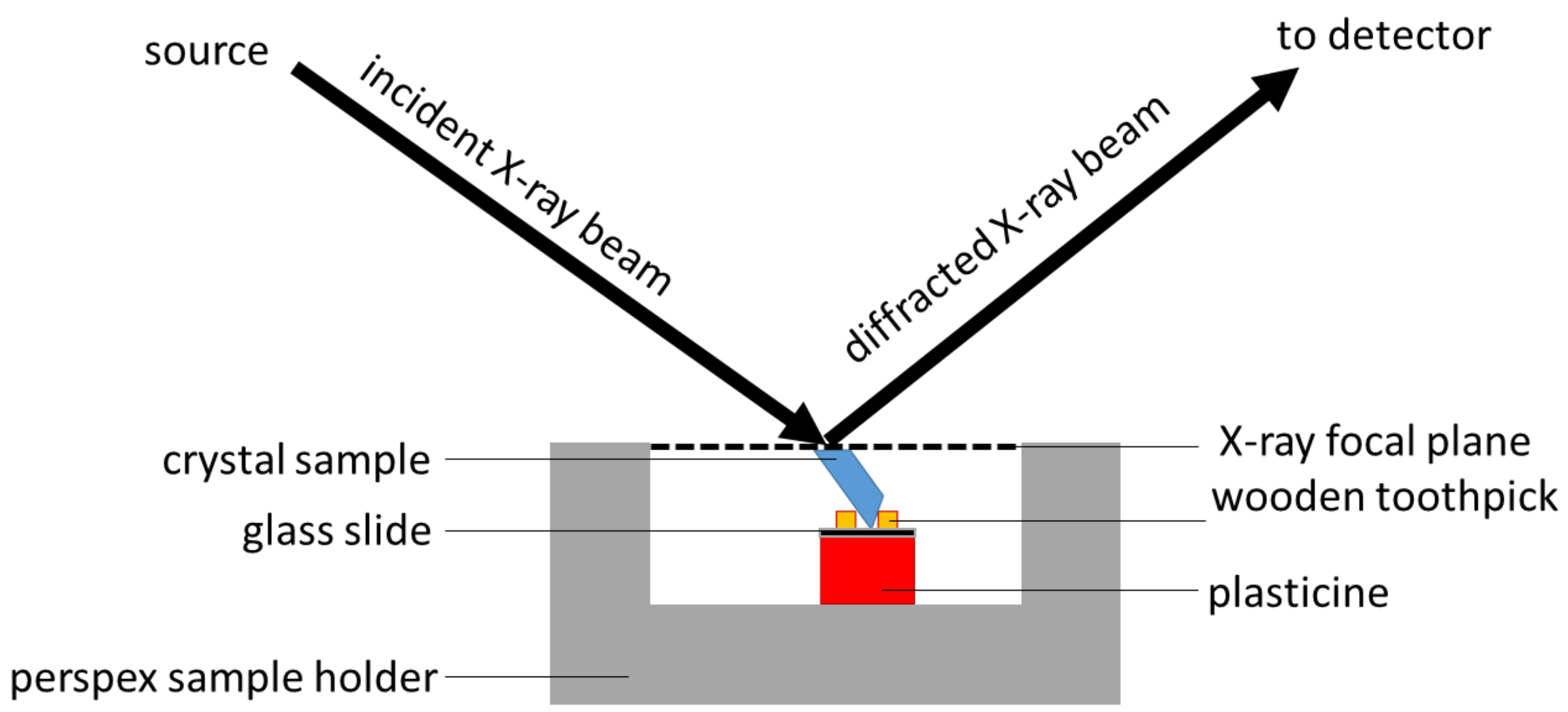

Figure S4. Diagram of the powder X-ray diffraction (PXRD) measurement setup with bespoke wooden frame used in this work, showing facets B and C being accessed by tilting the crystal.

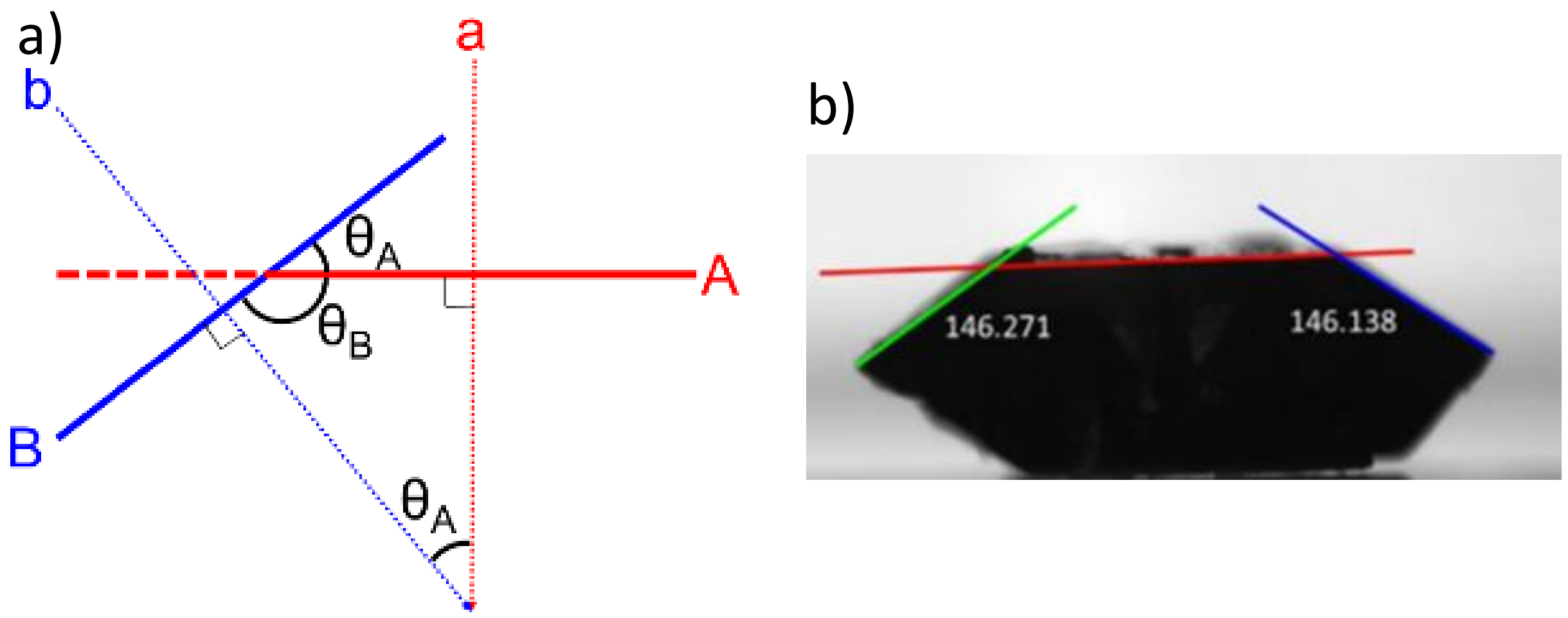

Figure S3. Illustration of Steno's law (a) and annotated tensiometer image showing how interfacial angle measurements were made (b).

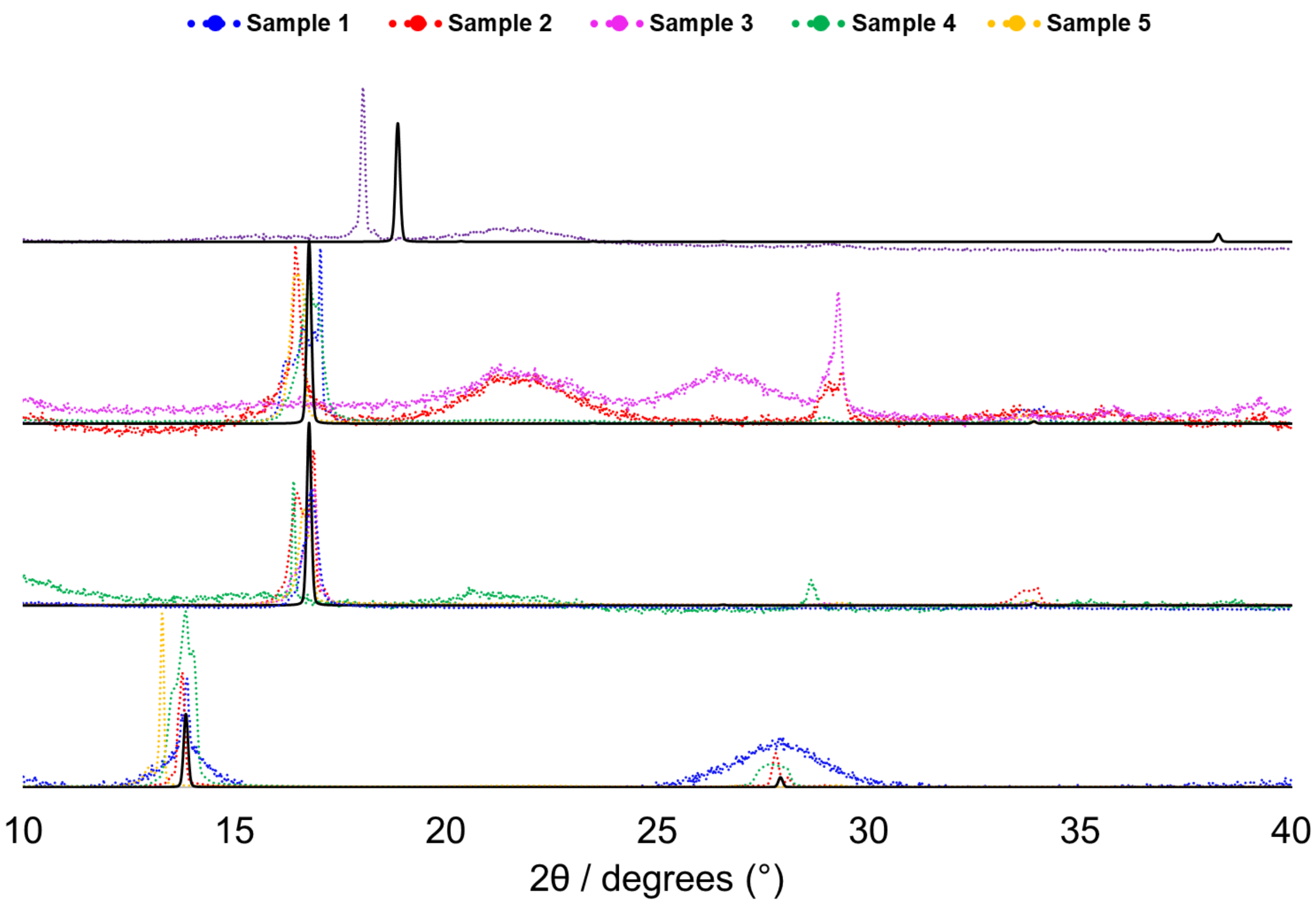

Figure S4. Powder X-ray diffraction (PXRD) patterns of all samples after shifting.

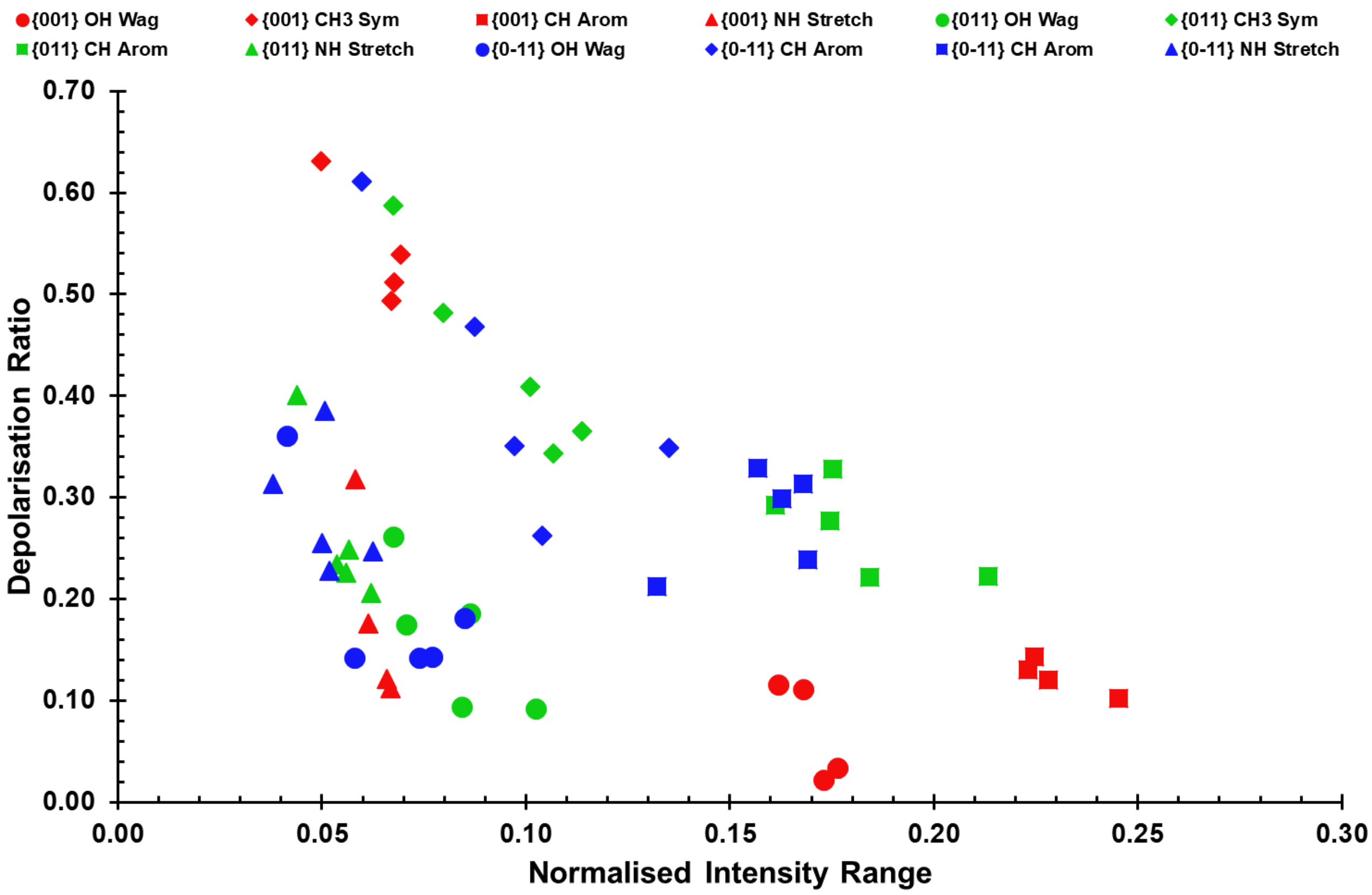

Figure S5. Comparison of the depolarisation ratios and normalised intensity ranges measured from angle-resolved polarised Raman spectra.

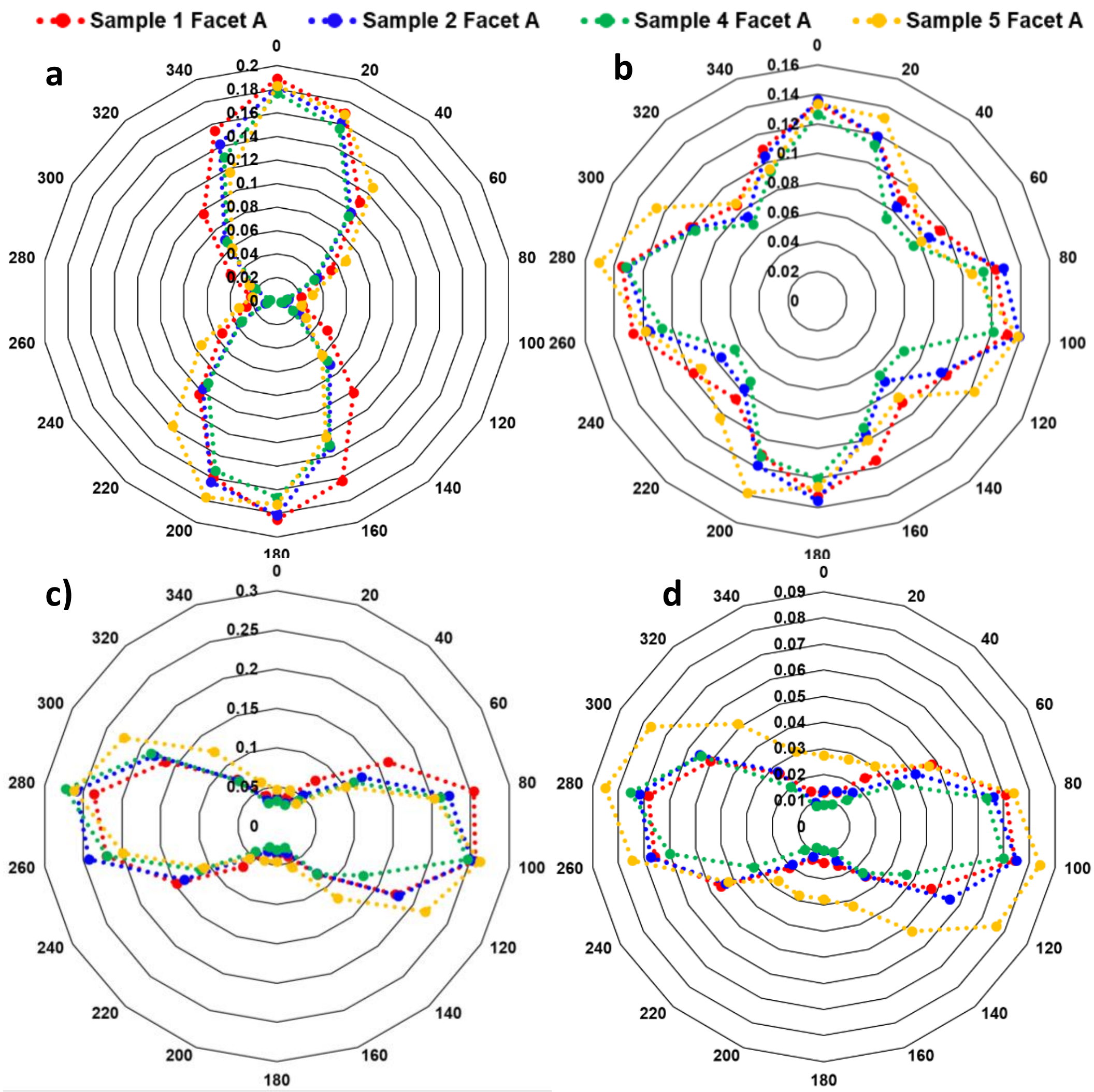

Figure S6. Angle-resolved polarised Raman spectra measurements of the (**001**) experimental facets: (a) OH Wag, (b) CH_3 symmetric stretch, (c) CH Aromatic stretch, and (d) NH stretch.

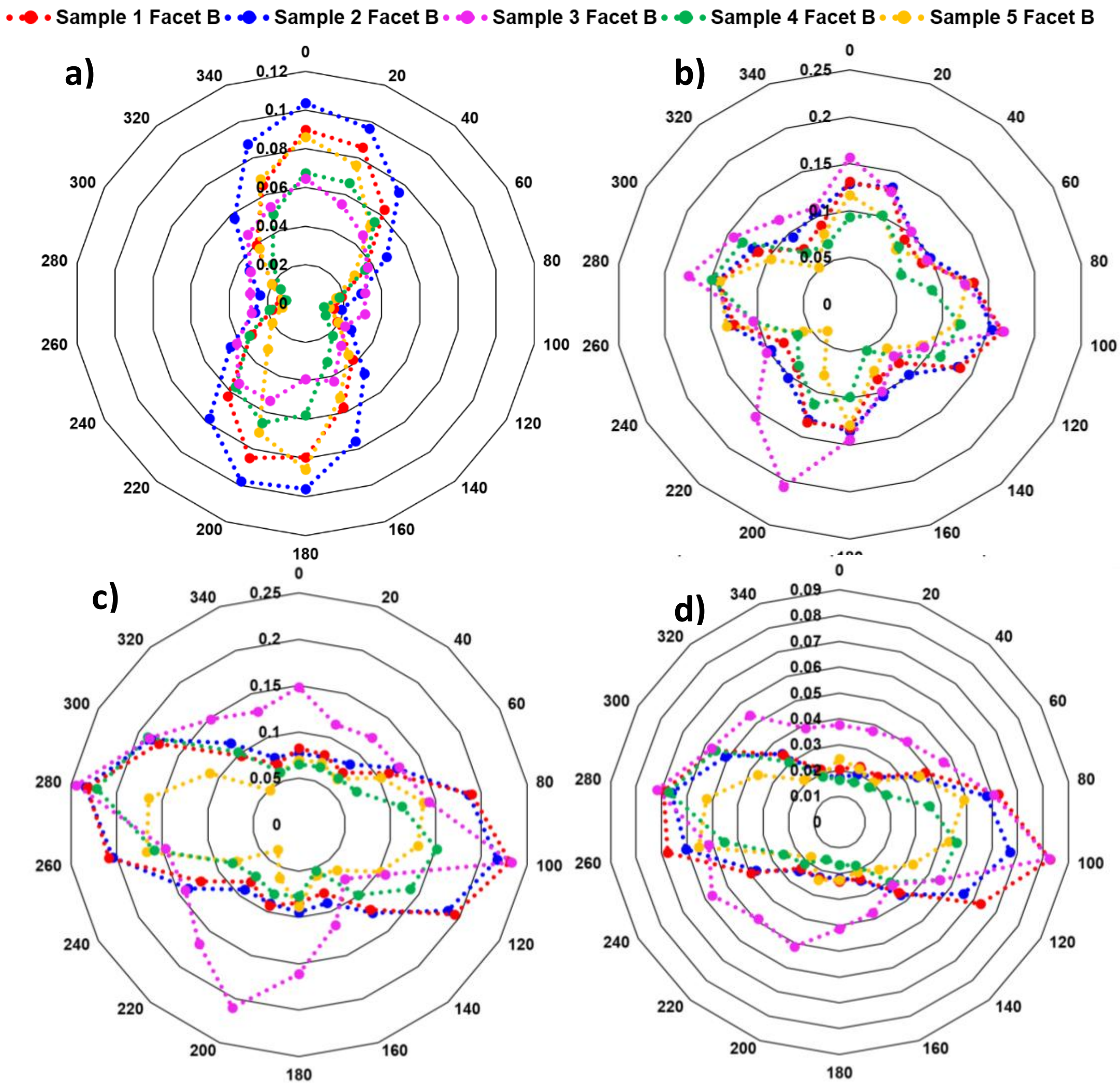

Figure S7. Angle-resolved polarised Raman spectra measurements of the (**011**) experimental facets: (a) OH Wag, (b) CH_3 symmetric stretch, (c) CH Aromatic stretch, and (d) NH stretch.

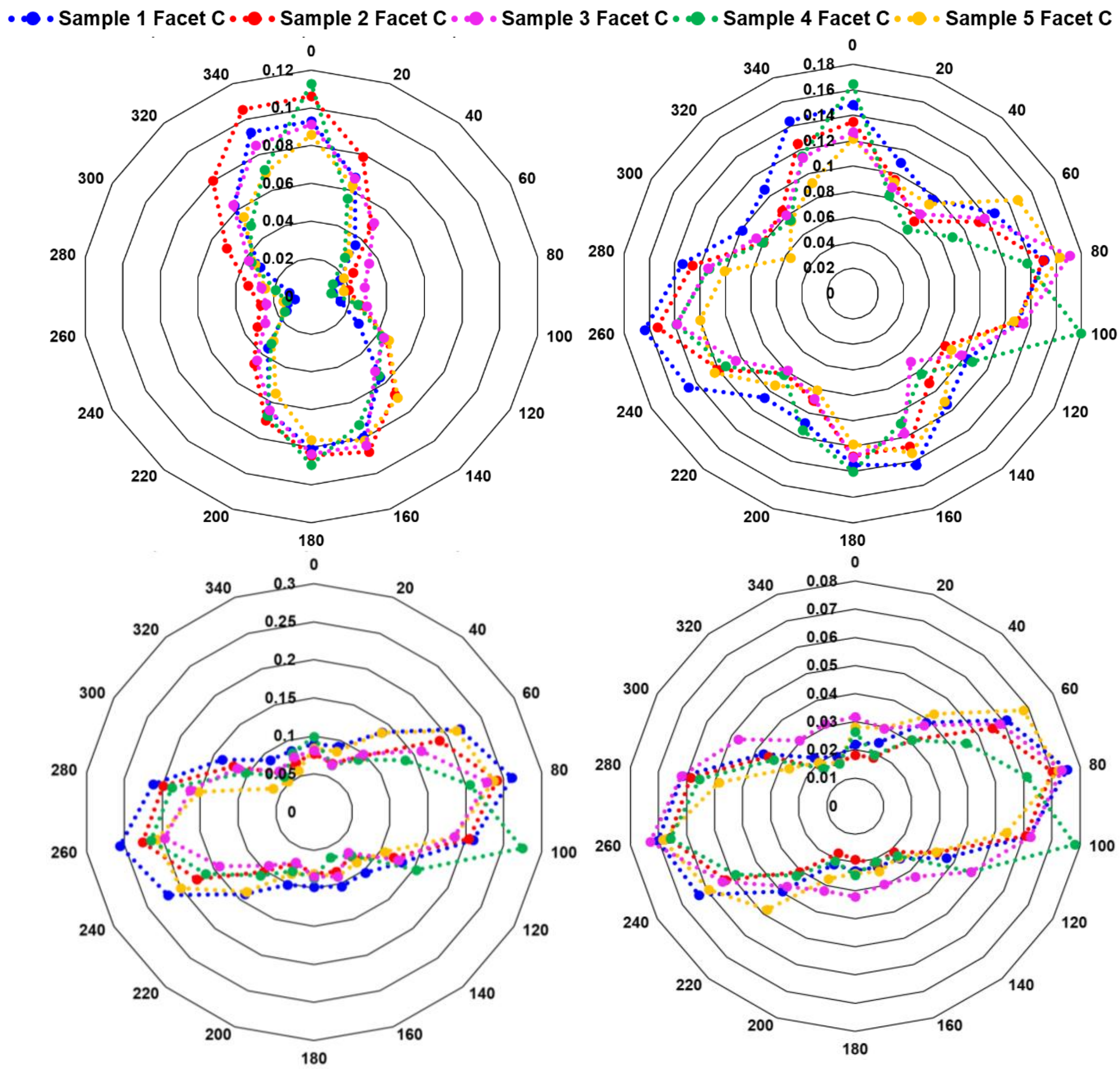

Figure S8. Angle-resolved polarised Raman spectra measurements of the $(\mathbf{0\bar{1}1})$experimental facets: (a) OH Wag, (b) CH_3 symmetric stretch, (c) CH Aromatic stretch, and (d) NH stretch.

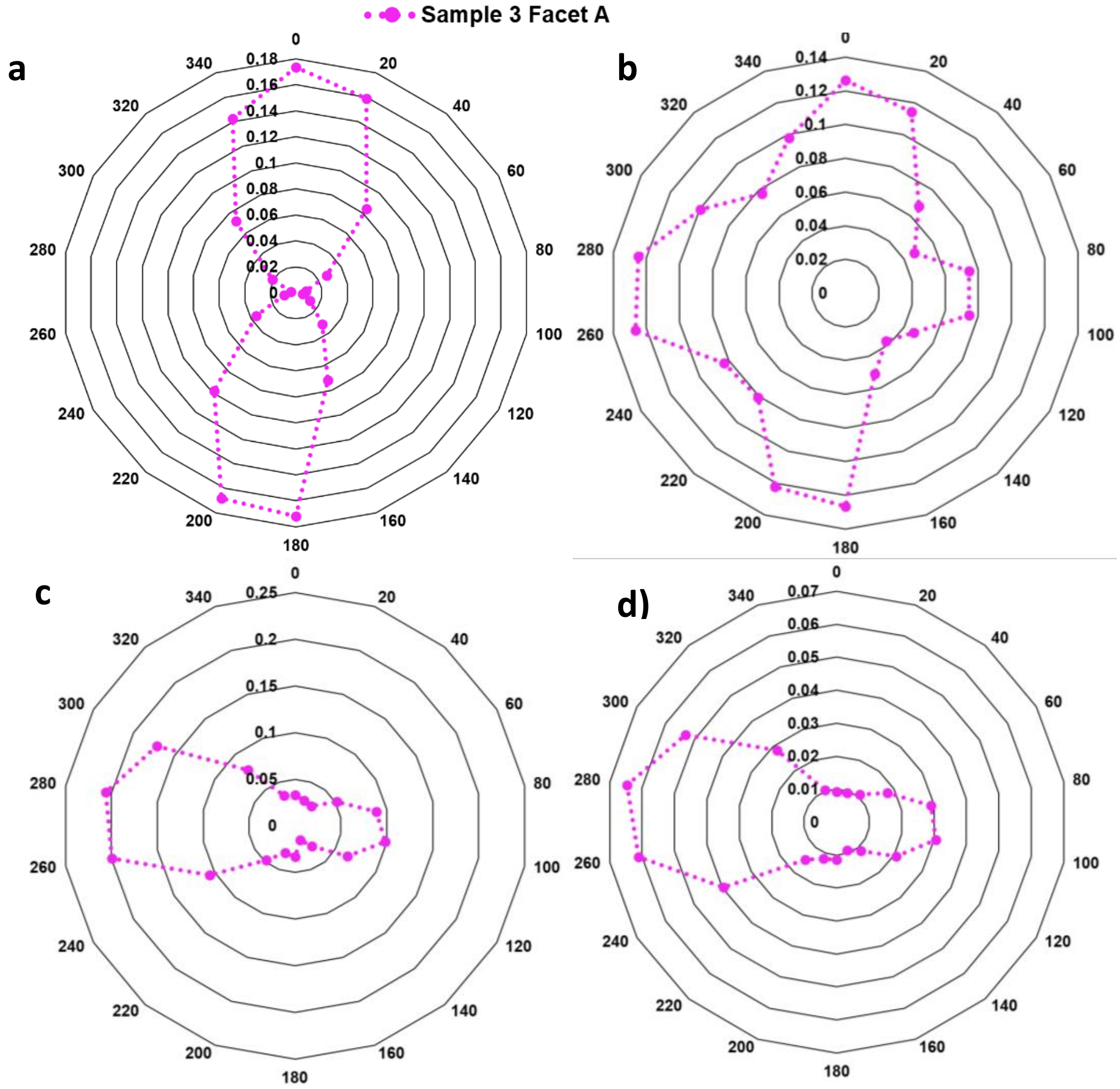

Figure S9. Angle-resolved polarised Raman spectra measurements of Sample 3 experimental Facet A: (a) OH Wag, (b) CH_3 symmetric stretch, (c) CH Aromatic stretch, and (d) NH stretch.

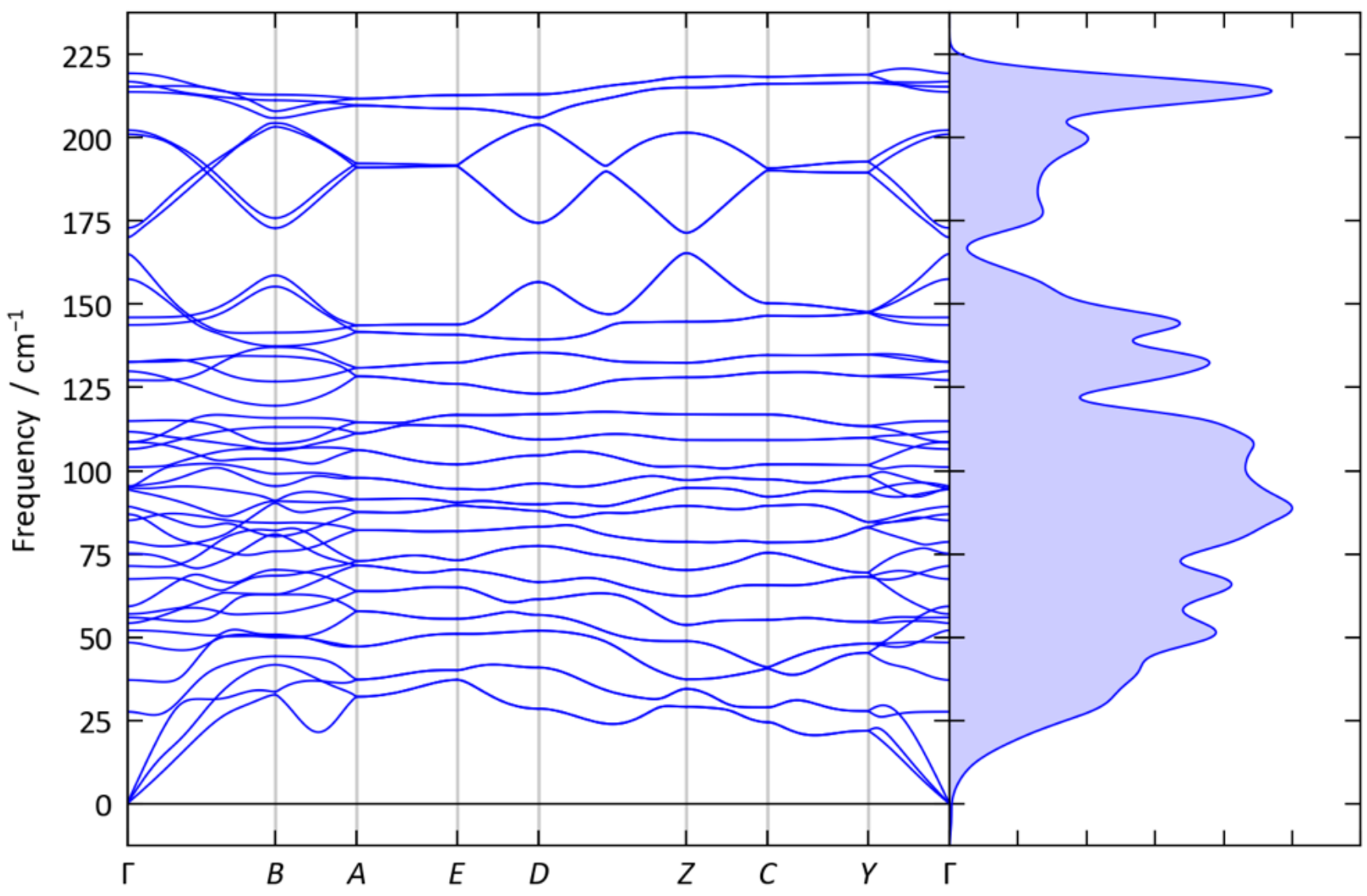

Figure S10. Simulated low-frequency phonon dispersion and density of states (DoS) of paracetamol Form-I.

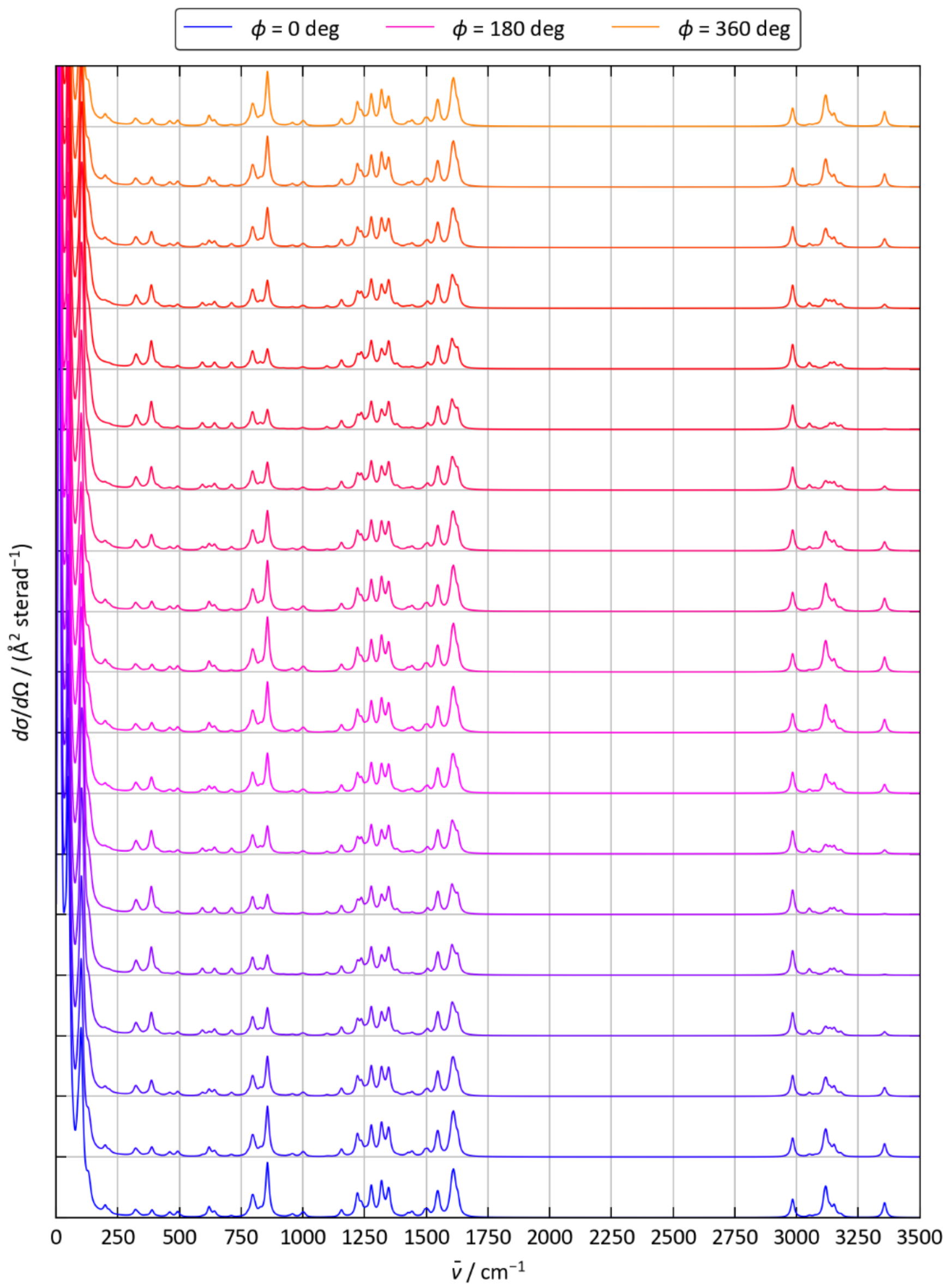

Figure S11. Simulated Raman spectra offset by sample orientation angle of paracetamol Form-I (001) facet. The spectra are colour coded by angle from blue (ϕ = 0 °) to orange (ϕ = 360 °).

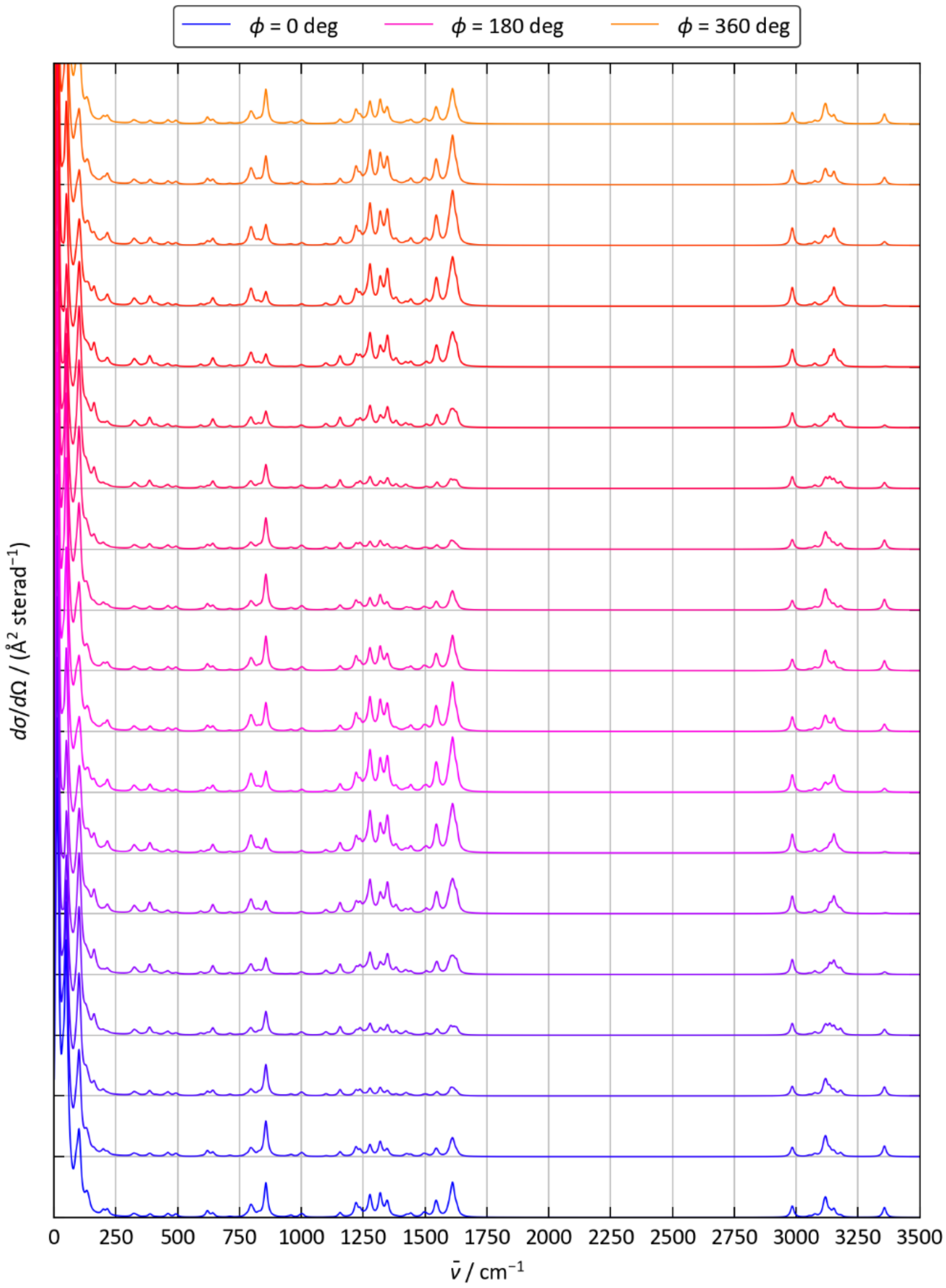

Figure S12. Simulated Raman spectra offset by sample orientation angle of paracetamol Form-I (011) facet. The spectra are colour coded by angle from blue (ϕ = 0 °) to orange (ϕ = 360 °).

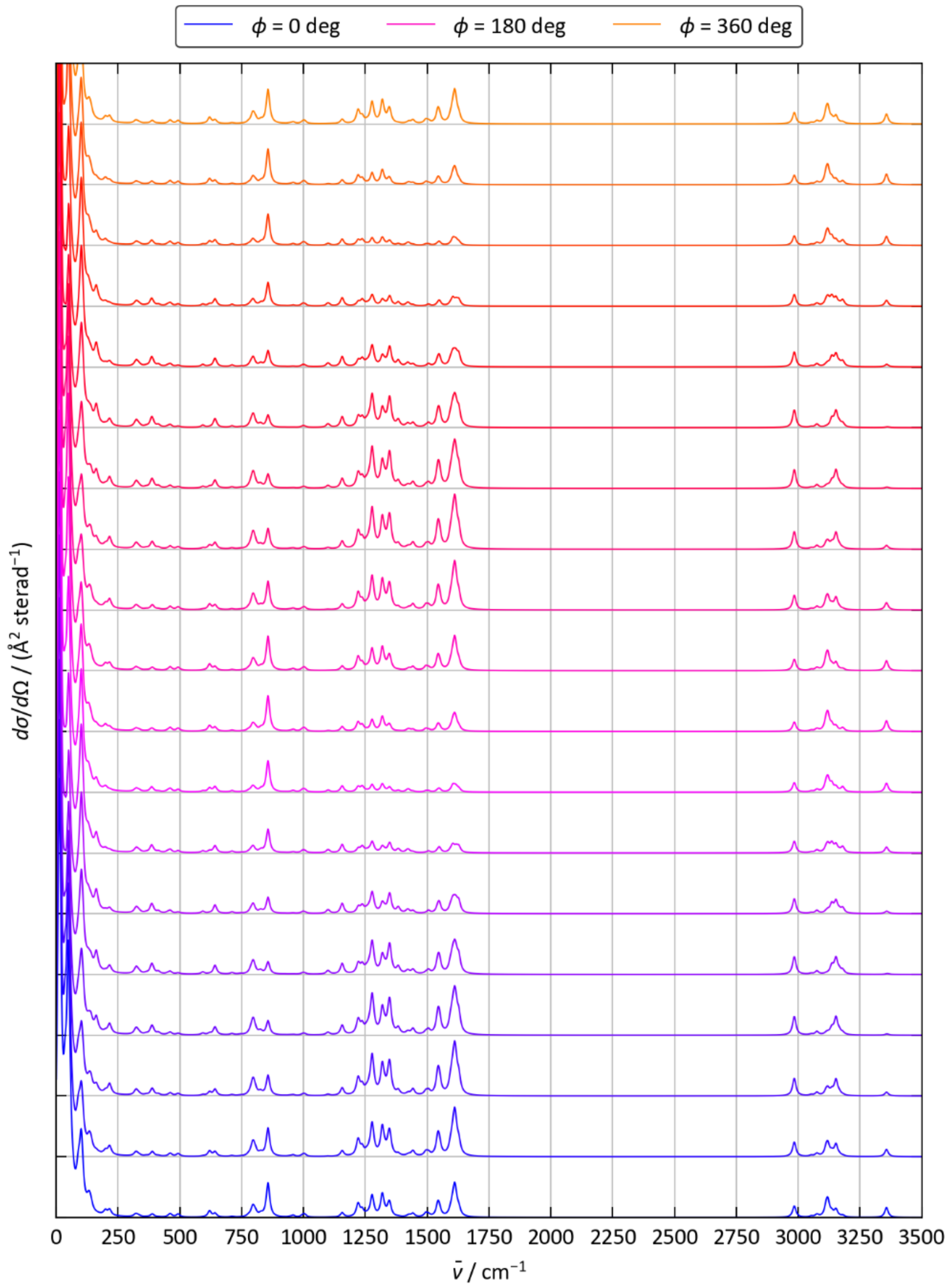

Figure S13. Simulated Raman spectra offset by sample orientation angle of paracetamol Form-I $(0\bar{1}1)$ facet. The spectra are colour coded by angle from blue (ϕ = 0 °) to orange (ϕ = 360 °).

Table S1. Assignment of the vibrational features of paracetamol Form I. ‡ E. B. Burgina, V. P. Baltakhinov, E.V. Boldyreva, T. P. Shakhtschneider, *Journal of Structural Chemistry*, **2004**, **45**, 64-73.

**This work**			**Previous work ‡**		
**Theoretical wavenumber (cm^{-1})**	**Experimental wavenumber (cm^{-1})**	**Assignment**	**Theoretical wavenumber (cm^{-1})**	**Experimental wavenumber (cm^{-1})**	**Assignment**
391	389	OH wag	390	392	ϒ(Ph-Amide)
419	410	δ (Ph-twist)	415	413	δ (Ph-twist)
505	503	ϒ(Ph-Amide)	490	504	ϒ(Ph-Amide)
582	603	Molecule breathing	Not assigned	Not observed	Not assigned
631	626	χPhH + χ amide	621	624	χPhH + χ amide
639	650	δPh +δ amide	645	650	δPh +δ amide
702	710	χCNH + χPhH	699	715, 668	χCNH + χPhH
762	731	δPh +δ amide	765	730	χPhH
781*	795	δPh	799	810, 795	δPh +δ amide
816	830	χPhH	Not assigned	Not observed	Not assigned
819*	834	δPh +δ amide	830	837	χPhH
988*	967	δPhH + δCH_3	969	968	δPhH + vCN
998	1004	δPhH + vCN	Not assigned	Not observed	Not assigned
1013	1017	δPhH + δCH_3	Not assigned	Not observed	Not assigned
1092*	1100	δPhH + vCOH	1095	1107	δPhH + δCH_3
1160	1167	δPhH + vCOH	1171	1172	δPhH + vCOH
1248	1235	δPhH + vCC	1259	1227	δPhH + vCC
1294	1255	δPhH + vCN	1291	1260	δPhH + vCN
1298	1278	δPhH + vCN	1291	1260	δPhH + vCN
1346	1323	δCH_3	1355	1327	δCH_3
1390	1368	δCH_3 + vPh	Not assigned	Not observed	Not assigned
1424	1436	δCH_3	Not assigned	Not observed	Not assigned
1437	1442	δCH_3 + vPh	1466	1442	δCH_3 + vPh
1498	1515	δCNH + vPh	1529	1516, 1506	δCNH + vPh
1583	1561	δCNH + vPh	1559	1565	δCNH + vPh
1607	1609	vPh	1624	1610	vPh
1679	1645	vC=O + δCNH	1683	1653	vC=O + δCNH
2984	2930	vCH_3 sym	3000	2926	vCH_3
3096	3064	vPhH	3093	3032	vPhH
3118	Not observed	vCH_3 asym	Not assigned	Not observed	Not assigned
3122	3064	vPhH	3134	Not observed	vPhH
3127	3167	vPhH	3167	Not observed	vPhH
3357	3326	vNH	Not assigned	Not observed	Not assigned

Table S2. Predicted normalised intensity ranges and depolarisation ratios obtained from simulated angle-resolved polarised Raman spectra.

Facet	Vibrational mode	Normalised intensity range	Depolarisation ratio
(001)	OH Wag	0.012	0.018
	CH_3 Symmetric	0.645	0.009
	CH_3 Asymmetric	0.943	0.045
	NH Stretch	0.638	0.001
(011)	OH Wag	0.009	0.052
	CH_3 Symmetric	0.169	0.698
	CH_3 Asymmetric	0.934	0.055
	NH Stretch	0.643	0.030
$(0\bar{1}1)$	OH Wag	0.009	0.052
	CH_3 Symmetric	0.170	0.698
	CH_3 Asymmetric	0.934	0.055
	NH Stretch	0.642	0.031
(010)	OH Wag	0.004	0.076
	CH_3 Symmetric	0.414	0.031
	CH_3 Asymmetric	0.945	0.043
	NH Stretch	0.648	0.044

Table S3. Measured interfacial angle of all samples.

Sample Crystal	Experimental Facet	Experimental Angle (°)
**1**	**A - B**	34.36
	**A - C**	36.95
**2**	**A - B**	35.88
	**A - C**	35.44
**3**	**A - B**	35.11
	**A - C**	29.18
**4**	**A - B**	38.50
	**A - C**	31.12
**5**	**A - B**	33.65
	**A - C**	39.81

Section S1. Calculation of Raman tensors

For solids, the Raman activity tensors $\boldsymbol{\alpha}_{\Gamma j}$ of the Γ-point phonon modes are obtained from the derivative of the energy-dependent dielectric function $\boldsymbol{\varepsilon}(E)$ with respect to displacement amplitude $Q_{\Gamma j}$:

$$\boldsymbol{\alpha}_{\Gamma j} = \frac{\partial \boldsymbol{\varepsilon}(E)}{\partial Q_{\Gamma j}} \approx \frac{\Delta \boldsymbol{\varepsilon}(E)}{\Delta Q_{\Gamma j}}$$

The $Q_{\Gamma j}$ are related to the atomic displacement $\Delta \boldsymbol{r}_k$ of the kth atom in the primitive unit cell according to:

$$\Delta \boldsymbol{r}_k(Q_{\Gamma j}) = Q_{\Gamma j} \boldsymbol{X}_{k,\Gamma j} = Q_{\Gamma j} \frac{\boldsymbol{W}_{k,\Gamma j}}{\sqrt{m_k}}$$

where $\boldsymbol{X}_{k,\Gamma j}$ is the component of the "eigendisplacement" on the atom k obtained by dividing the corresponding component of the phonon eigenvector $\boldsymbol{W}_{k,\Gamma j}$ by the square root of the atomic mass m_k.

We considered several different approaches for calculating the $\boldsymbol{\varepsilon}(E)$. We first performed calculations using the density-functional perturbation theory (DFPT) routines in VASP. These calculations are relatively straightforward, but the VASP implementation can only determine the high-frequency dielectric constant $\boldsymbol{\varepsilon}_\infty = \boldsymbol{\varepsilon}(E = 0)$. The $\boldsymbol{\varepsilon}_\infty$ can be evaluated at one of two levels of approximation: the independent-particle approximation (IPA), which neglects changes to the cell-periodic part of the potential (so-called local-field effects), and a more sophisticated treatment that includes these effects. In both cases, the $\boldsymbol{\alpha}_{\Gamma j}$ based on changes in the $\boldsymbol{\varepsilon}_\infty$ assumes that the derivative of the dielectric function at the measurement laser wavelength of λ = 532 nm is comparable to that at E = 0, which is termed the far-from-resonance approximation (FFRA). We then also performed additional calculations to evaluate the full energy-dependent dielectric function $\boldsymbol{\varepsilon}(E)$ using the "sum over states" implementation of the IPA. This method has the advantage of accounting for the energy dependence of the dielectric response, but requires large numbers of unoccupied (virtual) electronic states, which make the calculations technically more involved, and also does not include for local-field effects.

Convergence of the DFPT Raman spectra with respect to $\boldsymbol{k}$-point sampling

While, as noted in the main text, Γ-point $\boldsymbol{k}$-point sampling was found to be sufficient for the structural optimisation and subsequent phonon calculations, further explicit testing determined that denser $\boldsymbol{k}$-point sampling was required to converge the $\boldsymbol{\varepsilon}_\infty$ calculated with DFPT.

Table S4 shows the non-zero elements of the $\boldsymbol{\varepsilon}_\infty$ calculated with Γ-point sampling and with denser 1 × 2 × 3, 2 × 3 × 5 and 3 × 4 × 7 $\boldsymbol{k}$-point meshes. We find that Γ-point sampling is sufficient to converge the three diagonal elements ε_∞^{xx}, ε_∞^{yy} and ε_∞^{zz} of the $\boldsymbol{\varepsilon}_\infty$ tensor to within 1.4% of the values obtained with denser sampling, but the much smaller $\varepsilon_\infty^{xz} = \varepsilon_\infty^{zx}$ component has a larger error of 13%. With denser 1 × 2 × 3 $\boldsymbol{k}$-point mesh these errors are reduced to <0.5%, and the 2 × 3 × 5 mesh is fully converged with respect to the denser 3 × 4 × 7 mesh.

With our chosen displacement step of ΔQ = 3.5 × 10^{-2} amu$^{1/2}$ Å and Γ-point $\boldsymbol{k}$-point sampling, the average and maximum component-wise derivatives of the $\boldsymbol{\varepsilon}_0$ over all 120 of the Raman-active bands, computed including local-field effects, are 4.08 × 10^{-4} ± 9.3 × 10^{-4} and 1.53 × 10^{-2} respectively (**Table S5**). Similar

results are obtained for the calculations with denser 1 × 2 × 3 and 2 × 3 × 5 $\boldsymbol{k}$-point sampling, and with $\boldsymbol{\varepsilon}_\infty$ calculated within the IPA. These comparisons suggest that we ideally require convergence of the $\boldsymbol{\varepsilon}_\infty$ to four decimal places, which the data in **Table S4** shows is obtained with the 2 × 3 × 5 $\boldsymbol{k}$-point mesh.

Table S6 additionally shows the independent components of the Raman tensors, computed using DFPT including local-field effects, of the 12 selected bands used for facet identification based on the changes in band intensity as a function of in-plane rotation angle. The average and largest components are 1.58 and 10.7 $Å^2$ $amu^{1/2}$, respectively. Taking the values obtained with the 2 × 3 × 5 $\boldsymbol{k}$-point mesh as a reference, we obtain maximum absolute differences of 1.83, 0.13 and 4 × 10^{-2} with Γ-point, 1 × 2 × 3 and 3 × 4 × 7 $\boldsymbol{k}$-point meshes. These comparisons confirm that Γ-point sampling is insufficiently converged, but that the error with respect to $\boldsymbol{k}$-point sampling for the denser 2 × 3 × 5 mesh is 2-3 orders of magnitude lower than the average and largest components of the $\boldsymbol{\alpha}_{\Gamma j}$. Given the much higher computational cost of calculations with the 3 × 4 × 7 $\boldsymbol{k}$-point mesh (and larger meshes), the 2 × 3 × 5 mesh appears to be a very reasonable compromise.

To explore this further, and to qualitatively analyse the impact of $\boldsymbol{k}$-point sampling on the simulated spectra, we simulated powder-averaged Raman spectra obtained using the $\boldsymbol{\varepsilon}_0$ including local-field effects and calculated with Γ-point sampling and denser 1 × 2 × 3, and 2 × 3 × 5 sampling meshes (**Figure S14**). Increasing the $\boldsymbol{k}$-point sampling from the Γ-point to the 1 × 2 × 3 mesh leads to some notable changes in the band intensities below 250 cm^{-1}, from 1250-1750 cm^{-1} and from 2750-3500 cm^{-1}. Increasing the mesh again to the fully converged 2 × 3 × 5 mesh then leads to minor further changes, the most prominent of which are below 250 cm^{-1}.

Finally, we also compared polar plots obtained for the four groups of bands analysed in the text at the (001), (011) and $(0\bar{1}1)$ surfaces, each obtained with three different $\boldsymbol{k}$-point meshes (**Figures S15-S17**). We find that the calculations using Γ-point sampling and the denser 1 × 2 × 3 and 2 × 3 × 5 $\boldsymbol{k}$-point meshes predict very similar behaviour at all three surfaces, such that the differences between are well within the resolution of the experimental measurements.

Convergence of the IPA energy-dependent Raman spectra with respect to the number of virtual states

In addition to $\boldsymbol{k}$-point sampling, calculations of the energy-dependent $\boldsymbol{\varepsilon}(E)$ must also be converged with respect to the number of virtual states included in the summation over electronic transitions. The IPA $\boldsymbol{\varepsilon}_\infty$ computed with DFPT are not sensitive to the number of virtual states and thus serve as a reference point for checking convergence.

Table S7 compares the independent components of the IPA $\boldsymbol{\varepsilon}_0$ calculated using DFPT and the sum-over-states method with up to a 20× excess of virtual states in the calculation, all using the converged 2 × 3 × 5 $\boldsymbol{k}$-point sampling. With a 1× excess of virtual states, i.e. with the same number of empty and filled states, the diagonal $\varepsilon_\infty^{\alpha\alpha}$ components of the $\boldsymbol{\varepsilon}_\infty$ are ~5-7% lower than the DFPT reference. A larger 4× and 10× excess improves these errors to <1 and <0.1% respectively. On the other hand, the smaller ε_∞^{xz} component is underestimated by 95% with a 1× excess of virtual states, and a 5× excess is required to improve this to <5%. Even with the largest 20× excess of states we tested, the three $\varepsilon_\infty^{\alpha\alpha}$ and the ε_∞^{xz} are 0.04 and 1% lower, respectively, than the DFPT reference. These correspond to errors on the order of 10^{-3}-10^{-4} ε_0, which

is above the threshold for absolute convergence (the average and maximum changes in the component-wise derivatives of the $\boldsymbol{\varepsilon}_\infty$ for the IPA DFPT calculations are also shown in **Table S5**). This suggests it is not technically feasible to converge the $\boldsymbol{\varepsilon}_\infty$ with respect to the DFPT reference to the level of precision we ideally require.

However, we find that in practice such strict convergence is not necessary. **Figure S18** compares simulated powder-averaged Raman spectra obtained from IPA $\boldsymbol{\varepsilon}_\infty$ calculated with DFPT and the sum-over-states method with a 2-5× excess of virtual states. As observed for the $\boldsymbol{k}$-point convergence, the simulated powder spectra converge on the DFPT reference spectrum as the number of virtual states included in the calculation is increased, such that at with a 5× excess the largest errors are confined below 250 cm^{-1} and the simulated spectrum is visually indistinguishable from the DFPT result at higher wavenumbers.

Figures S19-S21 compare polar plots for the four band groups and three surfaces of interest obtained from calculations using the same set of methods. The polar plots largely mirror the convergence behaviour of the powder spectra. At all three surfaces, a 3× excess of virtual states is sufficient to reproduce the polarisation behaviour of three of the four band groups predicted with DFPT to a degree that would be indistinguishable within experimental resolution, and while the fourth group, with $\bar{\nu}$ = 385/389 cm^{-1}, shows larger differences to the reference calculations they are still within experimental resolution. We therefore infer that the IPA sum-over-states calculations are sufficiently, if not absolutely, converged with a 5× excess of virtual states.

Comparison of the DFPT far-from-resonance and IPA far-from-resonance and energy-dependent Raman spectra

Having checked the convergence of the DFPT $\boldsymbol{\varepsilon}_\infty$ with respect to $\boldsymbol{k}$-point sampling, and the IPA sum-over-states $\boldsymbol{\varepsilon}_\infty$ with respect to the number of virtual states, we now compare simulations using three different treatments of the dielectric response: calculations using the FFRA with $\boldsymbol{\varepsilon}_\infty$ obtained from DFPT calculations including local-field effects; calculations using the FFRA with $\boldsymbol{\varepsilon}_\infty$ obtained within the IPA using the sum-over-states method; and calculations using the dielectric response at the laser wavelength λ = 532 nm (E = 2.33 eV), again obtained within the IPA using the sum-over-states method.

Figure S22 compares the normalised powder-averaged spectra obtained with the three methods. The major qualitative differences between the three methods is in the band intensities relative to the features between 1500-1750 cm^{-1}. Both sets of IPA calculations predict the high-frequency features above 2750 cm^{-1} to have much lower relative intensities than the most intense feature between 1500-1750 cm^{-1}, whereas the DFPT calculations including local-field effects predict the high-frequency features to be more intense such that the features in the two ranges show comparable intensity. This much better reflects the measured band intensities. The spectra obtained using the IPA dielectric response with either the $\boldsymbol{\varepsilon}_\infty$ or the $\boldsymbol{\varepsilon}(E$ = 2.33 eV) are generally very similar, with the latter resulting in a small reduction in intensity across the spectrum relative to the most intense feature at 1611 cm^{-1}. This suggests that the FFRA is reasonable for this system, as one would expect given the wide bandgap E_g relative to the measurement laser wavelength (E_g = 3.43 eV direct and 3.35 eV indirect in our GGA calculations).

Despite the large differences in relative band intensities, the three treatments of the dielectric response predict remarkably similar polarisation behaviour (**Figures S23-S25**). At the (001) surface, the main

qualitative differences are in the polarisation behaviour at the minima. This is particularly noticeable for the feature at 3357/3362 cm^{-1}, where the calculations including local-field effects predict that the band intensity falls to zero at some angles, while both calculations using the IPA predict a non-zero minimum. Broadly similar behaviour is predicted at the (011) and $(0\bar{1}1)$surfaces, with the exception that the behaviour of the low-frequency band group at 385/389 cm^{-1} shows larger tilts about ϕ = 90 ° when local-field effects are included in the calculation. This again better matches the experimental polarisation behaviour of this feature relative to the three groups of higher-frequency bands.

Overall, this analysis indicates that for this system the inclusion of local-field effects is much more important for correctly capturing the experimental band intensities and polarisation behaviour than accounting for the energy-dependence of the dielectric response. This is clearly seen by comparing the simulated angle-resolved polarised Raman spectra of the three surfaces as a function of in-plane rotation angle in **Figures S26-S28** to **Figure 5** in the text. Based on this, we therefore conclude that DFPT calculations using the FFRA and accounting for local-field are the most efficient and accurate approach to simulating the Raman activity tensors of wide-bandgap molecular solids.

Table S4. Non-zero components of the high-frequency dielectric constant $\boldsymbol{\varepsilon}_{\infty}$ of paracetamol computed using density-functional perturbation theory (DFPT) and four different $\boldsymbol{k}$-point sampling meshes. Two sets of values are shown, computed within the independent-particle approximation (IPA) and including local-field effects (LF). Where applicable, percentage errors with respect to the largest sampling mesh are shown.

$\boldsymbol{k}$-Points	$\boldsymbol{\varepsilon}_{\infty}$ (IPA, DFPT) / ε_0				$\boldsymbol{\varepsilon}_{\infty}$ (LF, DFPT) / ε_0			
	$\varepsilon_{\infty}^{xx}$	$\varepsilon_{\infty}^{yy}$	$\varepsilon_{\infty}^{zz}$	$\varepsilon_{\infty}^{xz} = \varepsilon_{\infty}^{zx}$	$\varepsilon_{\infty}^{xx}$	$\varepsilon_{\infty}^{yy}$	$\varepsilon_{\infty}^{zz}$	$\varepsilon_{\infty}^{xz} = \varepsilon_{\infty}^{zx}$
Γ	3.4914 (+0.13%)	3.0765 (-0.49%)	3.3582 (+1.19%)	0.0032 (-83.16%)	2.8671 (+0.25%)	2.5247 (-0.49%)	2.7795 (+1.35%)	0.1106 (-13.13%)
1 × 2 × 3	3.4949 (+0.23%)	3.0920 (+0.01%)	3.3188	0.0178 (-6.73%)	2.8670 (+0.24%)	2.5373 (+0.01%)	2.7424 (<0.01%)	0.1268 (-0.43%)
2 × 3 × 5	3.4870	3.0916	3.3188	0.0191	2.8600	2.5371	2.7425	0.1274
3 × 4 × 7	3.4870	3.0916	3.3188	0.0191 (-0.02%)	2.8600	2.5371	2.7425	0.1274

Table S5. Average and maximum component-wise derivatives of the high-frequency dielectric constant $\boldsymbol{\varepsilon}_{\infty}$ over the Raman-active bands for Raman activity tensor calculations performed using density-functional perturbation theory (DFPT) with three different $\boldsymbol{k}$-point sampling meshes and within the independent-particle approximation (IPA) or including local-field effects (LF).

	Ave $\Delta\boldsymbol{\varepsilon}_{\infty}$ / 10^{-4} ε_0	Max. $\Delta\boldsymbol{\varepsilon}_{\infty}$ / 10^{-2} ε_0
Γ (LF, DFPT)	4.08 ± 9.3	1.53
1 × 2 × 3 (LF, DFPT)	3.93 ± 8.67	1.22
2 × 3 × 5 (LF, DFPT)	3.93 ± 8.65	1.22
Γ (IPA, DFPT)	6.01 ± 12.2	1.34
1 × 2 × 3 (IPA, DFPT)	5.81 ± 11.6	1.03
2 × 3 × 5 (IPA, DFPT)	5.81 ± 11.5	1.03

Table S6. Independent components of the Raman tensors $\boldsymbol{\alpha}_{\Gamma j}$ for selected bands j computed using density-functional perturbation theory (DFPT), including local-field effects, and four different $\boldsymbol{k}$-point sampling meshes.

ν / cm^{-1}	$\boldsymbol{k}$-points	$\boldsymbol{\alpha}$ / (Å^2 amu$^{1/2}$)					
		α_{xx}	α_{yy}	α_{zz}	α_{xy}	α_{xz}	α_{yz}
385	Γ	-0.2291	-1.4068	0.3074	0	0.2201	0
	1 × 2 × 3	-0.2098	-1.6628	0.1676	0	0.3153	0
	2 × 3 × 5	-0.1791	-1.6686	0.1693	0	0.3087	0
	3 × 4 × 7	-0.1790	-1.6687	0.1705	0	0.3088	0
389	Γ	0.0002	0.0001	-0.0001	-0.9330	-0.0002	-0.6697
	1 × 2 × 3	0.0004	0.0010	-0.0026	-0.9078	-0.0003	-0.6307
	2 × 3 × 5	-0.0071	0.0133	0.0012	-0.9031	-0.0021	-0.6814
	3 × 4 × 7	-0.0012	-0.0031	-0.0002	-0.9152	-0.0011	-0.6799
2984	Γ	8.7327	10.6483	10.7142	0	2.2382	0
	1 × 2 × 3	8.7509	10.2772	10.5989	0	2.2777	0
	2 × 3 × 5	8.7228	10.2757	10.6072	0	2.2766	0
	3 × 4 × 7	8.7219	10.2772	10.6103	0	2.2791	0
3113	Γ	0.0000	0.0002	-0.0002	-2.7599	0.0001	-2.6346
	1 × 2 × 3	-0.0002	0.0013	-0.0006	-2.7103	-0.0002	-2.5681
	2 × 3 × 5	0.0010	0.0037	-0.0007	-2.7002	-0.0013	-2.5840
	3 × 4 × 7	0.0005	-0.0008	-0.0001	-2.6930	0.0002	-2.5845
3113	Γ	0.0014	0.0010	-0.0001	-1.6563	0.0002	-1.1343
	1 × 2 × 3	0.0008	0.0007	0.0010	-1.5766	-0.0001	-0.9744
	2 × 3 × 5	0.0003	-0.0013	-0.0001	-1.6700	0.0001	-1.0544
	3 × 4 × 7	0.0005	0.0014	0.0000	-1.6373	-0.0001	-1.0147
3120	Γ	-8.7503	0.5398	-0.4078	0	2.9623	0
	1 × 2 × 3	-8.5094	0.6089	0.0096	0	2.8154	0
	2 × 3 × 5	-8.3830	0.5970	0.0146	0	2.8089	0
	3 × 4 × 7	-8.3824	0.5970	0.0149	0	2.8084	0
3120	Γ	10.6938	0.4000	-0.2618	0	-4.2099	0
	1 × 2 × 3	10.4260	0.5660	-0.0112	0	-4.1661	0
	2 × 3 × 5	10.3589	0.5593	-0.0105	0	-4.1685	0
	3 × 4 × 7	10.3573	0.5620	-0.0115	0	-4.1682	0
3357	Γ	0.0014	0.0009	0.0000	0.5243	0.0002	-0.7333
	1 × 2 × 3	-0.0001	-0.0013	0.0009	0.4834	0.0001	-0.9223
	2 × 3 × 5	-0.0005	-0.0011	0.0002	0.4758	0.0005	-0.9022
	3 × 4 × 7	0.0001	-0.0006	-0.0001	0.4914	0.0001	-0.8936
3362	Γ	-10.5561	-1.1873	-4.4807	0	5.7490	0
	1 × 2 × 3	-9.8455	-1.2532	-2.6624	0	4.4759	0
	2 × 3 × 5	-9.8773	-1.2425	-2.6497	0	4.4323	0
	3 × 4 × 7	-9.8723	-1.2519	-2.6499	0	4.4328	0

Table S7. Non-zero components of the high-frequency dielectric constant $\boldsymbol{\varepsilon}_\infty = \boldsymbol{\varepsilon}(E=0)$ of paracetamol computed with the independent-particle approximation (IPA) with increasing numbers of virtual states included in the calculation. The numbers of virtual states are expressed as multiples of the number of occupied states. The equivalent value of the $\boldsymbol{\varepsilon}_\infty$ computed using density-functional perturbation theory (DFPT), which does not depend on the number of virtual states, is also provided as a reference. For each calculation, percentage errors with respect to the DFPT reference are shown.

Virtual states	$\boldsymbol{\varepsilon}_\infty$ / ε_0			
	ε_∞^{xx}	ε_∞^{yy}	ε_∞^{zz}	$\varepsilon_\infty^{xz} = \varepsilon_\infty^{zx}$
DFPT	3.4870	3.0916	3.3188	0.0191
1 ×	3.3006<br>(-5.35%)	2.8727<br>(-7.08%)	3.1187<br>(-6.03%)	0.0010<br>(-94.6%)
2 ×	3.4336<br>(-1.53%)	3.0183<br>(-2.37%)	3.2594<br>(-1.79%)	0.0084<br>(-55.8%)
3 ×	3.4598<br>(-0.78%)	3.0567<br>(-1.13%)	3.2897<br>(-0.88%)	0.0143<br>(-25.24%)
4 ×	3.4706<br>(-0.47%)	3.0722<br>(-0.63%)	3.3020<br>(-0.51%)	0.0167<br>(-12.44%)
5 ×	3.4760<br>(-0.31%)	3.0794<br>(-0.39%)	3.3079<br>(-0.33%)	0.0182<br>(-4.53%)
10 ×	3.4838<br>(-0.09%)	3.0884<br>(-0.1%)	3.3158<br>(-0.09%)	0.0188<br>(-1.27%)
20 ×	3.4857<br>(-0.04%)	3.0904<br>(-0.04%)	3.3175<br>(-0.04%)	0.0189<br>(-1%)

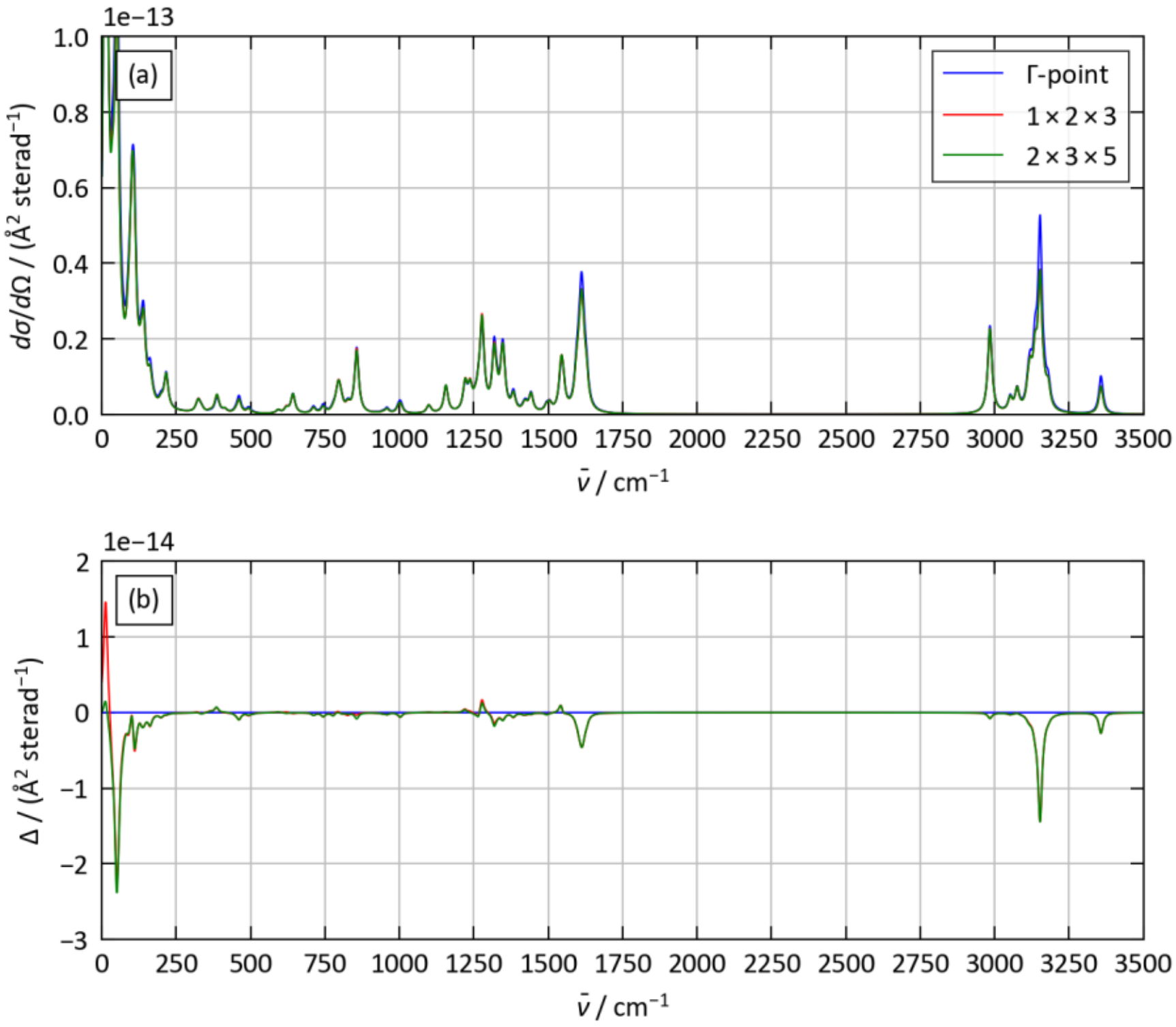

Figure S14. Predicted powder Raman spectra of paracetamol using Raman-activity tensors calculated in the far-from-resonance approximation, with high-frequency dielectric constants $\boldsymbol{\varepsilon}_{\infty}$ obtained from density-functional theory (DFPT) calculations including local-field effects and with three different $\boldsymbol{k}$-point sampling meshes. Plot (a) shows the spectra, and plot (b) shows the differences in normalised intensity of the two spectra computed on denser $\boldsymbol{k}$-point sampling relative to the spectrum computed with Γ-point sampling.

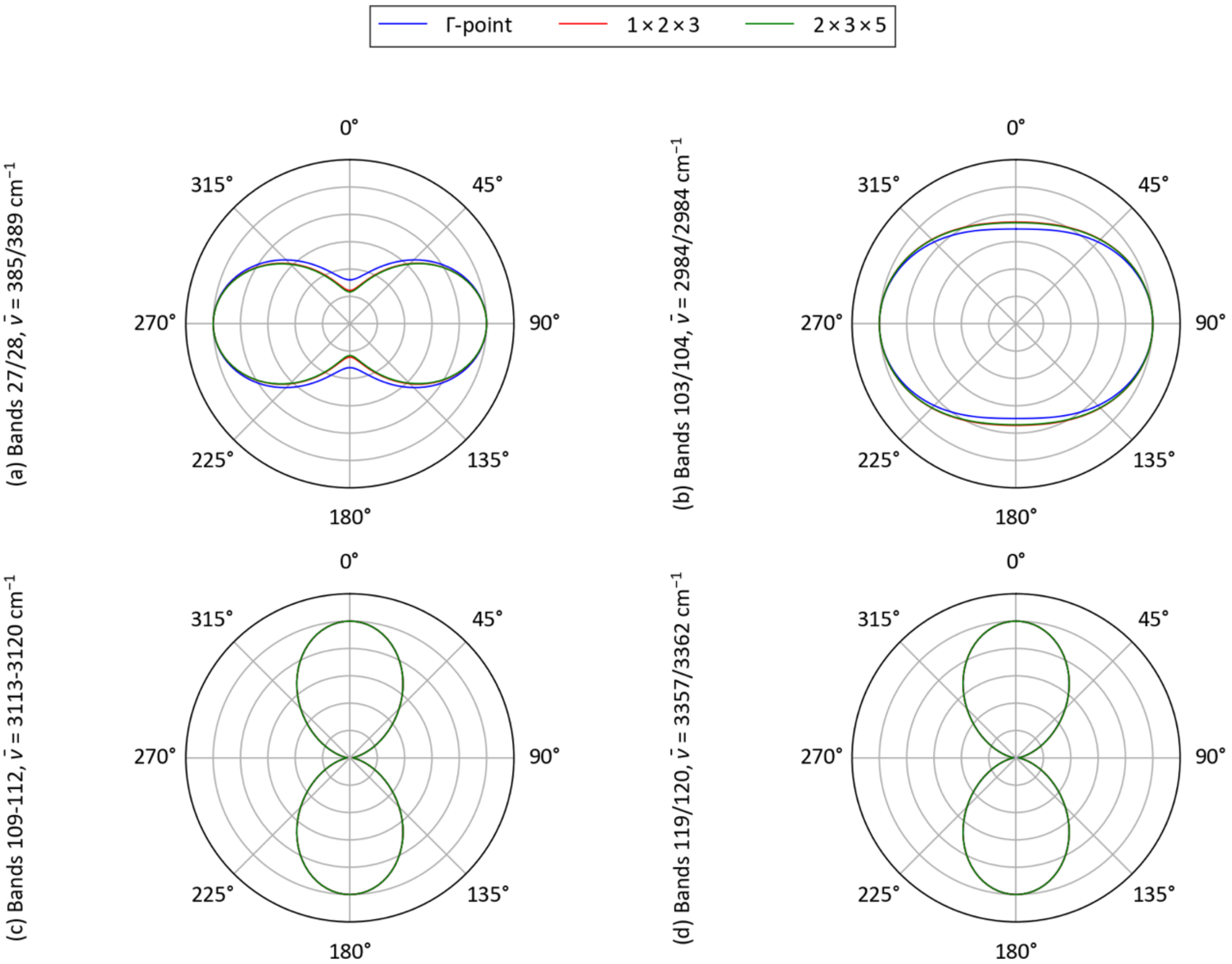

Figure S15. Predicted angle dependence of the relative intensities of selected spectral features in angle-resolved polarised Raman measurements of the (001) surface of paracetamol, calculated in the far-from-resonance approximation with high-frequency dielectric constants $\boldsymbol{\varepsilon}_{\infty}$ the density-functional theory (DFPT) including local-field effects. Each plot shows the relative intensities predicted with three different $\boldsymbol{k}$-point sampling meshes.

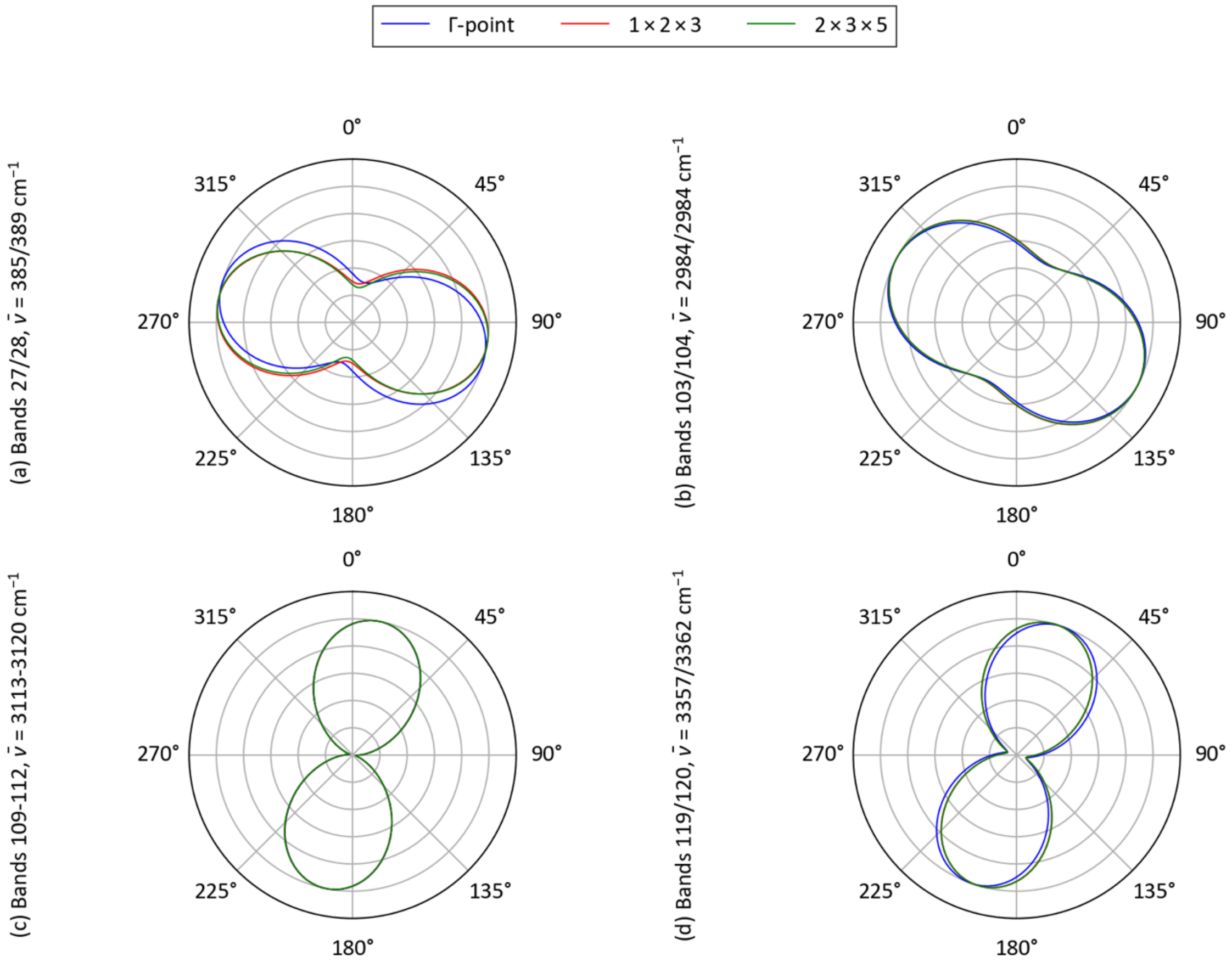

Figure S16. Predicted angle dependence of the relative intensities of selected spectral features in angle-resolved polarised Raman measurements of the (011) surface of paracetamol, calculated in the far-from-resonance approximation with high-frequency dielectric constants $\boldsymbol{\varepsilon}_{\infty}$ the density-functional theory (DFPT) including local-field effects. Each plot shows the relative intensities predicted with three different $\boldsymbol{k}$-point sampling meshes.

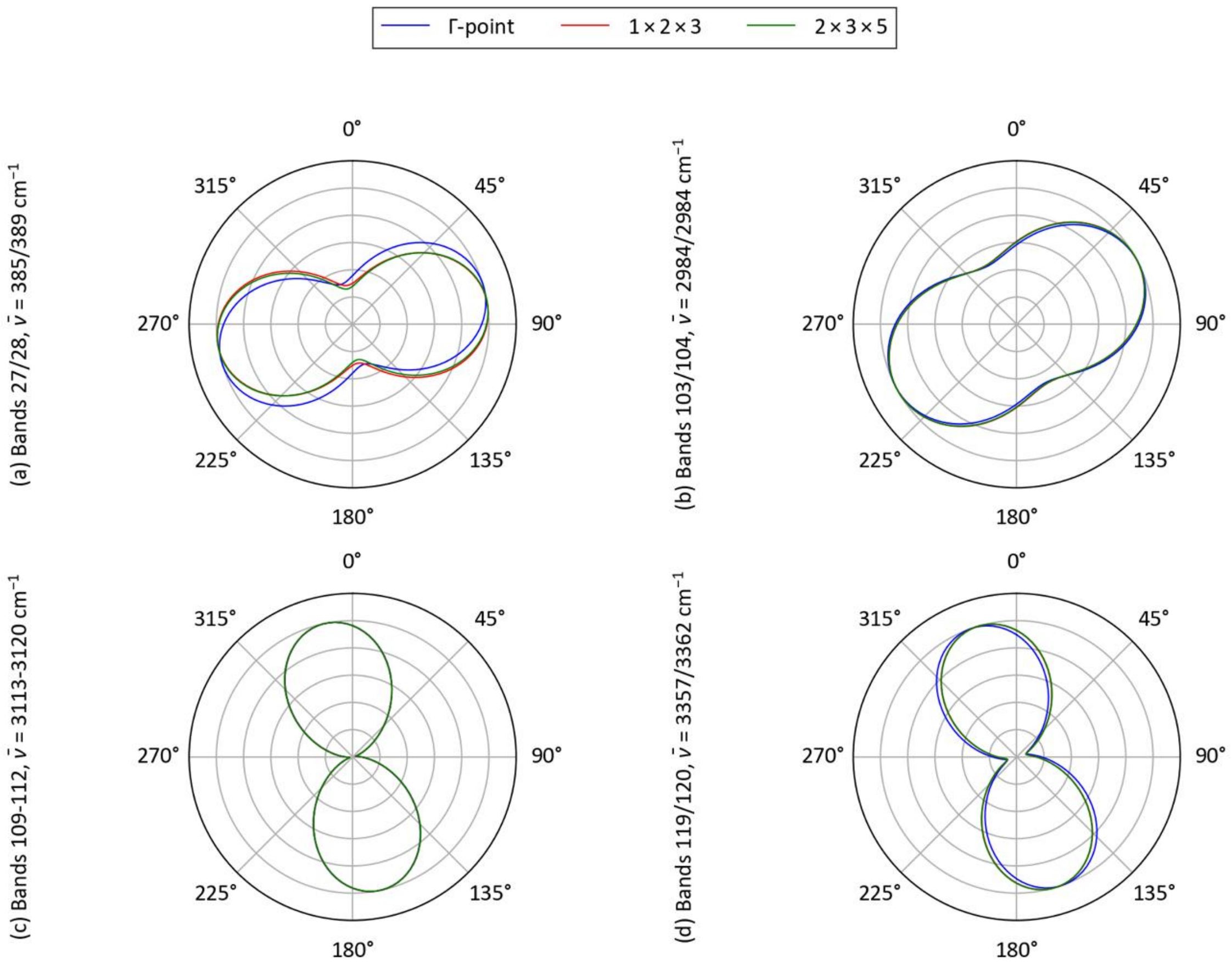

Figure S17. Predicted angle dependence of the relative intensities of selected spectral features in angle-resolved polarised Raman measurements of the $(0\bar{1}1)$ surface of paracetamol, calculated in the far-from-resonance approximation with high-frequency dielectric constants $\boldsymbol{\varepsilon}_{\infty}$ the density-functional theory (DFPT) including local-field effects. Each plot shows the relative intensities predicted with three different $\boldsymbol{k}$-point sampling meshes.

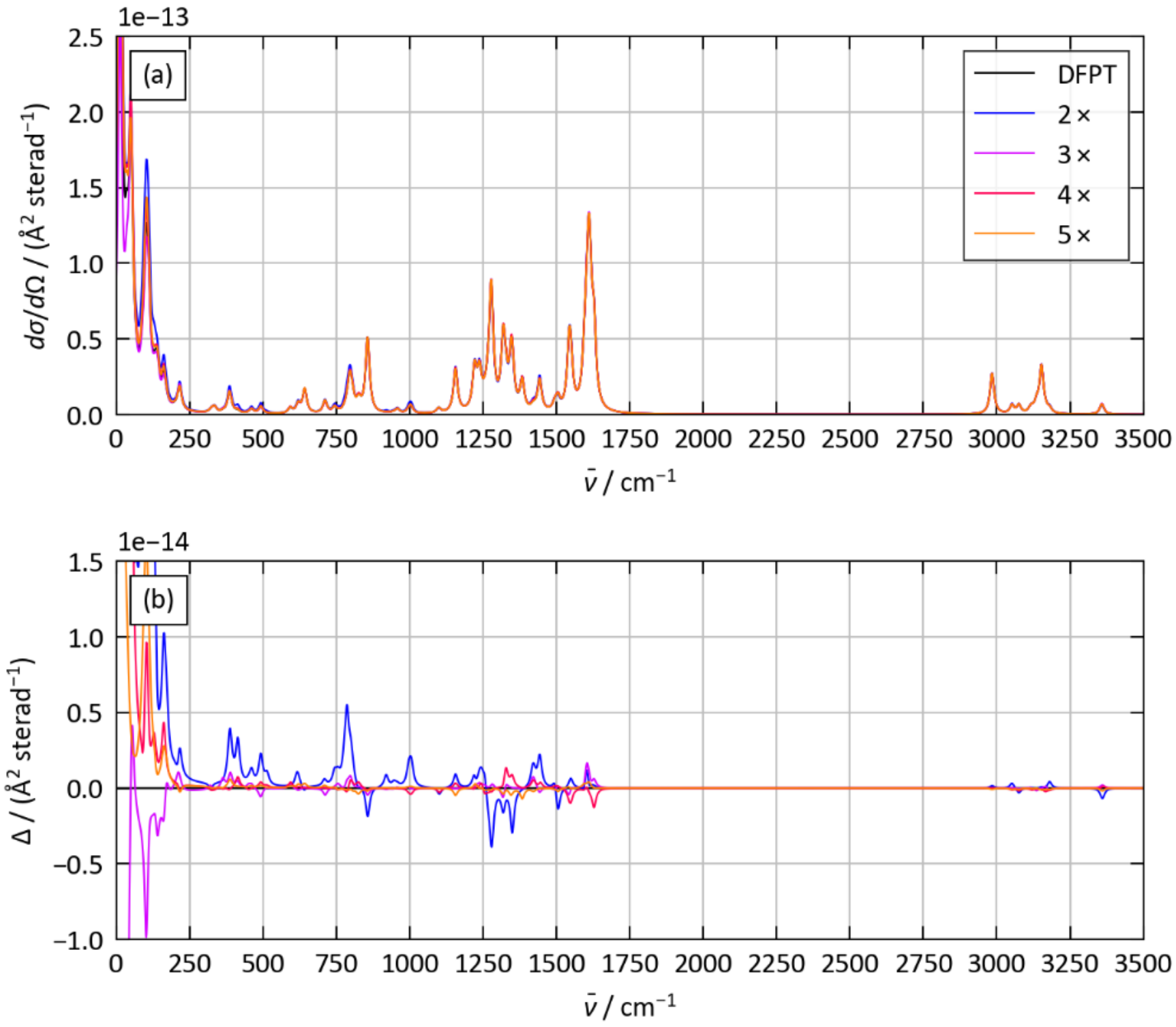

Figure S18. Predicted powder Raman spectra of paracetamol using Raman-activity tensors calculated in the far-from-resonance approximation, with high-frequency dielectric constants $\boldsymbol{\varepsilon}_{\infty}$ obtained from independent-particle approximation calculations using either density-functional perturbation theory (DFPT) or the "sum over states" method with a 1-5× excess of virtual states. Spectra obtained using the latter method are colour coded from blue to orange, corresponding to increasing numbers of virtual states, and the DFPT reference spectrum is shown in black. Plot (a) shows the spectra, and plot (b) shows the differences in normalised intensity of the spectra computed with the sum-over-states method relative to the spectrum computed with DFPT.

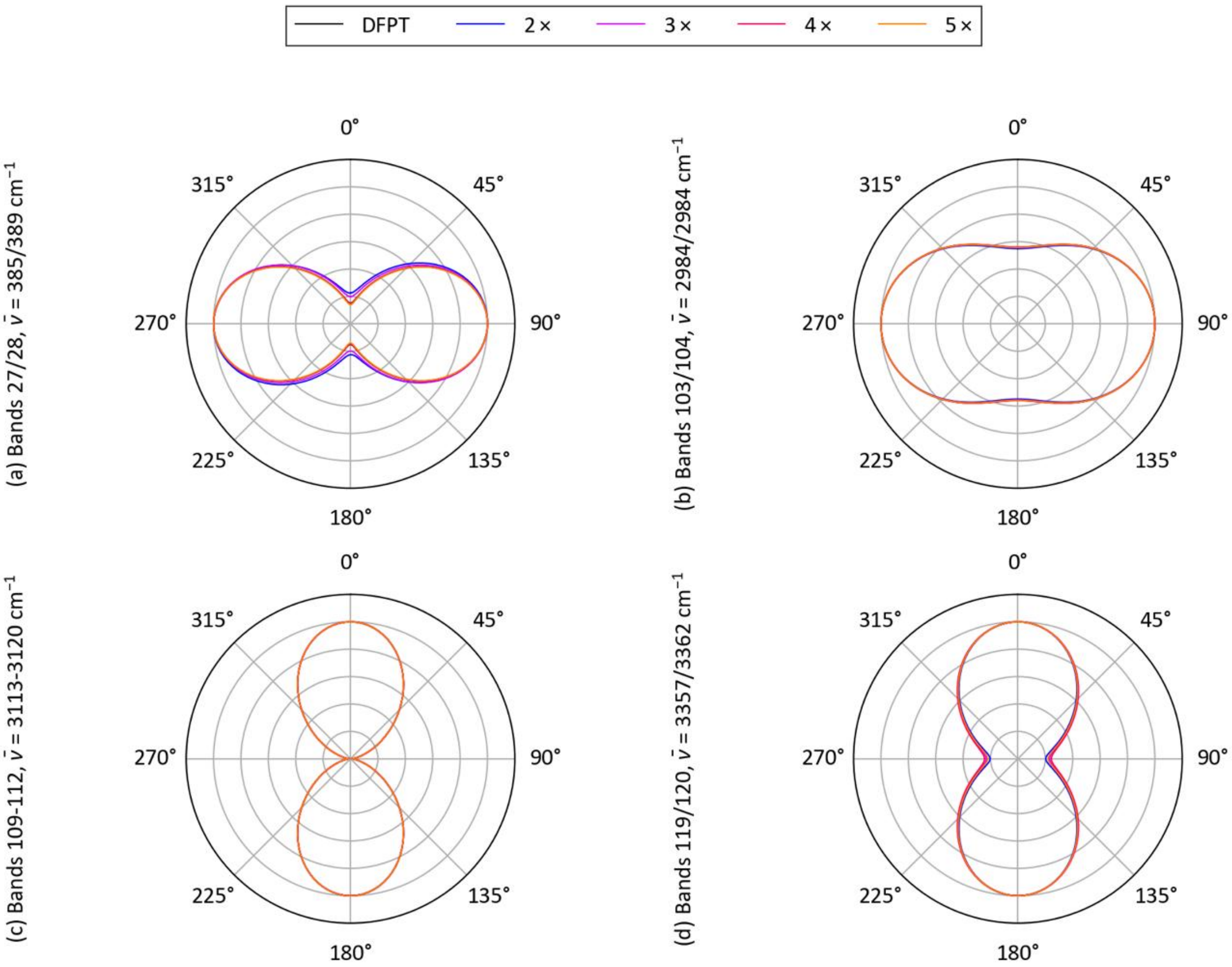

Figure S19. Predicted angle dependence of the relative intensities of selected spectral features in angle-resolved polarised Raman measurements of the (001) surface of paracetamol, calculated in the far-from-resonance approximation with the independent-particle approximation (IPA) high-frequency dielectric constants $\boldsymbol{\varepsilon}_{\infty}$. Each plot shows the relative intensities predicted from calculations using density-functional perturbation theory (DFPT) or the "sum-over-states" method with a 1-5× excess of virtual states. Polar plots obtained using the latter method are colour coded from blue to orange, corresponding to increasing numbers of virtual states, and the DFPT reference plot is shown in black.

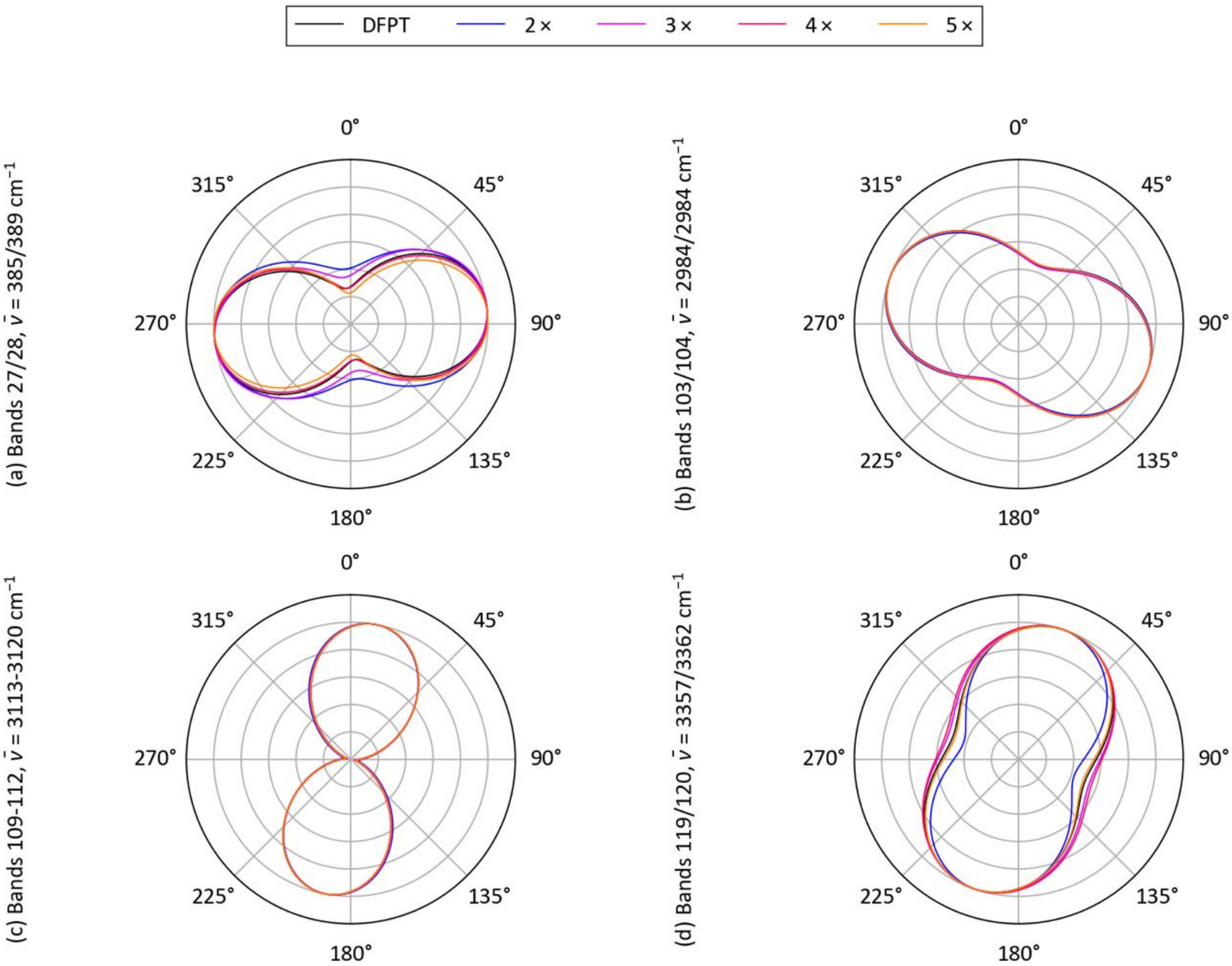

Figure S20. Predicted angle dependence of the relative intensities of selected spectral features in angle-resolved polarised Raman measurements of the (011) surface of paracetamol, calculated in the far-from-resonance approximation with the independent-particle approximation (IPA) high-frequency dielectric constants $\boldsymbol{\varepsilon}_{\infty}$. Each plot shows the relative intensities predicted from calculations using density-functional perturbation theory (DFPT) or the "sum-over-states" method with a 1-5× excess of virtual states. Polar plots obtained using the latter method are colour coded from blue to orange, corresponding to increasing numbers of virtual states, and the DFPT reference plot is shown in black.

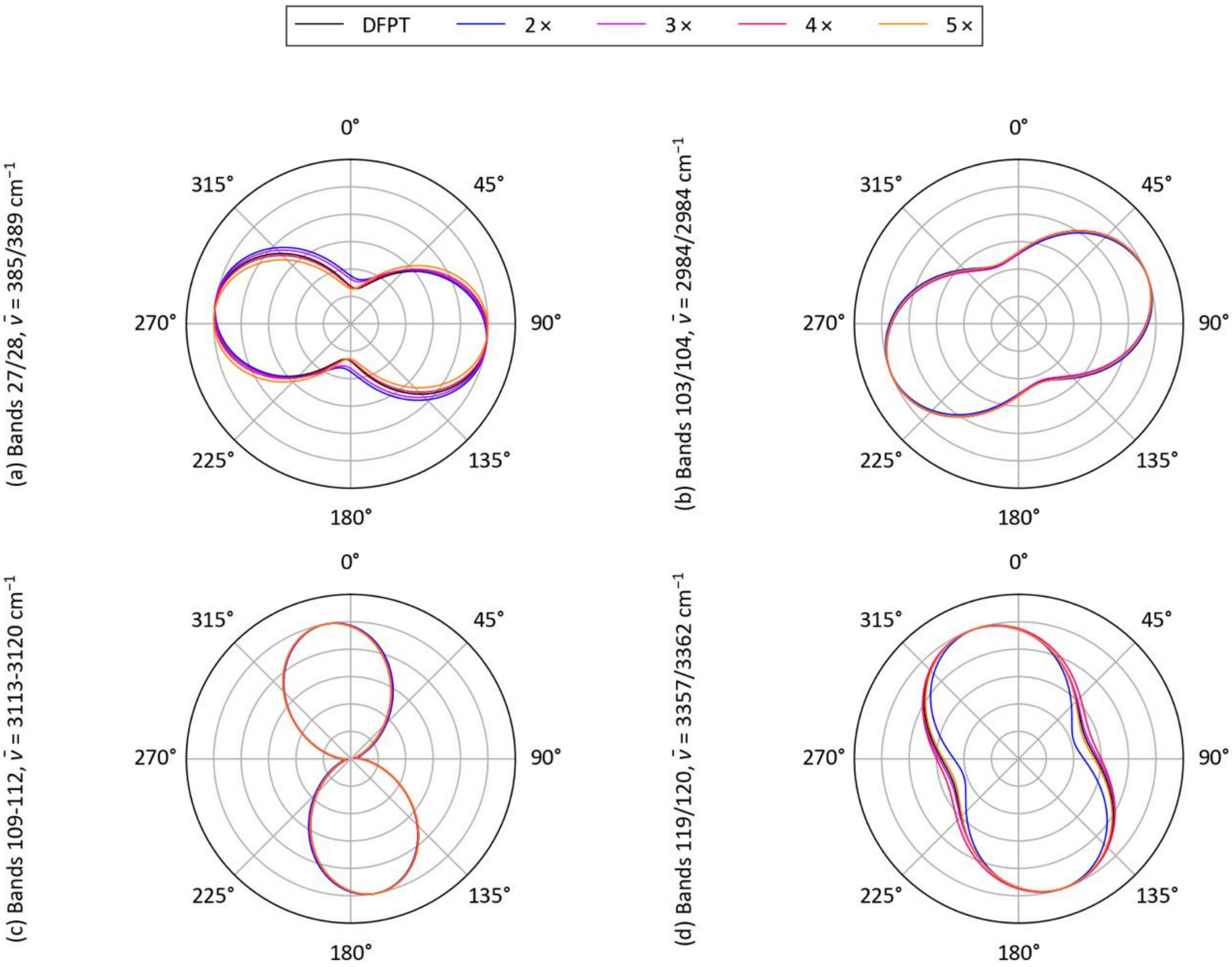

Figure S21. Predicted angle dependence of the relative intensities of selected spectral features in angle-resolved polarised Raman measurements of the $(0\bar{1}1)$ surface of paracetamol, calculated in the far-from-resonance approximation with the independent-particle approximation (IPA) high-frequency dielectric constants $\boldsymbol{\varepsilon}_{\infty}$. Each plot shows the relative intensities predicted from calculations using density-functional perturbation theory (DFPT) or the "sum-over-states" method with a 1-5× excess of virtual states. Polar plots obtained using the latter method are colour coded from blue to orange, corresponding to increasing numbers of virtual states, and the DFPT reference plot is shown in black.

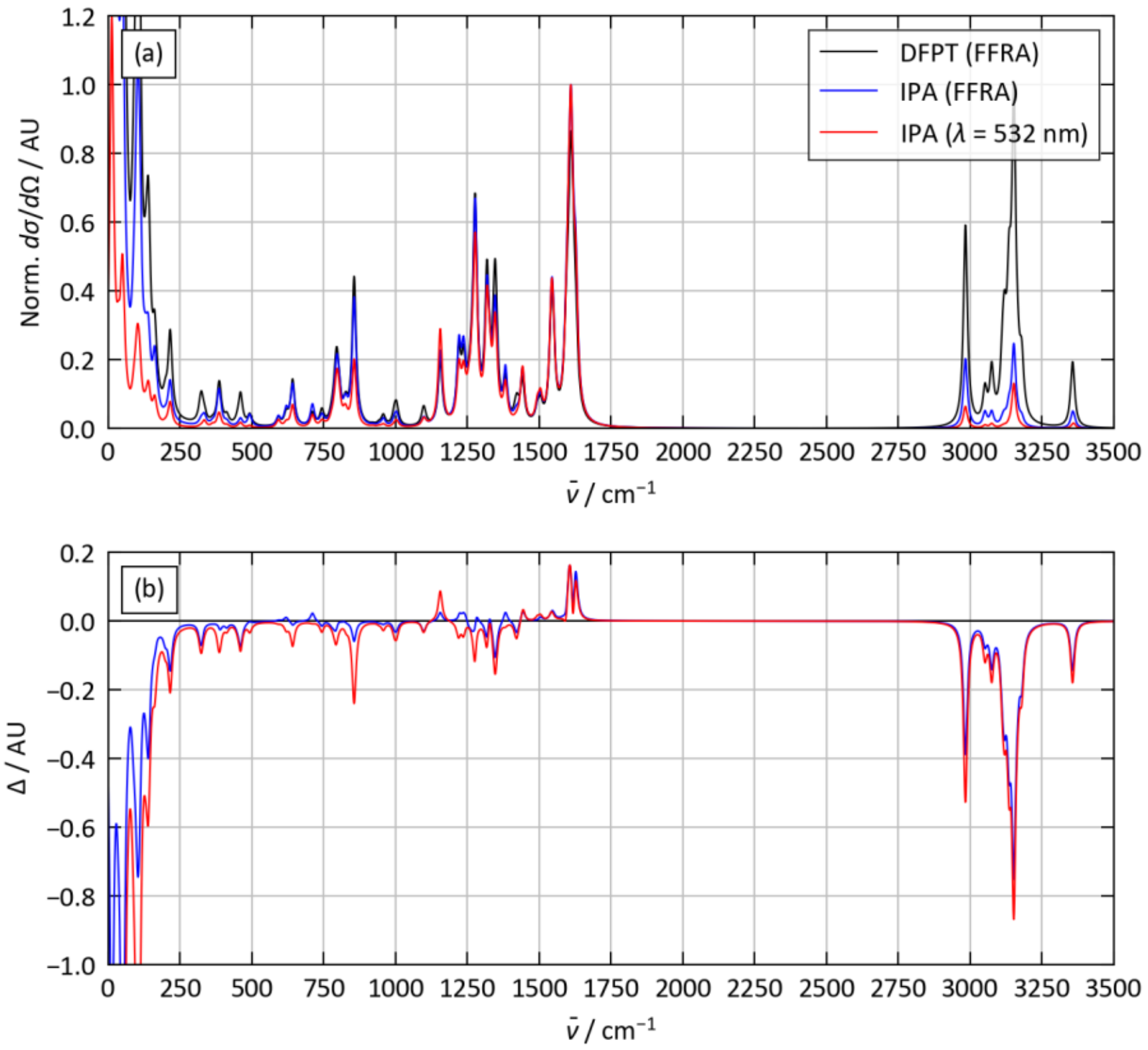

Figure S22. Predicted powder Raman spectra of paracetamol using Raman-activity tensors calculated using one of three methods: within the far-from-resonance approximation (FFRA) using high-frequency dielectric constants $\boldsymbol{\varepsilon}_{\infty}$ from density-functional perturbation theory (DFPT) including local-field effects; within the FFRA using $\boldsymbol{\varepsilon}_{\infty}$ from the independent-particle approximation (IPA); and within the IPA using the dielectric response $\boldsymbol{\varepsilon}(E)$ at the measurement laser wavelength λ = 532 nm (E = 2.33 eV). Plot (a) shows the spectra, and plot (b) shows the differences in intensity relative to the DFPT FFRA calculation. The three spectra are normalised relative to the highest band intensity at $\bar{\nu}$ > 500 cm^{-1}.

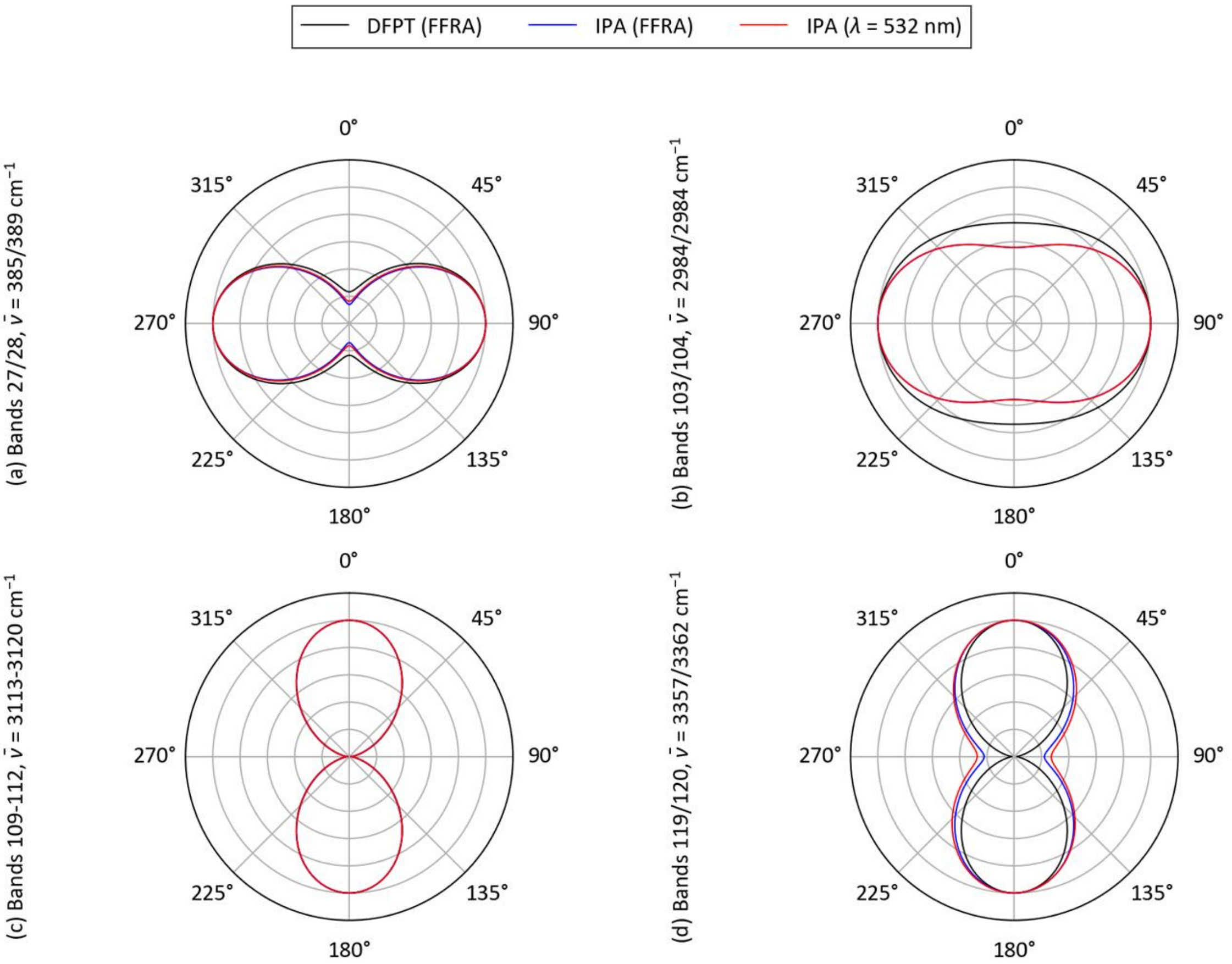

Figure S23. Predicted angle dependence of the relative intensities of selected spectral features in angle-resolved polarised Raman measurements of the (001) surface of paracetamol obtained with one of three methods: within the far-from-resonance approximation (FFRA) using high-frequency dielectric constants $\boldsymbol{\varepsilon}_{\infty}$ from density-functional perturbation theory (DFPT) including local-field effects; within the FFRA using $\boldsymbol{\varepsilon}_{\infty}$ from the independent-particle approximation (IPA); and within the IPA using the dielectric response $\boldsymbol{\varepsilon}(E)$ at the measurement laser wavelength λ = 532 nm (E = 2.33 eV).

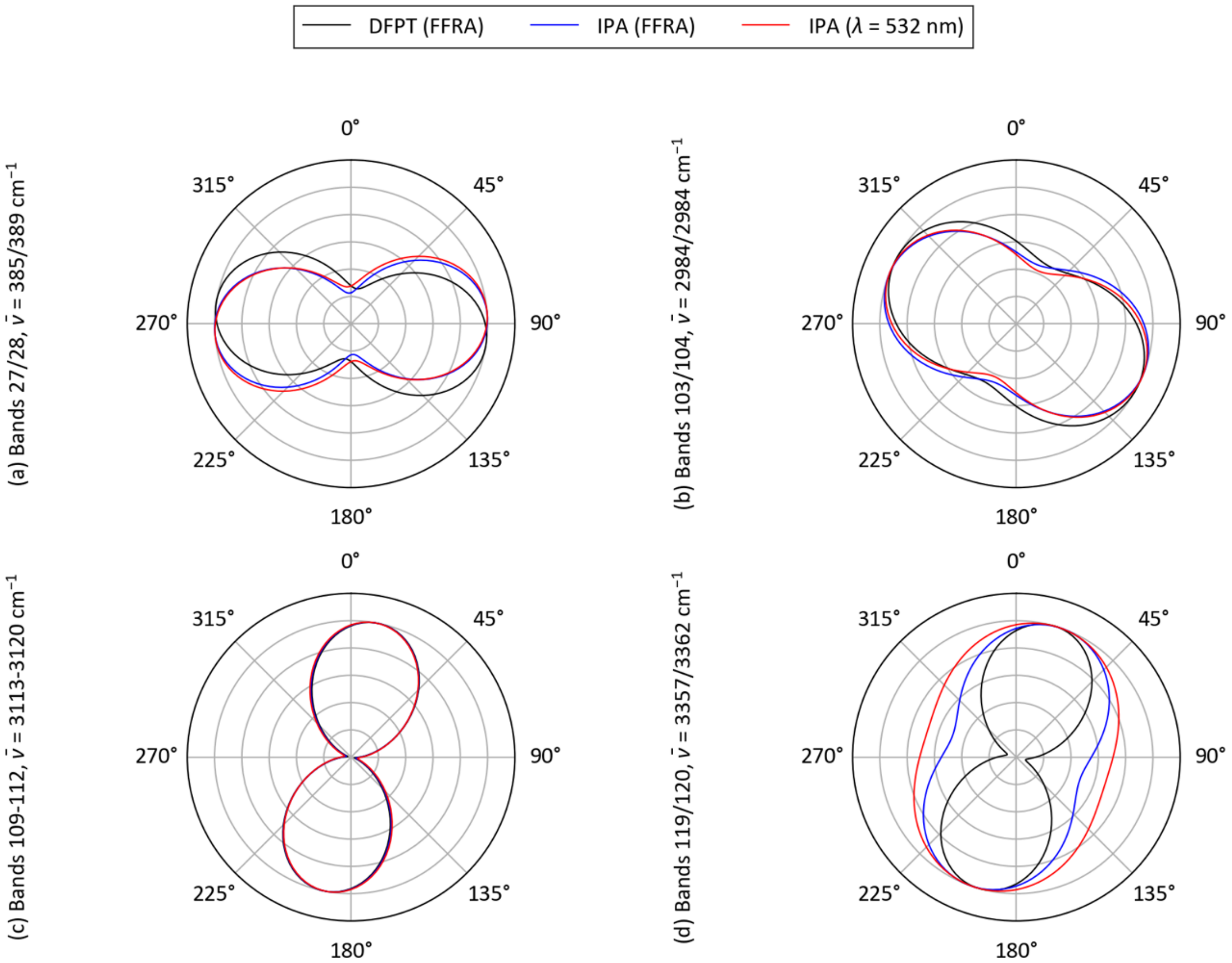

Figure S24. Predicted angle dependence of the relative intensities of selected spectral features in angle-resolved polarised Raman measurements of the (011) surface of paracetamol obtained with one of three methods: within the far-from-resonance approximation (FFRA) using high-frequency dielectric constants $\boldsymbol{\varepsilon}_{\infty}$ from density-functional perturbation theory (DFPT) including local-field effects; within the FFRA using $\boldsymbol{\varepsilon}_{\infty}$ from the independent-particle approximation (IPA); and within the IPA using the dielectric response $\boldsymbol{\varepsilon}(E)$ at the measurement laser wavelength λ = 532 nm (E = 2.33 eV).

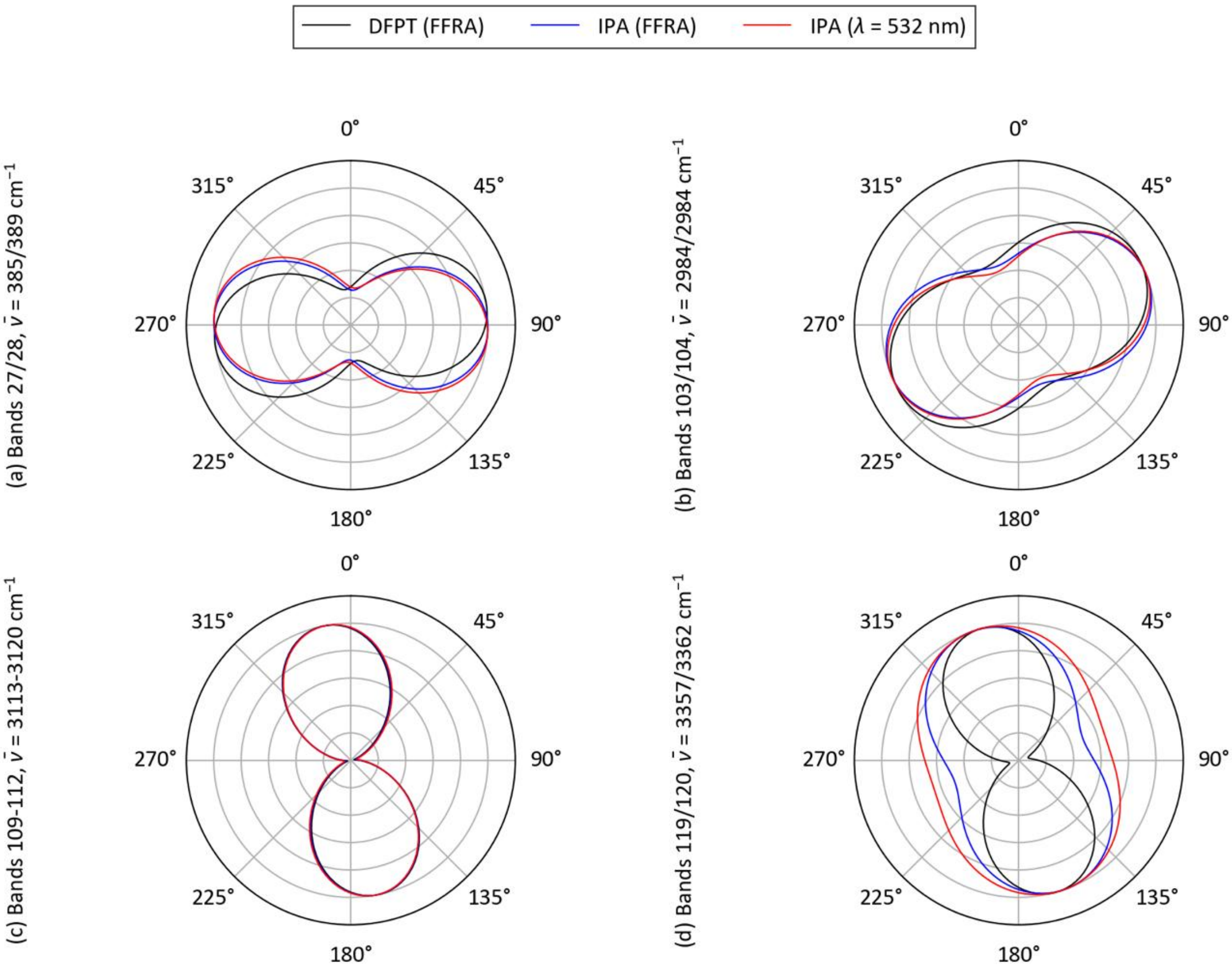

Figure S25. Predicted angle dependence of the relative intensities of selected spectral features in angle-resolved polarised Raman measurements of the $(0\bar{1}1)$ surface of paracetamol obtained with one of three methods: within the far-from-resonance approximation (FFRA) using high-frequency dielectric constants $\boldsymbol{\varepsilon}_\infty$ from density-functional perturbation theory (DFPT) including local-field effects; within the FFRA using $\boldsymbol{\varepsilon}_\infty$ from the independent-particle approximation (IPA); and within the IPA using the dielectric response $\boldsymbol{\varepsilon}(E)$ at the measurement laser wavelength λ = 532 nm (E = 2.33 eV).

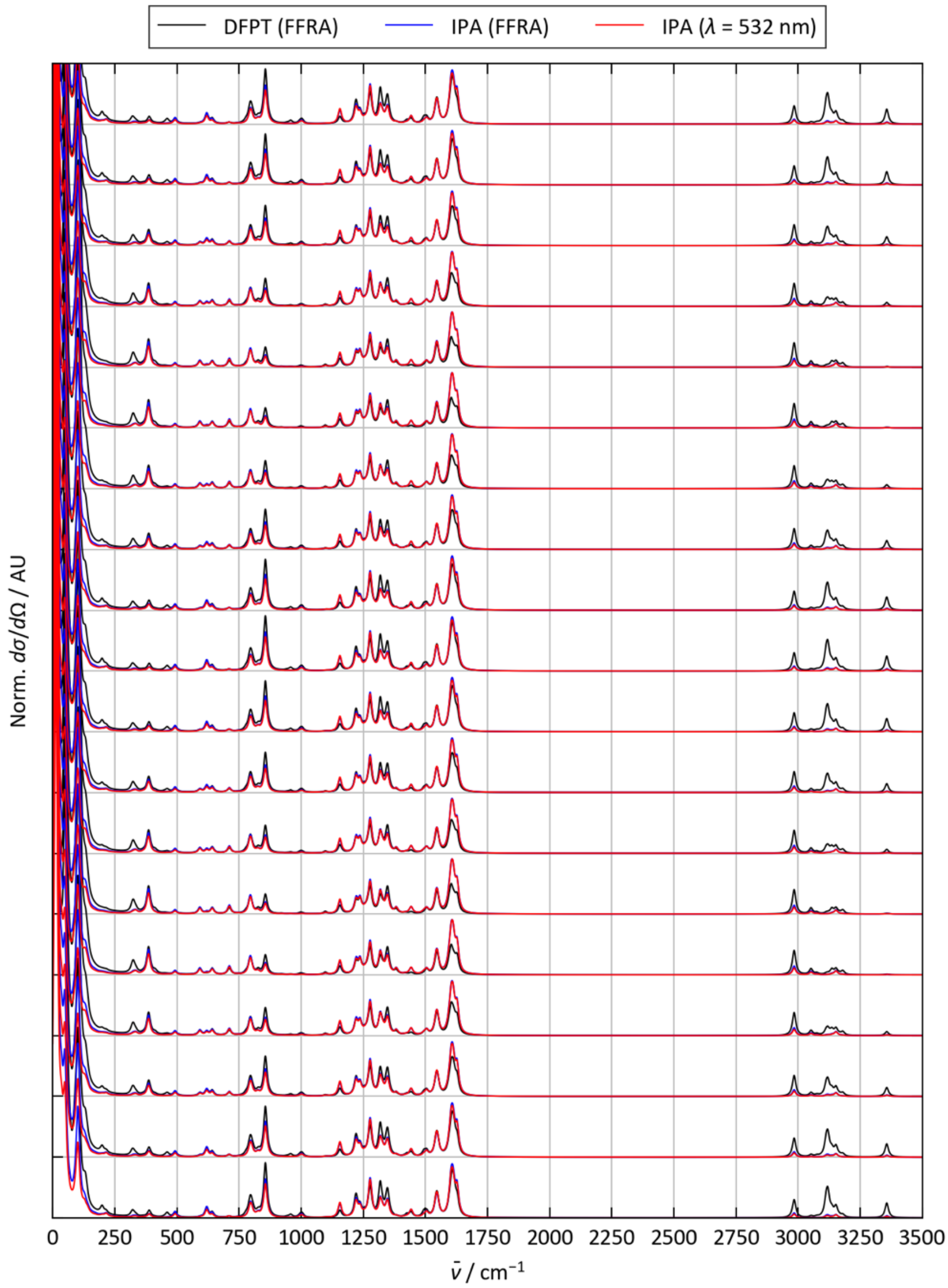

Figure S26. Predicted angle-resolved polarised Raman spectra of the (001) surface of paracetamol for in-plane rotation angles ϕ = 0-360 ° (bottom to top) obtained using Raman-activity tensors calculated with one of three methods: within the far-from-resonance approximation (FFRA) using high-frequency dielectric constants $\boldsymbol{\varepsilon}_{\infty}$ from density-functional perturbation theory (DFPT) including local-field effects; within the FFRA using $\boldsymbol{\varepsilon}_{\infty}$ from the independent-particle approximation (IPA); and within the IPA using the dielectric response $\boldsymbol{\varepsilon}(E)$ at the measurement laser wavelength λ = 532 nm (E = 2.33 eV).

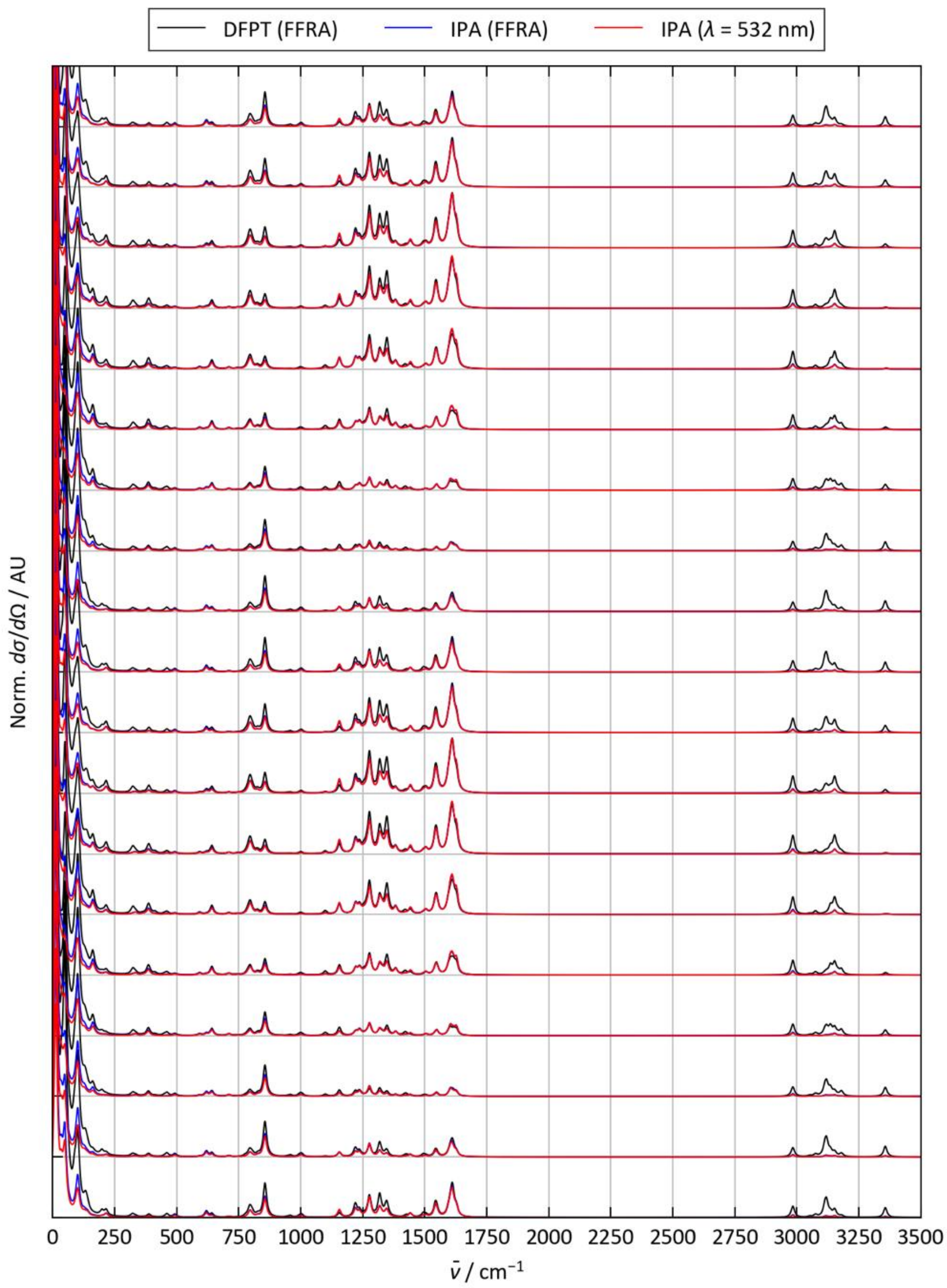

Figure S27. Predicted angle-resolved polarised Raman spectra of the (011) surface of paracetamol for in-plane rotation angles ϕ = 0-360 ° (bottom to top) obtained using Raman-activity tensors calculated with one of three methods: within the far-from-resonance approximation (FFRA) using high-frequency dielectric constants $\boldsymbol{\varepsilon}_\infty$ from density-functional perturbation theory (DFPT) including local-field effects; within the FFRA using $\boldsymbol{\varepsilon}_\infty$ from the independent-particle approximation (IPA); and within the IPA using the dielectric response $\boldsymbol{\varepsilon}(E)$ at the measurement laser wavelength λ = 532 nm (E = 2.33 eV).

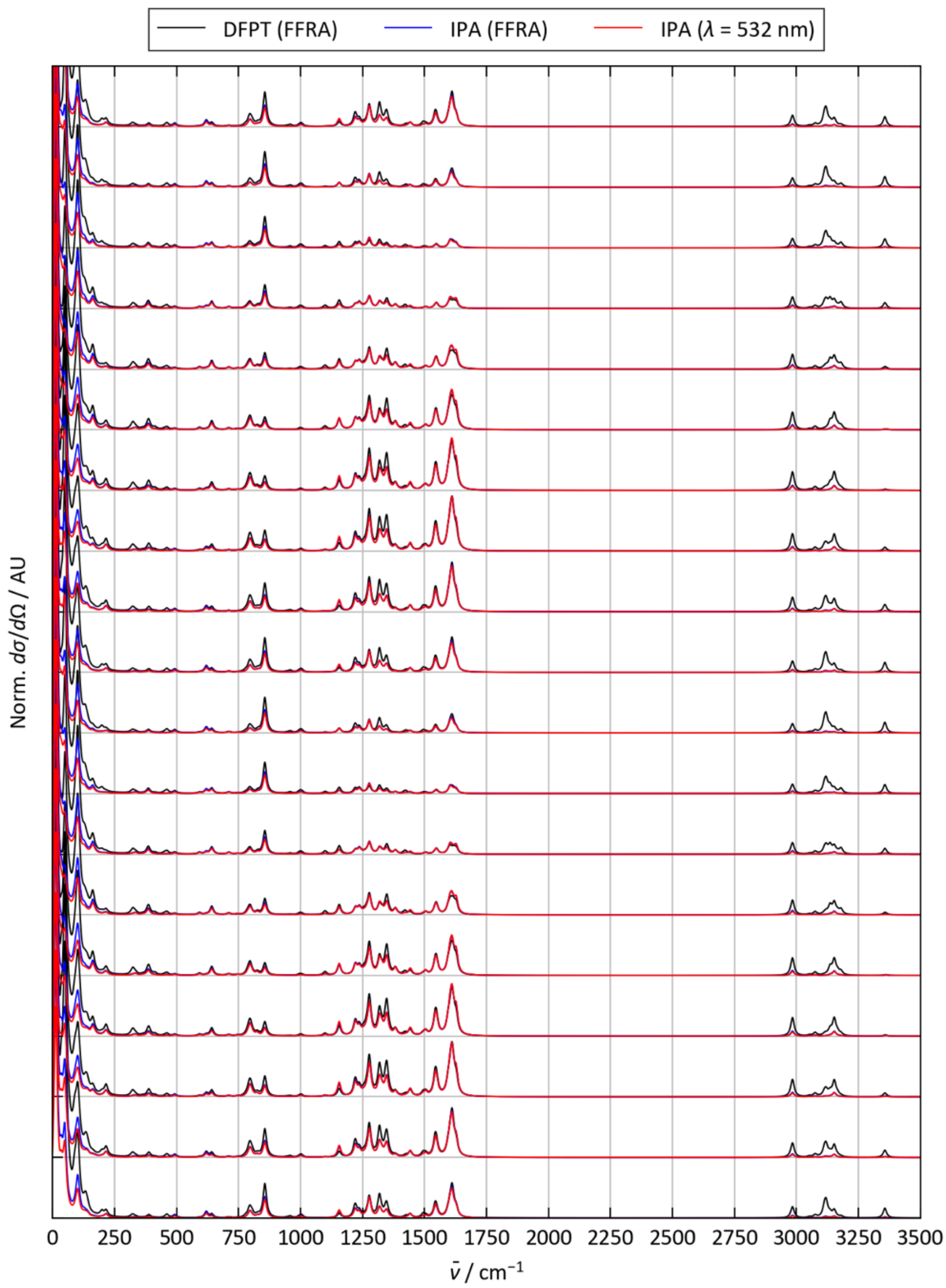

Figure S28. Predicted angle-resolved polarised Raman spectra of the $(0\bar{1}1)$ surface of paracetamol for in-plane rotation angles ϕ = 0-360 ° (bottom to top) obtained using Raman-activity tensors calculated with one of three methods: within the far-from-resonance approximation (FFRA) using high-frequency dielectric constants $\boldsymbol{\varepsilon}_{\infty}$ from density-functional perturbation theory (DFPT) including local-field effects; within the FFRA using $\boldsymbol{\varepsilon}_{\infty}$ from the independent-particle approximation (IPA); and within the IPA using the dielectric response $\boldsymbol{\varepsilon}(E)$ at the measurement laser wavelength λ = 532 nm (E = 2.33 eV).

Section S2. Surface termination effects

To reliably identify the crystal facets, it is key to understand the positions and orientations of the various functional groups on the facet as well as those that could potentially cover the surface. The (001) facet for example can be represented with 10 possible functional group terminations. Due to the centrosymmetric nature of the space group paracetamol Form I presents itself in, it is safe to assume that (001) is identical to $(00\bar{1})$ for example. The (001), $(011)$and $(01\bar{1})$ planes were visualized on the CCDC Mercury software to identify the possible functional group terminations. The (001) plane has two possible terminations. The first where, the benzene ring and the OH group are exposed at the surface and the second where the amide group is exposed at the surface. Similarly, $(011)$and $(01\bar{1})$ are identical, as are $(0\bar{1}1)$ and $(0\overline{11})$. The (011)plane can have part of the benzene ring and the C=O at the surface or in the second scenario have the OH as well as the C=O-CH_3 groups on the surface. This possibility presents OH in two different directions at the surface. The first near parallel to the normal to the plane i.e., the incident laser and the second much more inclined at almost 45 deg. This is worth mentioning as these positions affect how they interact with the polarised laser and hence the net intensity of the peaks observed from this plane. Interestingly the $(01\bar{1})$ plane has only one possible termination where the functional group presented are the OH (near parallel the normal), the C=O and the CH_3 of the amide at a significant angle to the normal but pointing at opposite directions to each other and finally a C-H bond of the benzene ring.
